# Supplementary material for: Biomarkers of response to neoadjuvant palbociclib plus anastrozole in endocrine-resistant estrogen receptor-positive/HER2-negative breast cancer: a phase 2 trial
Source: Nat Commun. 2026 Jan 27;17:949. doi: 10.1038/s41467-026-68570-6 (PMC12848104; doi:10.1038/s41467-026-68570-6)
Supplement: Supplementary file 1 — Supplementary Information [file 41467_2026_68570_MOESM1_ESM.pdf]

SUPPLEMENTARY INFORMATION

Biomarkers of response to neoadjuvant palbociclib plus anastrozole in endocrine-resistant estrogen receptor-positive/HER2-negative breast cancer: a phase 2 trial

Tim Kong[1,2,3,4], Alex Mabry[1,2], Maureen Highkin[1], Anthony Z. Wang[5], Jeremy Hoog[1], Zhanfang Guo[1], Adrian Gonzales-Gonzales[1], Shana Thomas[1], Yingduo Song[1], Feng Gao[6], Mateusz Opyrchal[1], Lindsay Peterson[1], Foluso Ademuyiwa[1], Julie Margenthaler[7], Rebecca Aft[7], Katherine Glover-Collins[7], Leslie Nehring[1], Yu Tao[6], Souzan Sanati[8], Ian S. Hagemann[8], Fouad Boulos[8], Matthew Holt[9], Li Ding[1], Wenge Zhu[10], Stephen T. Oh[3,8,11], Jianxin Wang[12], Agnieszka K. Witkiewicz[12,13], Erik S. Knudsen[12], Ron Bose[1], Jason D. Weber[1], Matthew Goetz[14], Donald Northfelt[15], Jingqin Luo[6,*], Cynthia X. Ma[1,*]

[1]Divison of Oncology, Department of Medicine, Washington University School of Medicine, St. Louis, MO
[2]Cancer Biology Graduate Program, Division of Biology and Biomedical Sciences, Washington University School of Medicine, St. Louis, MO, Department of Medicine
[3]Divison of Hematology, Department of Medicine, Washington University School of Medicine, St. Louis, MO
[4]Department of Medicine, Weill Cornell Medicine, New York, NY, USA
[5]Department of Neurosurgery, Massachusetts General Hospital, Harvard Medical School, Boston, MA
[6]Division of Public Health Science, Department of Surgery, Washington University School of Medicine, St. Louis, MO
[7]Section of Endocrine and Oncologic Surgery, Department of Surgery, Washington University School of Medicine, St. Louis, MO
[8]Department of Pathology and Immunology, Washington University School of Medicine, St. Louis, MO
[9]Baylor College of Medicine, Houston, TX
[10]Department of Biochemistry and Molecular Medicine, George Washington University School of Medicine and Health Sciences, Washington, DC
[11]Bursky Center for Human Immunology & Immunotherapy, Washington University School of Medicine, St. Louis, MO
[12]Department of Molecular and Cellular Biology, Roswell Park Comprehensive Cancer Center, Buffalo, NY
[13]Department of Pathology, Roswell Park Comprehensive Cancer Center, Buffalo, NY
[14]Department of Medical Oncology, Mayo Clinic, Rochester, MN
[15]Division of Hematology and Medical Oncology, Mayo Clinic, Phoenix, AZ

*These authors jointly supervised this work

Supplementary Figures 1-6
Supplementary Tables 1-2
TRIPOD Checklist
Bootstrapping information
Code
NeoPalAna Clinical Trial Protocol

Supplementary Fig. 1

a

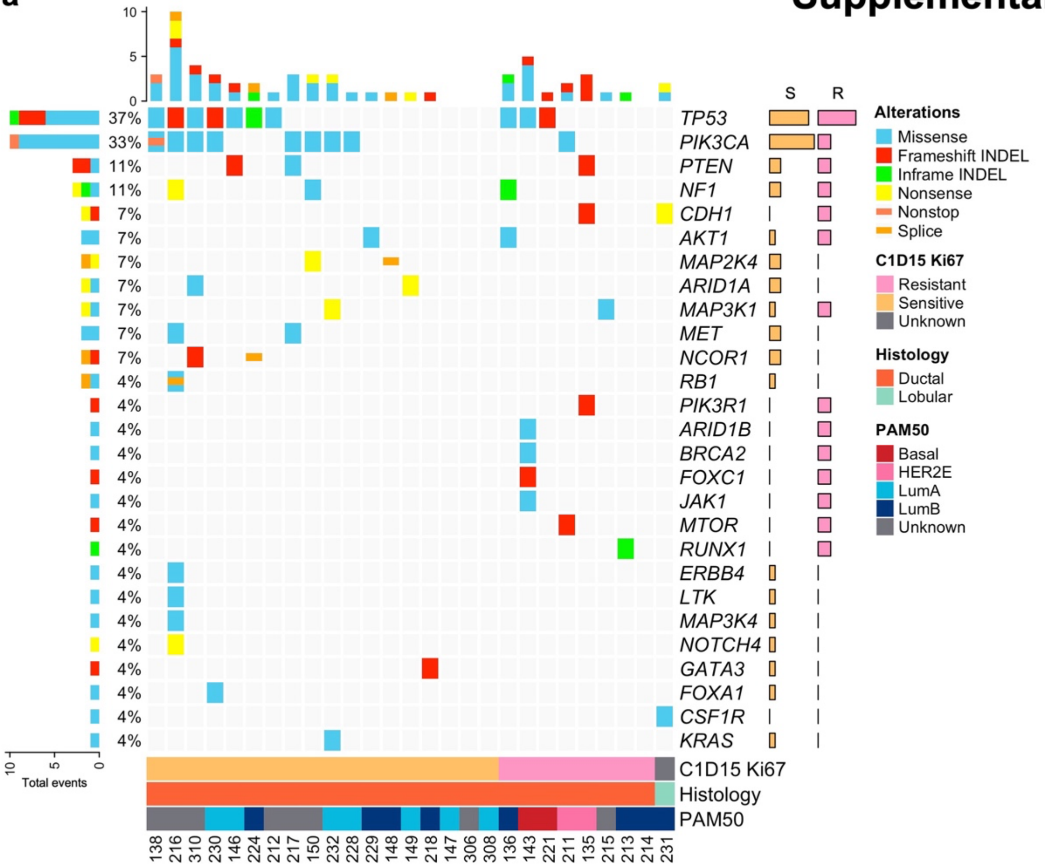

b

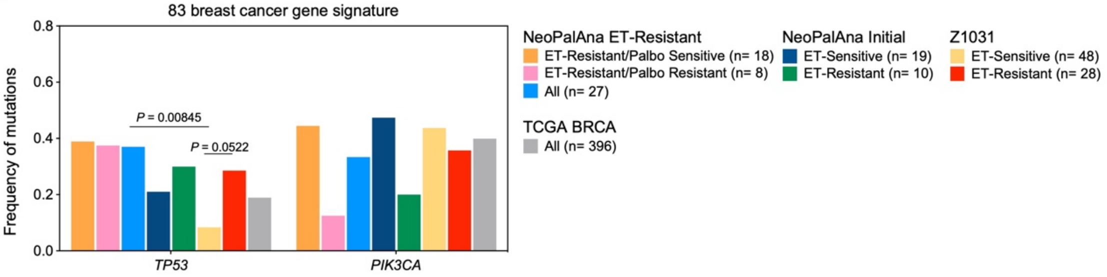

c

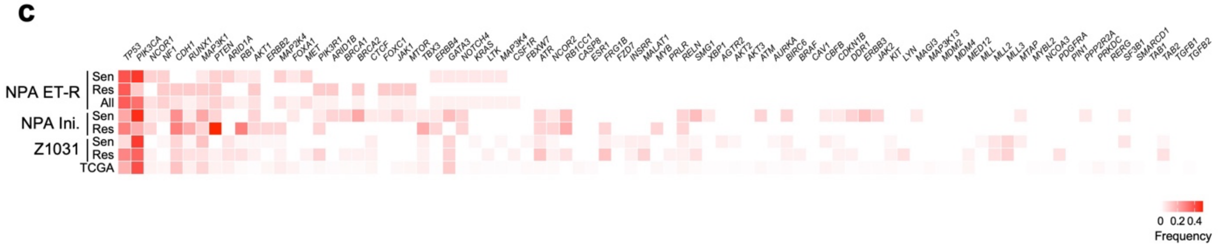

Supplementary Fig. 1. Genomic landscape of the NeoPalAna ET-R cohort. a) OncoPrint of genes in the 83-gene panel with mutations detected in at least 4% samples are shown. Left bar graph annotations: frequency of mutations seen in the cohort (n = 27 samples). Right bar graph annotations: S (Sensitive, $Ki67_{C1D15}$ ≤10%) to anastrozole plus palbociclib (ANA/PAL); R (Resistant, $Ki67_{C1D15}$ >10%) to ANA/PAL. Frequency of alterations in R/S samples within all S (n = 18) or R (n = 8) samples. **b**) Mutation frequencies of *TP53* and *PIK3CA* in the 83-gene panel identified in the NeoPalAna ET-R cohort, ET-Sensitive or ET-Resistant cases from the NeoPalAna Initial Cohort or the Z1031_POL trials, and the TCGA BRCA cohort. Statistics by Chi-square test. **c**) Mutation frequencies of all genes from the 83-gene panel across the breast cancer cohorts.

Supplementary Fig. 2

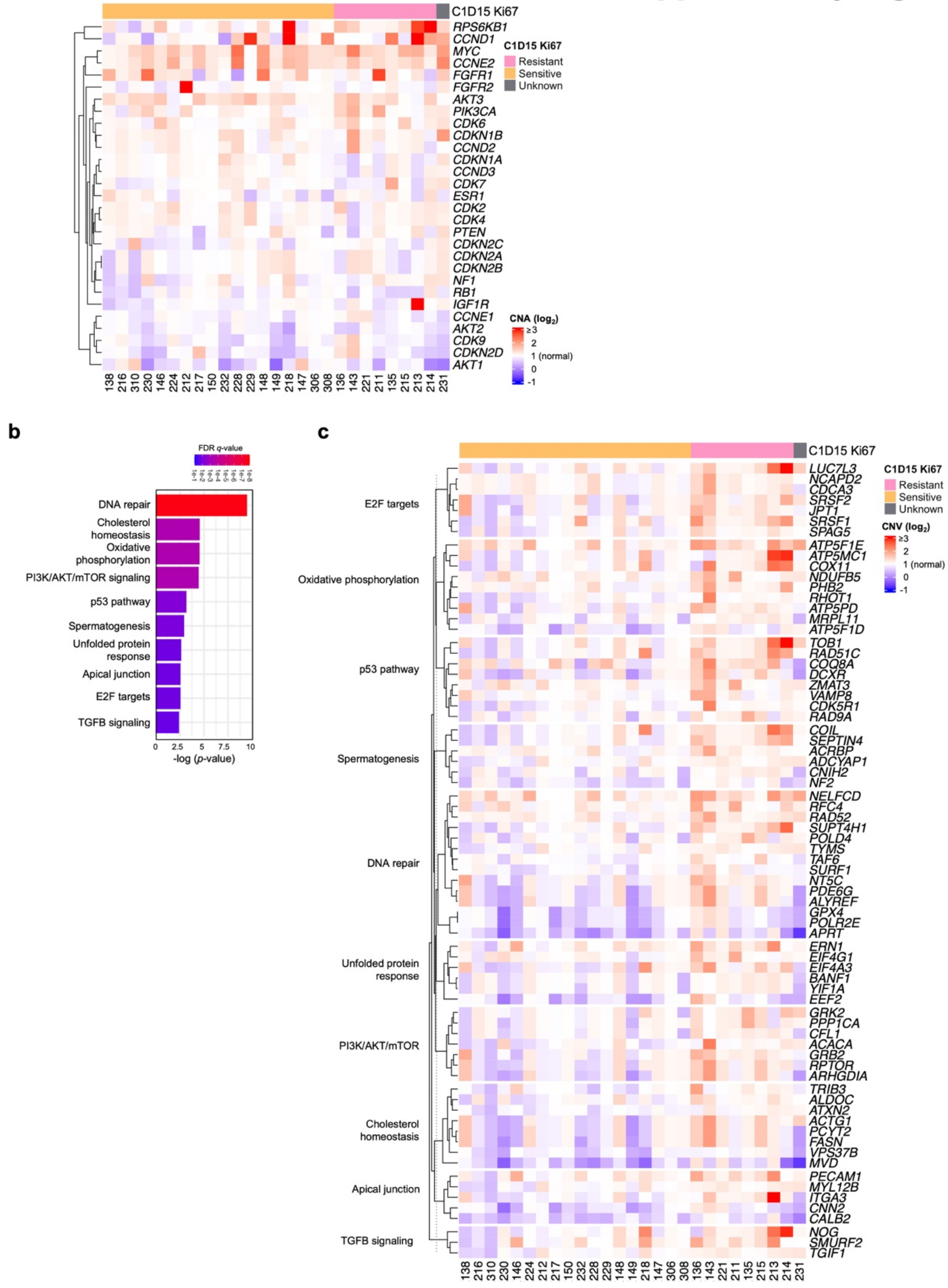

Supplementary Fig. 2. Differential copy number variations of genes between Sensitive and Resistant tumors. a) Copy number variations (CNV) of cell cycle genes and other genes of interest. Values expressed as log2(x), where a normal copy number of "2" has a transformed value of 1. **b**) Hallmark pathway enrichment of CNV for the Resistant cohort. **c**) CNV of other genes as reflected in panel b.

Supplementary Fig. 3

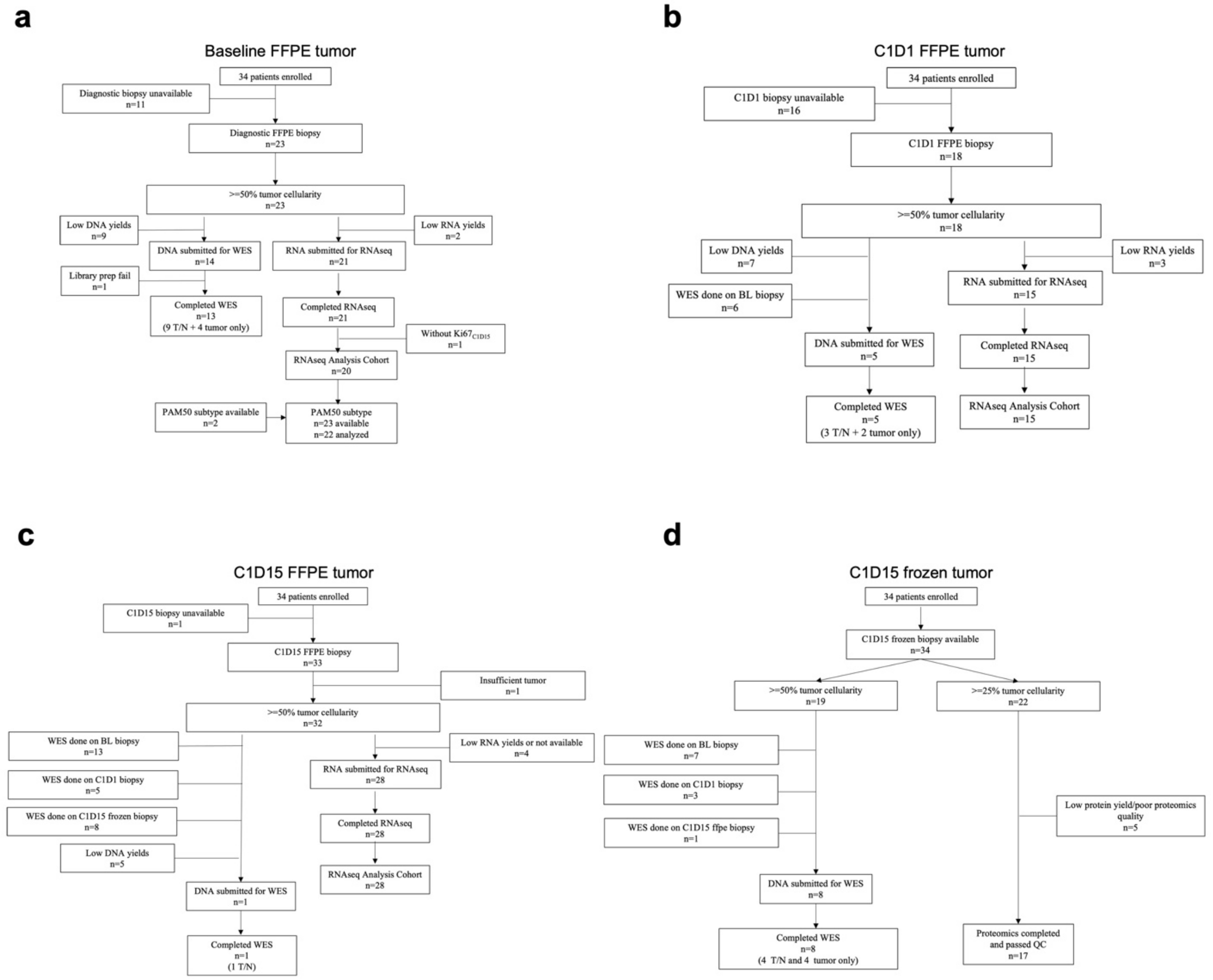

Supplementary Fig. 3. Remark diagrams. a-d) Remark Diagram of samples being analyzed at each timepoint.

Supplementary Fig. 4

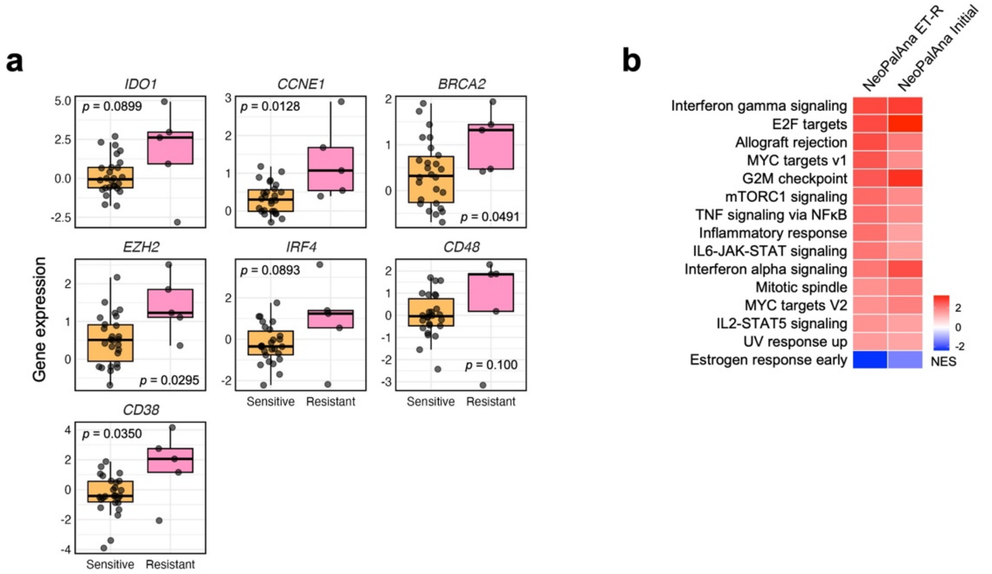

Supplementary Fig. 4. Altered expression profiles in baseline tumors from the NeoPalAna Initial cohort. a) Box plots of upregulated genes of interest observed in the NeoPalAna ET-R cohort similarly enriched in NeoPalAna Initial(tumor samples: n = 26 Sensitive; n= 5 Resistant). Statistics assessed by two-sided Mann-Whitney U test. **b**) Shared Hallmark pathway alterations in NeoPalAna ET-R and NeoPalAna Initial in ANA/PAL resistant tumors.

Supplementary Fig. 5

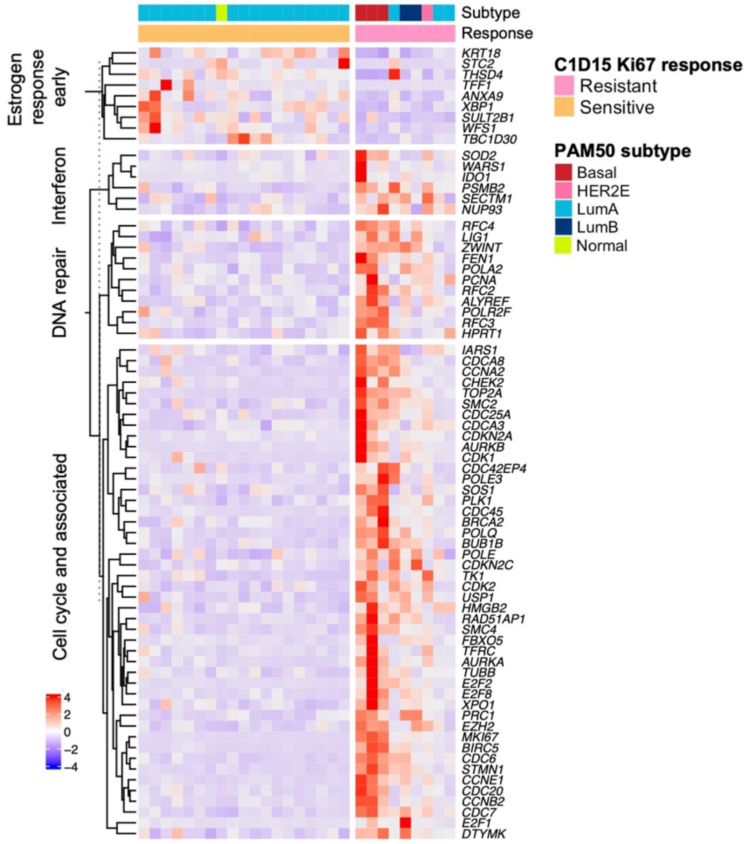

Supplementary Fig. 5. Altered expression profiles in C1D15 tumors from the NeoPalAna ET-R cohort. Among top 500 upregulated and top 500 downregulated, differentially expressed genes involved in estrogen response early, cell cycle, DNA repair, and interferon pathways.

Supplementary Fig. 6

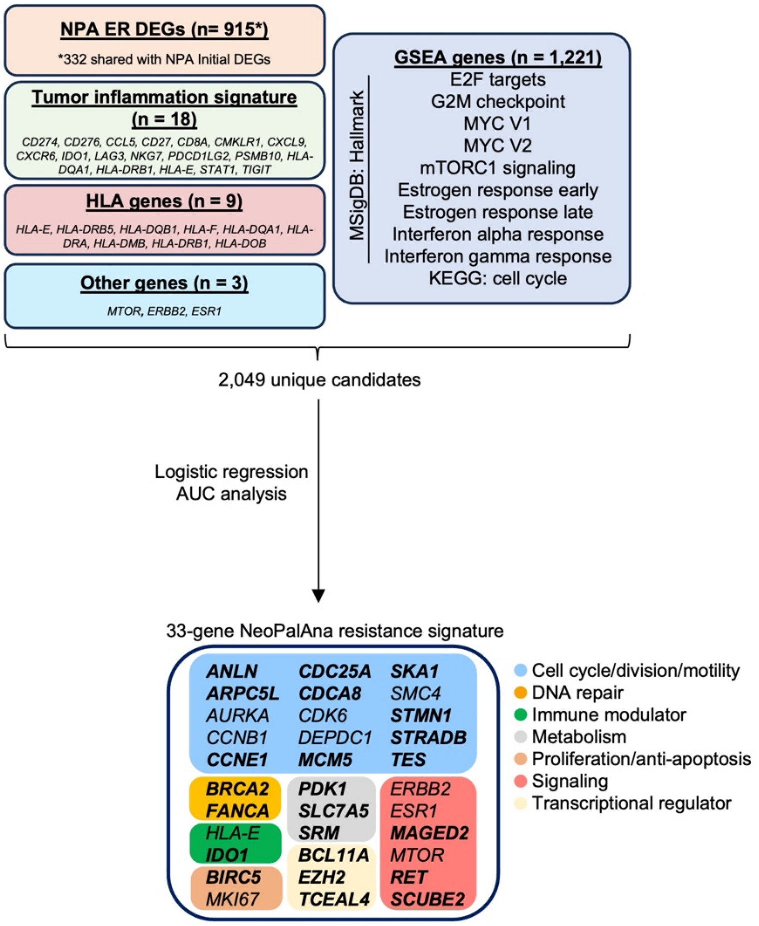

Supplementary Fig. 6. Workflow to generate the 33-gene NeoPalAna resistance signature. Candidate genes were evaluated by logistic regression and AUC analysis to derive a 33 gene signature encompassing a multitude of biological functions. Genes highlighted in bold represent differentially expressed genes from the NeoPalAna ET-R cohort.

Supplementary Table 1. Treatment related adverse events.

Adverse Event	Grade 1	Grade 2	Grade 3	Grade 4	Total
Neutrophil count decreased	2 (6%)	12 (35%)	13 (38%)	1 (3%)	82%
White blood cell decreased	5 (15%)	8 (24%)	3 (9%)	0	47%
Nausea	9 (26%)	1 (3%)	0	0	29%
Fatigue	5 (15%)	3 (9%)	0	0	24%
Anemia	6 (18%)	1 (3%)	0	0	21%
Hot flashes	6 (18%)	0	0	0	18%

AEs at least possibly related to the study drugs occurred in more than 15% of patients are shown.

Supplementary Table 2. Antibodies utilized in this study.

Target	Catalog Number	Dilution	Vender
B-tubulin	ab6046	1:30000	Abcam
AKT	(40D4) #2920	1:1000	Cell Signaling Technologies
Cyclin D1	(92G2) #2978	1:1000	Cell Signaling Technologies
pAKT S473	#9271	1:1000	Cell Signaling Technologies
pSTAT3 Y705	#9131	1:1000	Cell Signaling Technologies
RB	(D20) #9313	1:1000	Cell Signaling Technologies
STAT3	(124H6) #9139	1:1000	Cell Signaling Technologies
ISG15	Custom	1:1000	ISG15 antibody was a gift from Dr. Deb Lenschow

TRIPOD Checklist

Topic	Item	Checklist item	Verification (information, section, documentation)
Title	1	Identify the study as developing or evaluating the performance of a multivariable prediction model, the target population, and the outcome to be predicted.	Title identifies study as evaluating biomarkers of response to neoadjuvant palbociclib plus anastrozole in ER+/HER2− breast cancer.
Abstract	2	See TRIPOD+AI for Abstracts checklist.	Abstract includes objectives, study design, participants, outcomes, predictors, analysis, results, and conclusions.
Background	3a	Explain the healthcare context (including whether diagnostic or prognostic) and rationale for developing or evaluating the prediction model, including references to existing models.	Background: ER+/HER2− BC, rationale for CDK4/6i + ET; prior resistance data (Introduction).
	3b	Describe the target population and the intended purpose of the prediction model in the context of the care pathway, including its intended users (eg, healthcare professionals, patients, public).	Target population: stage II/III ER+/HER2− BC resistant to NET (on-treatment Ki67>10%); intended purpose: biomarker discovery and therapy stratification (Methods).
	3c	Describe any known health inequalities between sociodemographic groups.	Health inequalities: Race associated with response, but small sample size; caution noted (Results).
Objectives	4	Specify the study objectives, including whether the study describes the development or validation of a prediction model (or both).	Objectives: Evaluate ANA/PAL in ET-resistant tumors; primary endpoint CCCA at C1D15; secondary: biomarker discovery (Introduction).
Data	5a	Describe the sources of data separately for the development and evaluation datasets (eg, randomised trial, cohort, routine care or registry data), the rationale for using these data, and representativeness of the data.	Data source: Multi-cohort single-arm phase II trial (NeoPalAna, NCT01723774) (Introduction/Methods).
	5b	Specify the dates of the collected participant data, including start and end of participant accrual; and, if applicable, end of follow-up.	Dates: Accrual Aug 2016 – Mar 2021 (Results).
Participants	6a	Specify key elements of the study setting (eg, primary care, secondary care, general population) including the number and location of centres.	Setting: Neoadjuvant trial, academic cancer center (Introduction).
	6b	Describe the eligibility criteria for study participants.	Eligibility: Stage II/III, ER+, HER2–, Ki67 >10% after ≥4 weeks AI, ECOG 0–2 (Methods).
	6c	Give details of any treatments received, and how they were handled	Treatment: ANA daily, PAL 125 mg d1–21 q28d, ± goserelin, 5 cycles if C1D15 Ki67≤10% (Methods).

		during model development or evaluation, if relevant.	
Data preparation	7	Describe any data pre-processing and quality checking, including whether this was similar across relevant sociodemographic groups.	Data preparation: Central pathology review; RNA/DNA QC;(Methods).
Outcome	8a	Clearly define the outcome that is being predicted and the time horizon, including how and when assessed, the rationale for choosing this outcome, and whether the method of outcome assessment is consistent across sociodemographic groups.	Outcome: CCCA (Ki67 ≤2.7%) at C1D15; surrogate for anti-proliferative response (Methods).
	8b	If outcome assessment requires subjective interpretation, describe the qualifications and demographic characteristics of the outcome assessors.	Outcome assessors: Central pathology lab, blinded imaging analysis (Methods).
	8c	Report any actions to blind assessment of the outcome to be predicted.	Central Ki67 testing minimized bias and blinded analysis for the validation metastatic cohort (Methods).
Predictors	9a	Describe the choice of initial predictors (eg, literature, previous models, all available predictors) and any pre-selection of predictors before model building.	Predictors: Based on NeoPalAna cohorts, literature (Methods).
	9b	Clearly define all predictors, including how and when they were measured (and any actions to blind assessment of predictors for the outcome and other predictors).	Predictor definition: Grade, Ki67, PAM50, genomic alterations, pathways (Methods/Results).
	9c	If predictor measurement requires subjective interpretation, describe the qualifications and demographic characteristics of the predictor assessors.	Assessors: Board-certified pathologists; genomic/proteomic specialists (Methods).
Sample size	10	Explain how the study size was arrived at (separately for development and evaluation) and justify that the study size was sufficient to answer the research question. Include details of any sample size calculation.	Sample size: Simon two-stage design; target 37; closed at 34 when endpoint met (Methods).
Missing data	11	Describe how missing data were handled. Provide reasons for omitting any data.	Missing data: One patient excluded (insufficient tumor); (Results).
Analytical methods	12a	Describe how the data were used (e.g., for development and evaluation of model performance) in the analysis, including whether the data were partitioned, considering any sample size requirements.	Data used: Signature developed in NeoPalAna cohorts, validated in metastatic dataset (Methods).
	12b	Depending on the type of model, describe how predictors were	Predictors handled: RNA-seq normalization (Methods).

		handled in the analyses (functional form, rescaling, transformation, or any standardisation).	
	12c	Specify the type of model, rationale , all model-building steps, including any hyperparameter tuning, and method for internal validation.	Model: Logistic regression; ROC AUC; external validation (Methods).
	12d	Describe if and how any heterogeneity in estimates of model parameter values and model performance was handled and quantified across clusters (e.g., hospitals, countries). See TRIPOD-Cluster for additional considerations.	Heterogeneity: Analyzed by subtype, grade, race (Results).
	12e	Specify all measures and plots used (and their rationale) to evaluate model performance (e.g., discrimination, calibration, clinical utility) and, if relevant, to compare multiple models.	Performance metrics: ROC, AUC, K-M survival curves (Results).
	12f	Describe any model updating (e.g., recalibration) arising from the model evaluation, either overall or for particular sociodemographic groups or settings.	Model updating: None (Methods/Results).
	12g	For model evaluation, describe how the model predictions were calculated (e.g., formula, code, object, application programming interface).	Predictions: Signature stratified into tertiles; survival compared (Methods). Code in Supplementary Data.
Class imbalance	13	If class imbalance methods were used, state why and how this was done, and any subsequent methods to recalibrate the model or the model predictions.	Class imbalance: No formal correction; validation mitigated risk (Methods/Results).
Fairness	14	Describe any approaches that were used to address model fairness and their rationale.	Fairness: Race association noted; small numbers (Results).
Model output	15	Specify the output of the prediction model (e.g., probabilities, classification). Provide details and rationale for any classification and how the thresholds were identified.	Model output: Binary classification (Sensitive vs Resistant); risk tertiles (Method/Results).
Training versus evaluation	16	Identify any differences between the development and evaluation data in healthcare setting, eligibility criteria, outcome, and predictors.	Development vs eval: Training in NeoPalAna cohorts, validation in real-world metastatic dataset (Methods).
Ethical approval	17	Name the institutional research board or ethics committee that approved the study and describe the participant-informed consent or the ethics committee waiver of informed consent.	Ethics: IRB approval; Declaration of Helsinki; informed consent (Methods).

Funding	18a	Give the source of funding and the role of the funders for the present study.	Funding: NIH, BCRF, Pfizer, Komen, institutional grants (Acknowledgments).
Conflicts of interest	18b	Declare any conflicts of interest and financial disclosures for all authors.	Conflicts: Declared for CXM and ISH (Competing Interests).
Protocol	18c	Indicate where the study protocol can be accessed or state that a protocol was not prepared.	Protocol: IRB-approved, provided in the Supplementary Data.
Registration	18d	Provide registration information for the study, including register name and registration number, or state that the study was not registered.	Registration: NCT01723774 (Introduction).
Data sharing	18e	Provide details of the availability of the study data.	Data availability: Sequencing and proteomic data deposited; clinical data on request (Data Availability).
Code sharing	18f	Provide details of the availability of the analytical code.	Code availability: R code in Supplementary Data.
Patient & Public Involvement	19	Provide details of any patient and public involvement during the design, conduct, reporting, interpretation, or dissemination of the study or state no involvement.	Patient/Public involvement: None.
Participants	20a	Describe the flow of participants through the study, including the number of participants with and without the outcome and, if applicable, a summary of the follow-up time. A diagram may be helpful.	Participant flow: CONSORT-style diagram, 34 enrolled → 33 evaluable (Figure 1).
	20b	Report the characteristics overall and, where applicable, for each data source or setting, including the key dates, key predictors (including demographics), treatments received, sample size, number of outcome events, follow-up time, and amount of missing data. A table may be helpful. Report any differences across key demographic groups.	Characteristics: Table 1 baseline by response; demographics and tumor features (Results).
	20c	For model evaluation, show a comparison with the development data of the distribution of important predictors (demographics, predictors, and outcome).	Predictor distribution: Compared Sensitive vs Resistant by subtype, grade, Ki67 (Results).
Model development	21	Specify the number of participants and outcome events in each analysis (e.g., for model development, hyperparameter tuning, model evaluation).	Numbers: Training: 20 BL RNA-seq + initial cohort; Validation: 151 metastatic cases (Methods/Results).
Model	22	Provide details of the full prediction model (e.g., formula, code, object, application programming interface) to allow predictions in new individuals and to enable third-party evaluation and implementation, including any	Model details: 33-gene signature in Supplementary Table 8. Code in Supplementary Data (Results).

		restrictions to access or re-use (e.g., freely available, proprietary).	
Model performance	23a	Report model performance estimates with confidence intervals, including for any key subgroups (e.g., sociodemographic). Consider plots to aid presentation.	Performance: ROC AUC 0.967–0.992; HR ~2 for PFS validation (Results).
	23b	If examined, report results of any heterogeneity in model performance across clusters. See TRIPOD Cluster for additional details.	N/A
Model updating	24	Report the results from any model updating, including the updated model and subsequent performance.	Model updating: None.
Interpretation	25	Give an overall interpretation of the main results, including issues of fairness in the context of the objectives and previous studies.	Interpretation: CCCA in 57.6%; resistance pathways distinct; gene signature prognostic (Discussion).
Limitations	26	Discuss any limitations of the study (such as a non-representative sample, sample size, overfitting, missing data) and their effects on any biases, statistical uncertainty, and generalizability.	Limitations: Small sample; limited resistant cases; risk of overfitting (Discussion).
Usability of the model in the context of current care	27a	Describe how poor quality or unavailable input data (e.g., predictor values) should be assessed and handled when implementing the prediction model.	Implementation: Future studies needed; validation in larger cohorts (Discussion).
	27b	Specify whether users will be required to interact in the handling of the input data or use of the model, and what level of expertise is required of users.	User interaction: Requires molecular profiling; specialized expertise.
	27c	Discuss any next steps for future research, with a specific view to applicability and generalizability of the model.	Next steps: further validation studies, integration into trial design (Discussion).

Bootstrapping-based overoptimism-corrected AUC

Bootstrapping analysis was performed on markers (each individual gene, the composite signature, the signature and Ki67) to identify potential bias and to evaluate overoptimism correction for AUC. We performed the overoptimism correction via bootstrapping, with the following detailed steps:

(1) Calculate the AUC of an individual gene (or a composite) using the original data, denoted by $AUC_{original}$

(2) Generate bootstrap data sets (k=1, 2, …, K and K set at1000) by random sampling with replacement.

(3) Within each bootstrap dataset k, we recalculate the AUC of an individual gene (or composite) as AUC_k

(4) Calculate the difference between a bootstrap AUC and the original AUC as

$\Delta AUC_k = AUC_k - AUC_{original}$

(5) Calculate the averaged AUC difference across all the K bootstrapping datasets as ΔAUC. ΔAUC should presumably be negative, as we expect lower AUC from bootstrapped data as compared to using the original data.

(6) Calculate the corrected AUC by subtracting the optimism part from the original AUC: $AUC_{optimism\text{-}corrected} = AUC_{original} + \Delta AUC$

Code

Optimism AUC

```
#Packages
library(dplyr)
library(pROC)
library(readxl)

#----------------------------
# 1. Read in your data
#----------------------------
dat <- read_excel("Data.xlsx")

# Prepare response variable
dat <- dat %>% mutate(
  response = ifelse(`C1D15 ki67 response category` == "Resistant", 1, 0)
)


#----------------------------
# 2. Helper function: compute AUC safely
#----------------------------
compute_gene_auc <- function(resp, pred, gene_id) {
  ##this function computes AUC of a quantitative marker for a binary response outcome
  ## resp: a numerical vector for the outcome variable which is binary, e.g., 1=non-response and
0=response
  ##  pred: a numerical vector of values of a gene corresponding to the gene_id
  ## gene_id: a character, giving the ID of a gene
  valid <- !is.na(resp) & !is.na(pred)
  resp <- resp[valid]; pred <- pred[valid]

  if(length(unique(resp)) < 2 || var(pred) == 0) {
    return(data.frame(
      gene_id = gene_id,
      auc = NA_real_,
      threshold = NA_real_,
      sensitivity = NA_real_,
      specificity = NA_real_
    ))
  }

  roc_obj <- roc(resp, pred, quiet = TRUE)

  # Safely extract numeric AUC
  auc_val <- as.numeric(roc_obj$auc[1])

  coords_best <- coords(roc_obj, "best", ret = c("threshold", "sensitivity", "specificity"))

  # Ensure numeric extraction even if coords_best contains a list
  threshold_val   <- as.numeric(coords_best[["threshold"]][1])
```

```
  sensitivity_val <- as.numeric(coords_best[["sensitivity"]][1])
  specificity_val <- as.numeric(coords_best[["specificity"]][1])

  data.frame(
    gene_id = gene_id,
    auc = auc_val,
    threshold = threshold_val,
    sensitivity = sensitivity_val,
    specificity = specificity_val
  )
}

#----------------------------
# 3. Nonparametric AUC and bootstrapping for multiple genes
#----------------------------
correctAUC_nonparametric <- function(nboot=1000, dat, resp_id="response", gene_list=NULL,
seed=123) {

  ##This function correct the over-optimism in AUC via bootstrapping
  #nboot: a numeric value, giving number of bootstrap iterations
  #dat: a data frame, containing the response variable provided by resp_id and the gene IDs to
be evaluated provided by gene_list
  #resp_id : a character, giving the response variable name in the data
  #gene_list: a character vector, giving gene IDs
  #seed: the seed for random number generation

  set.seed(seed)
  #if gene_list is not specified, will do all variables in dat excluding resp_id
  if(!is.null(gene_list)) {
    dat <- dat %>% select(all_of(c(resp_id, gene_list)))
  } else {
    gene_list <- setdiff(names(dat), resp_id)
  }

  nobs <- nrow(dat)

  #do bootstrapping, (1)at iteration jj=0, the original data will be used, at j=1:nboot, generate
bootstrap data by random sampling with replacement
  # (2) within each bootstrap,  calcate the AUC of genes
  res <- sapply(0:nboot, function(jj) {

    if(jj == 0) {
      boot_dat <- dat #use original data
    } else {
      #generate a bootstrap data set
      set.seed(jj)
      boot_idx <- sample(1:nobs, nobs, replace = TRUE)
      boot_dat <- dat[boot_idx, ]
    }
   #generate AUC for genes under a bootstrapped data set
    sapply(gene_list, function(gene_id) {
```

```
      auc_out <- compute_gene_auc(resp = boot_dat[[resp_id]], pred = boot_dat[[gene_id]],
gene_id = gene_id)
      auc_out$auc
    })
  })

  res <- as.matrix(res) #rows are gene, columns are bootstrapping iteration with 1st column
being the AUC under the original data
  auc_original <- res[,1] #the original AUC of genes
  auc_difference <- res[,-1] - res[,1]
  auc_optimism <- apply(auc_difference, 1, mean, na.rm = TRUE)# calculate the optimism AUC
as the averaged AUC difference between AUC from bootstrapping and the original
(bootstrapped AUCs are presumably lower than original AUC and a negative difference)
  auc_corrected <- res[,1] + auc_optimism # calculate the optimism corrected AUC by
subtracting the optimism

  out <- data.frame(
    gene_id = names(auc_optimism),
    auc_original = auc_original,
    auc_corrected = auc_corrected,
    auc_optimism = auc_optimism
  )

  return(list(out = out, boot_res = res))
}

#-----------------------------
# 4. Define 33 genes
#-----------------------------
gene_list_33 <- c(
  "ANLN","ARPC5L","AURKA","BCL11A","BIRC5","BRCA2","CCNB1","CCNE1",
  "CDC25A","CDCA8","CDK6","DEPDC1","ERBB2","ESR1","EZH2","FANCA",
  "HLAE","IDO1","MAGED2","MCM5","MKI67","MTOR","PDK1","RET",
  "SCUBE2","SKA1","SLC7A5","SMC4","SRM","STMN1","STRADB","TCEAL4","TES"
)

#-----------------------------
# 5. Run bootstrap-corrected AUC
#-----------------------------
out <- correctAUC_nonparametric(
  nboot = 1000,
  dat = dat,
  resp_id = "response",
  gene_list = gene_list_33,
  seed = 123
)

#-----------------------------
# 6. Export results
#-----------------------------
write.csv(out$out, "Reults.csv", row.names = FALSE)
```

AUC

```
# Packages
library(readxl)
library(tidyr)
library(dplyr)
library(nsprcomp)
library(pROC)

# Create output folder
dir.create("results2", showWarnings = FALSE)

## (1) Read in data -------------------------------------------------------
dat <- read_excel("Data.xlsx")

# Separate clinical and gene data
clinic <- dat %>% select(`Sample PPI`:`C1D15 ki67 response category`)
gene <- dat %>% select(-(`Sample PPI`:`C1D15 ki67 response category`)) %>% as.matrix()

## (2) Sparse PCA ---------------------------------------------------------
n_PC <- 10
pca.res <- nsprcomp(gene, ncomp = n_PC, nneg = FALSE, center = TRUE, scale. = TRUE)

# Scree plot
plot(pca.res)

# Add PC scores to data
pc <- pca.res$x
colnames(pc) <- paste0("PC", 1:n_PC)
dat <- bind_cols(dat, pc)


## (3) Add key variables (safe mutate) -------------------------------------
dat <- dat %>%
  mutate(
    response = ifelse(`C1D15 ki67 response category` == "Resistant", 1, 0),
    Ki67 = `Baseline Ki67`,
    Subtype = if ("Subtype" %in% colnames(.))
      ifelse(Subtype == "LumA", "LumA", "nonLumA") else NA
  )

write.csv(dat, file = "results2/Results.csv", row.names = FALSE)

## (4) ROC logistic function (bulletproof) --------------------------------
ROC_logistic <- function(dat0, markerID = "PC1", out_prefix = "results2/ROC", save=TRUE) {
  #dat0: a data frame, containing markers as specified in markerID and response variables
(under "response")
  #markerID: a character, indicating the marker to be evaluated for AUC
  #out_prefix: a character, the output folder
  #save: a logic indicating whether to save the ROC output and plot (set to FALSE if doing
bootstrapping)
```

```
  # Create missing columns if needed
  if (!"response" %in% colnames(dat0)) {
    stop("No 'response' column found. Please create it before running.")
  }
  if (!"Ki67" %in% colnames(dat0)) {
    stop("No 'Ki67' column found. Please create it before running.")
  }

  fmt_auc <- function(auc_ci) {
    sprintf("%.3f (%.3f, %.3f)", auc_ci[2], auc_ci[1], auc_ci[3])
  }

  # Fit models
  ki67_mod <- glm(response ~ Ki67, family = binomial, data = dat0)
  marker_mod <- glm(as.formula(paste("response ~", markerID)), family = binomial, data = dat0)
  ki67_marker_mod <- glm(as.formula(paste("response ~ Ki67 +", markerID)), family = binomial,
data = dat0)

  # Predictions
  ki67_pred <- predict(ki67_mod, type = "response")
  marker_pred <- predict(marker_mod, type = "response")
  ki67_marker_pred <- predict(ki67_marker_mod, type = "response")

  # ROC curves
  ki67_roc <- roc(dat0$response, ki67_pred)
  marker_roc <- roc(dat0$response, marker_pred)
  ki67_marker_roc <- roc(dat0$response, ki67_marker_pred)

  # AUC
  ki67_auc <- ci.auc(ki67_roc, method = "delong")
  marker_auc <- ci.auc(marker_roc, method = "delong")
  ki67_marker_auc <- ci.auc(ki67_marker_roc, method = "delong")

  # Save coefficients
  if(save)
  {
    write.csv(summary(ki67_mod)$coef, file = paste0(out_prefix, "_Ki67only.csv"))
    write.csv(summary(marker_mod)$coef, file = paste0(out_prefix, "_PConly.csv"))
    write.csv(summary(ki67_marker_mod)$coef, file = paste0(out_prefix, "_Ki67_PC.csv"))
  }

  # Save ROC plot
  if(save) jpeg(paste0(out_prefix, "_plot.jpeg"), res = 300, height = 6, width = 8, units = "in")
  plot(ki67_roc, col = 1, main = "ROC curves", xlim = c(1, 0))
  text(0.45, 0.3, col = 1, labels = paste("Baseline Ki67:", fmt_auc(ki67_auc)), cex = 1.65)

  plot(marker_roc, col = 2, add = TRUE, lwd = 2, lty = 2)
  text(0.45, 0.2, col = 2, labels = paste("PC:", fmt_auc(marker_auc)), cex = 1.65)

  plot(ki67_marker_roc, col = 4, add = TRUE)
```

```
  text(0.45, 0.1, col = 4, labels = paste("Ki67+PC:", fmt_auc(ki67_marker_auc)), cex = 1.65)
  if(save) dev.off()

  # Return results
  list(
    ki67_roc = ki67_roc,
    marker_roc = marker_roc,
    ki67_marker_roc = ki67_marker_roc,
    aucs = data.frame(
      Ki67 = fmt_auc(ki67_auc),
      PC = fmt_auc(marker_auc),
      Ki67_PC = fmt_auc(ki67_marker_auc)
    ),
    #add the 3 lines to return the AUC estimate alone for use in bootstrap optimism correction
    ki67_auc=ki67_auc[2],
    marker_auc=marker_auc[2],
    ki67_marker_auc=ki67_marker_auc[2]
  )
}

## (5) Run ROC analysis ---------------------------------------------------
PC1_roc <- ROC_logistic(dat0 = dat, markerID = "PC1", out_prefix = "results2/ROC_PC1")


#-----------------------------
# (6). Bootstrap-based optimism correction for ki67, marker and marker+ki67 where marker can be a composite signature or an individual gene
#-----------------------------
correctAUC_ROC_logistic <- function(nboot=1000, dat, resp_id="response", markerID="PC1", seed=123) {

  set.seed(seed)

  #nboot: a numeric value, giving number of bootstrap iterations
  #dat: a data frame, containing at least the response variable provided by resp_id, ki67, and the marker variable provided by markerID to be evaluated
  #resp_id : a character, giving the response variable name in the data
  #markerID: a character, giving the marker ID for evaluation

  #if gene_list is not specified, will do all variables in dat excluding resp_id
  if(!is.null(gene_list)) {
    dat <- dat %>% select(all_of(c(resp_id, gene_list)))
  } else {
    gene_list <- setdiff(names(dat), resp_id)
  }

  nobs <- nrow(dat)

  #do bootstrapping, (1)at iteration jj=0, the original data will be used, at j=1:nboot, generate bootstrap data by random sampling with replacement
  # (2) within each bootstrap,  calcate the AUC of genes
```

```
  res <- sapply(0:nboot, function(jj) {

    if(jj == 0) {
      boot_dat <- dat #use original data
    } else {
      #generate a bootstrap data set
      set.seed(jj)
      boot_idx <- sample(1:nobs, nobs, replace = TRUE)
      boot_dat <- dat[boot_idx, ]
    }
    #generate AUC for ki67, marker and ki67+marker under a bootstrapped data set
    roc_res <- ROC_logistic(dat0 = dat, markerID = markerID, out_prefix = "results2/ROC_PC1",
save=FALSE)
    c(ki67_auc=roc_res$ki67_auc, marker_auc=roc_res$marker_auc,
ki67_marker_auc=roc_res$ki67_marker_auc)
    })


  res <- as.matrix(res) #3 rows for ki67, marker and ki67+marker and columns are bootstrapping
iteration with 1st column being the AUC under the original data
  auc_original <- res[,1] #the original AUC of genes
  auc_difference <- res[,-1] - res[,1]
  auc_optimism <- apply(auc_difference, 1, mean, na.rm = TRUE)# calculate the optimism AUC
as the averaged AUC difference between AUC from bootstrapping and the original
(bootstrapped AUCs are presumably lower than original AUC and a negative difference)
  auc_corrected <- res[,1] + auc_optimism # calculate the optimism corrected AUC by
subtracting the optimism, 3 rows, coressponding to ki67, marker, ki67+marker

  out <- data.frame(
    gene_id = names(auc_optimism),
    auc_original = auc_original,
    auc_corrected = auc_corrected,
    auc_optimism = auc_optimism
  )

  return(list(out = out, boot_res = res))
}

#-----------------------------
# (7). Run bootstrap-corrected AUC for ki67, the composite signature, ki67+composite
#-----------------------------
out <- correctAUC_ROC_logistic(
  nboot = 1000,
  dat = dat,
  resp_id = "response",
  markerID = "PC1",
  seed = 123
)
```

Protocol Administrative Letter

March 25, 2020

Protocol 201301106

"A Phase II Trial of Neoadjuvant PD 0332991, a Cyclin-Dependent Kinase (Cdk) 4/6 inhibitor, in Combination with Anastrozole in Women with Clinical Stage 2 or 3 Estrogen Receptor Positive and HER2 Negative Breast Cancer"

Currently the study is open to enrollment for the Endocrine Resistant Cohort. We would like to omit the requirement of central review of Ki67 >10% reported by local clinical lab for eligibility. This modification is made to facilitate study enrollment during COVID-19 pandemics".

If you have any questions, please contact me or the regulatory coordinator (Natalie Benford at natalienconner@wustl.edu, 314-747-8365)

Regards,

Dr. Cynthia Ma, MD, PhD.
Professor of Medicine
Washington University School of Medicine
Internal Medicine – Medical Oncology
Phone: 314-362-8903
Email: cynthiaxma@wustl.edu

Protocol Update Letter

October 12, 2019

To: Protocol 201301106 investigators

Re: "*A Phase II Trial of Neoadjuvant PD 0332991, a Cyclin-Dependent Kinase (Cdk) 4/6 inhibitor, in Combination with Anastrozole in Women with Clinical Stage 2 or 3 Estrogen Receptor Positive and HER2 Negative Breast Cancer*"

Dear Investigator,

The purpose of this letter is to provide guidance for the clinical management of Interstitial Lung Disease (ILD)/pneumonitis. This guidance will also be incorporated into the protocol at the next amendment in a new sub-section (Section 6.2.2) of the palbociclib dose modifications:

6.2.2 Dose Adjustments Due to Interstitial Lung Disease (ILD)/Pneumonitis

Monitor patients for pulmonary symptoms indicative of ILD/pneumonitis (e.g. hypoxia, cough, dyspnea). In patients who have new or worsening respiratory symptoms and are suspected to have developed ILD/pneumonitis, interrupt palbociclib immediately and evaluate the patient. Permanently discontinue palbociclib in patients with severe ILD or pneumonitis.

If you have any questions, please contact myself or the primary clinic coordinator (Jill Anderson, jill.anderson@wustl.edu, 314-747-5209).

Regards,

Cynthia Ma, MD, PhD
Professor of Medicine
Washington University School of Medicine
Internal Medicine – Medical Oncology
Email: cynthiaxma@wustl.edu

A Phase II Trial of Neoadjuvant PD 0332991, a Cyclin-Dependent Kinase (Cdk) 4/6 inhibitor, in Combination with Anastrozole in Women with Clinical Stage 2 or 3 Estrogen Receptor Positive and HER2 Negative Breast Cancer

Washington University School of Medicine, Division of Oncology
660 South Euclid Avenue, Campus Box 8056, St. Louis, MO 63110

Protocol#: 201301106
Version Date: 09/14/16

Coordinating Center: **Washington University School of Medicine**

Principal Investigator: **Cynthia Ma, M.D., Ph.D.**
Washington University School of Medicine
Campus Box 8076
660 South Euclid Avenue
St. Louis MO 63110
Telephone: 314-362-9383
Fax: 314-362-7086
Email: cynthiaxma@wustl.edu

**Co-Investigators**	**Institution**	**Modality**
Matthew J. C. Ellis, M.B., Ph.D.	Baylor College of Medicine	Medical Oncology
Julie A. Margenthaler, M.D.	Washington University	Surgical Oncology
Mark Watson, M.D.	Washington University	Pathology and Immunology
Souzan Sanati, M.D.	Washington University	Anatomic and Molecular Pathology
Hussam Al-Kateb, M.Sc., Ph.D.,	Washington University	Genomics and Pathology Services
Matthew Goetz, M.D.	Mayo Clinic – Rochester	Medical Oncology
Tina Hieken, M.D.	Mayo Clinic – Rochester	Surgical Oncology
Donald W. Northfelt, M.D.	Mayo Clinic – Scottsdale	Medical Oncology
Andres Forero, M.D.	University of Alabama	Medical Oncology
Feng Gao, M.D., Ph.D.	Washington University	Biostatistics

Study Team Contact: Caroline Bumb
Phone: (314) 362-7249
Email: cbumb@ wustl.edu

Study Drug: PD 0332991 (palbociclib)
IND#: 117008
Clinical Trials.gov#: NCT01723774

CONFIDENTIAL
The information contained in this document is regarded as confidential and, except to the extent necessary to obtain informed consent, may not be disclosed to another party unless law or regulations require such disclosure. Persons to whom the information is disclosed must be informed that the information is confidential and may not be further disclosed by them

A Phase II Trial of Neoadjuvant PD 0332991, a Cyclin-Dependent Kinase (Cdk) 4/6 inhibitor, in Combination with Anastrozole in Women with Clinical Stage 2 or 3 Estrogen Receptor Positive and HER2 Negative Breast Cancer

Protocol Revision History

**Initial Approval Version**	**01/15/2013**
**Amendment #1 Version**	**03/01/2013**
**Amendment #2 Version**	**10/11/2013**
**Amendment #3 Version**	**01/29/2014**
**Amendment #4 Version**	**04/24/2014**
**Amendment #5 Version**	**09/29/2014**
**Amendment #6 Version**	**02/06/2015**
**Amendment #7 Version**	**11/25/2015**
**Amendment #8 Version**	**09/14/2016**

A Phase II Trial of Neoadjuvant PD 0332991, a Cyclin-Dependent Kinase (Cdk) 4/6 inhibitor, in Combination with Anastrozole in Women with Clinical Stage 2 or 3 Estrogen Receptor Positive and HER2 Negative Breast Cancer

Principal Investigator Signature Page

Principal Investigator:	Cynthia X. Ma, M.D., Ph.D.	
	Signature of Investigator	Date
	Printed Name of Investigator	
	By my signature, I agree to personally supervise the conduct of this study and to ensure its conduct in compliance with the protocol, informed consent, IRB/HRPO procedures, the Declaration of Helsinki, ICH Good Clinical Practices guidelines, and the applicable parts of the United States Code of Federal Regulations or local regulations governing the conduct of clinical studies.	

Contact Information

Principal Investigator:

Cynthia X. Ma, M.D., Ph.D.
Washington University School of Medicine
Division of Oncology
660 South Euclid Avenue, Campus Box 8076
St. Louis, MO 63110
Phone: (314) 362-8903
Fax: (314) 747-9320
E-mail: cynthiaxma@wustl.edu

SCHEMA

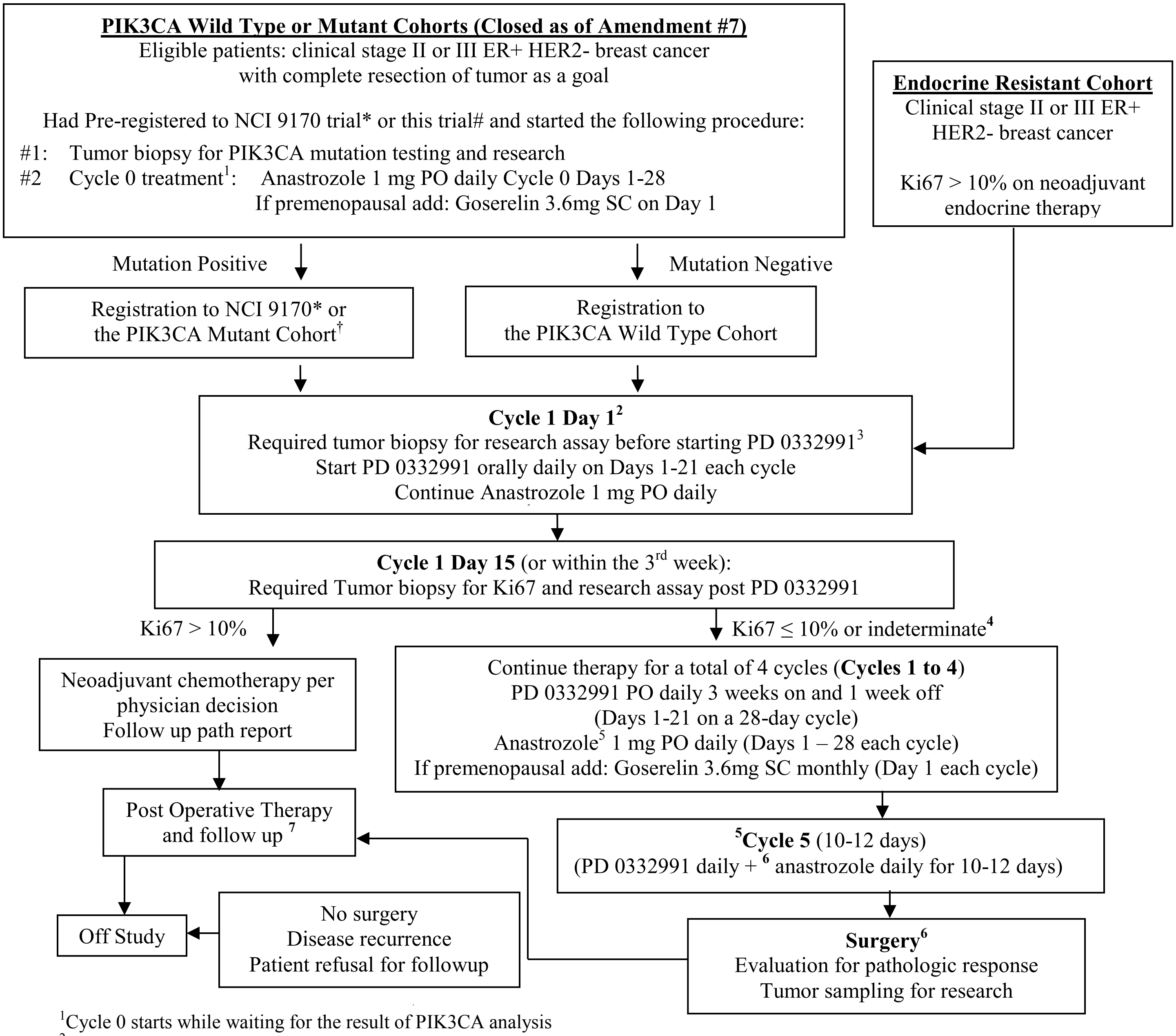

[1]Cycle 0 starts while waiting for the result of PIK3CA analysis
[2]Cycle length=28 Days
[3]Biopsy prior to PD0332991 is not required in the endocrine resistant cohort if the patient had a research tumor sample collected at the time of Ki67 analysis while on endocrine therapy
[4]Optional biopsy on Day 15 of subsequent cycles is recommended if C1D15 Ki67 was indeterminate or if not performed.
[5]Cycle 5 duration is 10-12 days and administered in patients whose absolute neutrophil is recovered to ≥ 1.5k/mcL and platelet ≥ 100k /mcL and treatment related AEs to grade 1 or less within 3 weeks after completion of cycle 4. Patients who do not meet this criteria may proceed to surgery in 3-5 weeks post cycle 4 day 21 PD0332991.
[6]Anastrozole is continued until the day of surgery. If premenopausal continue goserelin 3.6 mg SC monthly throughout the neoadjuvant duration. PD0332991 is also continued until the day of surgery in patients who started cycle 5 therapy.
[7] See Postoperative Therapy and Follow-up Schema
*NCI9170: A phase II trial of neoadjuvant MK-2206 in combination with anastrozole for PIK3CA mutant clinical stage 2 or 3 ER+ and HER2- breast cancer
†If the NCI9170 trial is not open or was open but is now closed at the participating institution

POSTOPERATIVE THERAPY AND FOLLOW-UP SCHEMA

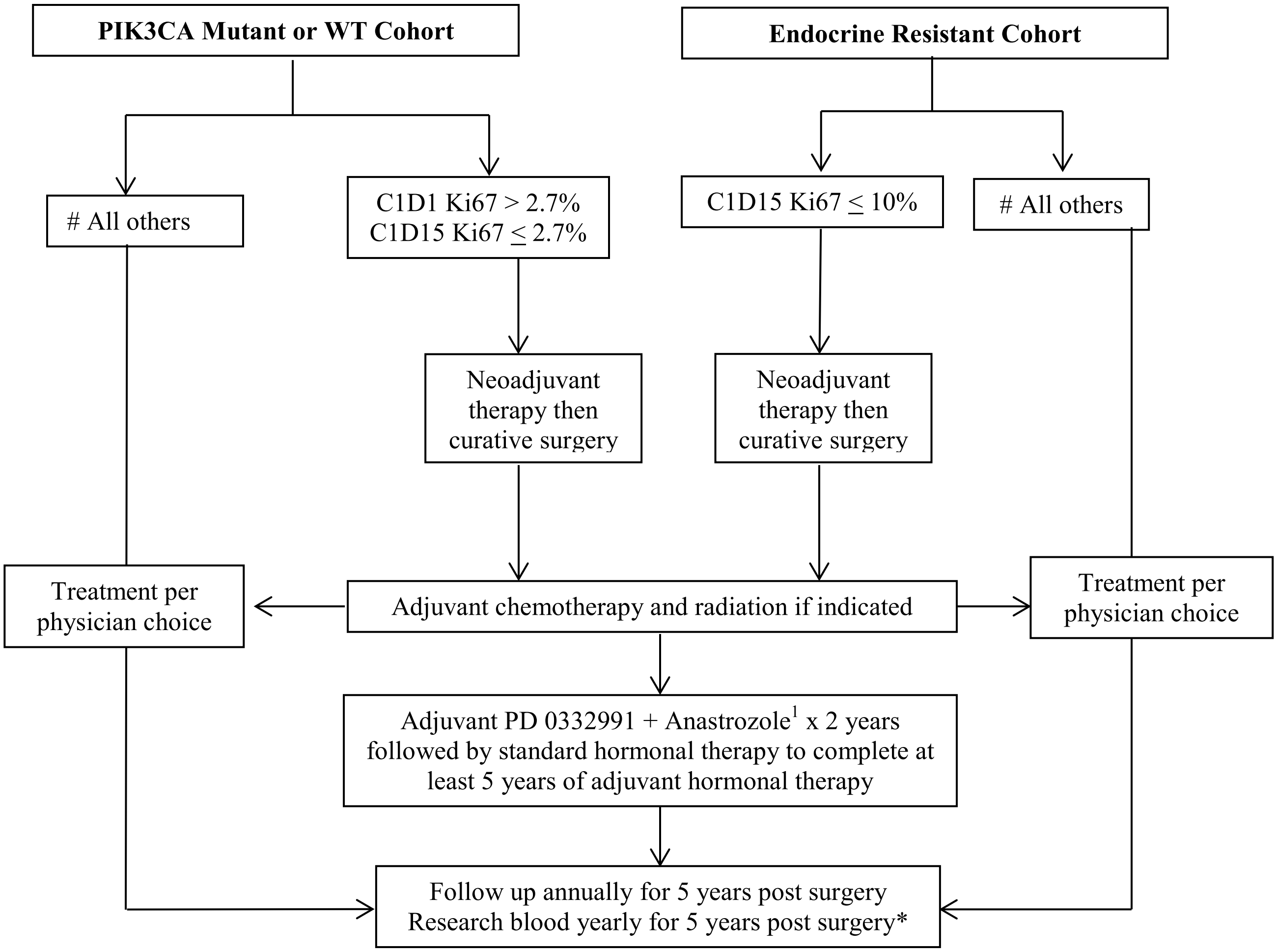

Except those who refuse for followup or did not undergo surgery

* Follow-up for patients who do not receive adjuvant PD 0332991 will take place annually for 5 years post-surgery or until recurrence (whichever comes first). Follow-up for patients who do receive adjuvant PD 0332991 will be tied to treatment, not to date of surgery. These patients will be followed for a total of 5 years (inclusive of the up to 2 years of adjuvant PD 0332991 treatment), with follow-up after discontinuation of PD 0332991 taking place annually after the last dose or until recurrence (whichever comes first). Patients who discontinue adjuvant PD 0332991 early (do not complete the full 23 cycles of treatment) will be followed annually following the last dose of PD 0332991 to get as close as possible to 5 years of post-surgery follow-up or until recurrence (whichever comes first).

[1] Endocrine therapy that is not Anastrozole can be used if patient cannot tolerate Anastrozole (See Section 5.3)

Glossary of Abbreviations

AE	Adverse event
ALT (SGPT)	Alanine transaminase (serum glutamate pyruvic transaminase)
ANC	Absolute neutrophil count
AST (SGOT)	Aspartate transaminase (serum glutamic oxaloacetic transaminase)
AUC	Area under the curve
BCS	Breast conserving surgery
B-HCG	Beta human chorionic gonadotropin
BUN	Blood urea nitrogen
CBC	Complete blood count
Cdk	Cyclin dependent kinase
CFR	Code of Federal Regulations
CNS	Central nervous system
CR	Complete response
CRF	Case report form
CT	Computed tomography
CTCAE	Common Terminology Criteria for Adverse Events
CTEP	Cancer Therapy Evaluation Program
DCIS	Ductal carcinoma in situ
DNA	deoxyribonucleic acid
DOB	Date of birth
DSM	Data and Safety Monitoring
DSMC	Data Safety Monitoring Committee
ECG (or EKG)	Electrocardiogram
ECOG	Eastern Cooperative Oncology Group
EDTA	ethylenediaminetetraacetic acid
ER	Estrogen receptor
FDA	Food and Drug Administration
FFPE	Formalin fixed paraffin embedded
FISH	fluorescent in situ hybridization
FSH	Follicle-stimulating hormone
FWA	Federal wide assurance
GCP	Good Clinical Practice
GnRH	Gonadotropin-releasing hormone
HER2	Human epidermal growth factor receptor 2
HHS	Department of Health and Human Services'
HIV	Human Immunodeficiency Virus
HRPO	Human Research Protection Office (IRB)
IHC	Immunohistochemistry
IND	Investigational New Drug

IRB	Institutional Review Board
IV	Intravenous
LD	Longest diameter
LDH	Lactate dehydrogenase
LH	Luteinizing hormone
LHRH	Luteinizing hormone-releasing hormone
MRI	Magnetic resonance imaging
MTD	Maximum tolerated dose
NC	No change
NCCN	National Cancer Center Network
NCI	National Cancer Institute
OHRP	Office of Human Research Protections
OS	Overall survival
pCR	Pathologic complete response
PD	Progressive disease
PEPI	Preoperative Endocrine Prognostic Index
PI	Principal investigator
PK	Pharmacokinetic
PO	Per os (by mouth)
PR	Partial response
QASMC	Quality Assurance and Safety Monitoring Committee
QD	Quaque die (one a day)
RECIST	Response Evaluation Criteria in Solid Tumors (Committee)
RFS	Relapse-free survival
RR	Response rate
SAE	Serious adverse event
SCC	Siteman Cancer Center
TSH	Thyroid stimulating hormone
TTP	Time to progression
UPN	Unique patient number
US	Ultrasound
WBC	White blood cell (count)
WHO	World Health Organization
WUSM	Washington University School of Medicine

Table of Contents

SCHEMA 4
POSTOPERATIVE THERAPY AND FOLLOW-UP SCHEMA 5
1.0 BACKGROUND AND RATIONALE 10
1.1 ER Positive and HER2 Negative Breast Cancer and Study Overview 10
1.2 Neoadjuvant Endocrine Therapy in ER+ Breast Cancer 11
1.3 Cyclin D/Cdk4/6 Pathway in ER+ Breast Cancer 13
1.4 PD 0332991 15
1.5 Anastrozole 19
1.6 Goserelin 22
1.7 Rationale to Investigate PD 0332991 in Combination with Anastrozole 23
1.8 Rationale for Adding Adjuvant PD 0332991 in Combination with Anastrozole in Patients who Derived benefit from the Addition of PD 0332991 during Neoadjuvant Therapy (Amendment #7) 26
1.9 Correlative Studies Background 27
2.0 OBJECTIVES 30
2.1 Primary Objective 32
2.2 Secondary Objectives 32
2.3 Exploratory Objectives 33
3.0 PATIENT SELECTION 34
3.1 Pre-registration Eligibility Criteria for the PIK3CA Mutant Cohort 34
3.2 Registration Eligibility Criteria for the PIK3CA Mutant Cohort 36
3.3 Eligibility Criteria for the PIK3CA Wild Type Cohort 37
3.4 Eligibility Criteria for the Endocrine Resistant Cohort 40
3.5 Eligibility Criteria for the Adjuvant Cohort 42
3.6 Inclusion of Women and Minorities 45
4.0 PRE-REGISTRATION AND REGISTRATION PROCEDURES 45
4.1 Pre-Registration 45
4.2 Registration 46
5.0 TREATMENT PLAN 48
5.1 Neoadjuvant Treatment 48
5.2 Surgery 49
5.3 Post Surgery Therapy 49
5.4 General Concomitant Medication and Supportive Care Guidelines 50
5.5 Women of Childbearing Potential 50
5.6 Duration of Therapy 50
5.7 Treatment/Follow-up Decision Tree 51
5.8 Duration of Follow-up 53
6.0 DOSE DELAYS/DOSE MODIFICATIONS 53
6.1 Dose Modifications for Anastrozole 53
6.2 Dose Modifications for PD 0332991 54
6.3 Re-Treatment Criteria for Neoadjuvant Cycles 1-4 and Adjuvant Therapy 55
6.4 Re-Treatment Criteria for Cycle 5 56
7.0 REGULATORY AND REPORTING REQUIREMENTS 56
7.2 Reporting to the Human Research Protection Office (HRPO) at Washington University 58

7.3 Reporting to the Quality Assurance and Safety Monitoring Committee (QASMC) at Washington University 59
7.4 Reporting Requirements for Secondary Sites 59
7.5 Reporting to Secondary Sites 59
7.6 Reporting to the FDA 60
7.7 Reporting to Pfizer 60
7.8 Timeframe for Reporting Required Events 61
8.0 PHARMACEUTICAL INFORMATION 61
8.1 Study Agent (PD 0332991) 61
8.2 Anastrozole 67
8.3 Goserelin 68
9.0 CORRELATIVE STUDIES 69
9.1 Sample Collection, Processing, and Shipment 69
9.2 Real Time Integral Biomarker Studies 74
9.3 Tumor Ki67 Assessment on Cycle 1 Day 1 (Pre-PD 0332991) and Cycle 1 Day 15 75
9.4 Laboratory Correlative Studies 76
10.0 STUDY CALENDARS 79
10.1 Pre-Registration and Cycle 0 Calendar 79
10.2 Neoadjuvant Study Treatment Calendar (ALL Cohorts) 80
10.3 Post Surgery Treatment Calendar 81
11.0 DATA SUBMISSION SCHEDULE 82
12.0 MEASUREMENT OF EFFECT 83
12.1 Neoadjuvant Treatment 83
12.2 Treatment Resistance 83
12.3 Surgery 85
12.4 Post-surgery 85
13.0 DATA AND SAFETY MONITORING 85
14.0 AUDITING 86
15.0 STATISTICAL CONSIDERATIONS 87
15.1 Purpose 87
15.2 Primary Endpoint 87
15.3 Trial Design 87
15.4 Sample Size and Trial Duration 89
15.5 Data Analysis 89
15.6 Correlative Studies 90
16.0 MULTICENTER REGULATORY REQUIREMENTS 91
17.0 REFERENCES 93
APPENDIX A: ECOG Performance Status Scale 99
APPENDIX B: Neoadjuvant PD 0332991: Registration Worksheet 100
APPENDIX C: Adjuvant PD 0332991 Registration Worksheet 103
APPENDIX D: Medication Diary – PD 0332991 106
APPENDIX E: Medication Diary – Endocrine Therapy 107
APPENDIX F: Pfizer Reportable Event Cover Sheet 109
APPENDIX H: Strong CYP3A4 Inhibitors or Inducers 111

1.0 BACKGROUND AND RATIONALE

1.1 ER Positive and HER2 Negative Breast Cancer and Study Overview

Estrogen receptor positive (ER+) and Human Epidermal Growth Factor Receptor 2 negative (HER2-) breast cancer represents approximately 70% of breast cancer cases[1]. Although most patients are diagnosed at an early stage and treated with curative intent, there is a persistent risk of relapse over decades[2]. As a result, approximately one-third of patients diagnosed with early stage ER+ breast cancer eventually experience disease recurrence, indicating the failure of current treatments regimens to completely eradicate cancer cells from these patients.

Conventional chemotherapy has been extensively examined in this setting but the impact of these agents is modest in addition to resulting in significant toxicity with little benefit for most patients. In the recent Early Breast Cancer Trialists' Collaborative Group (EBCTCG) overview analysis of randomized trials, the 5-year gain in reducing recurrence is about twice as great for ER poor disease as for ER+ disease[2]. Consistent with this finding is the low rate of pathologic complete response (pCR) for ER+ disease in response to neoadjuvant chemotherapy [3-9]. The pCR rate in the ER+ HER2- subgroup is particularly low, ranging 1.8% to 6%, compared to the over 20% pCR rate in the ER- or HER+ populations, following treatment with neoadjuvant anthracycline/taxane-containing regimens [3, 4, 6].

Endocrine therapy is the most effective systemic adjuvant treatment for ER+ HER2- breast cancer; however a protracted course of therapy is necessary to prevent recurrence, resulting in toxicity and economic burden. Unfortunately, many patients still suffer disease relapse despite long-term endocrine therapy. The therapeutic effects of endocrine treatments are thought to depend on an inhibitory or "cytostatic" effect on the tumor cell cycle [10, 11]. Unlike cytotoxic chemotherapy, it has never been clearly demonstrated that endocrine interventions promote cell death through apoptosis, perhaps explaining why maintenance therapy is necessary and there is a very high frequency of delayed recurrence [12]. In three relatively large studies with aromatase inhibitors, the pCR rate was no more than 1% [13-15].

Recent studies indicated that cyclin D is required for tumor maintenance in breast cancer [16], and inhibition of Cdk 4/6 was able to induce senescence, a permanent form of cell cycle arrest, in preclinical studies [16-20]. The high frequency of cyclin D/Cdk4/6 pathway alteration as a result of cyclin D amplification or loss of negative regulators in ER+ HER2- breast cancer [21] provides a strong scientific rationale for the investigation of Cdk4/6 inhibitors in this subtype. This is supported by the clinical efficacy observed in the randomized phase II trial of the Cdk4/6 inhibitor PD 0332991 in combination with letrozole versus letrozole alone as first line therapy for metastatic ER+ HER2- breast cancer, which demonstrated that the combination therapy was associated with a significant improvement in progression free survival (PFS) from 7.5 to 26.1 months (HR

0.37, 95% CI 0.21 – 0.63, p<0.001) [22]. This has led to the FDA fasttrack listing of this agent to accelerate the review and approval process.

The goal of this neoadjuvant trial is to investigate whether the combination of the aromatase inhibitor anastrozole and the Cdk4/6 inhibitor PD 0332991 induces cell cycle arrest more effectively than anastrozole alone and whether there is induction of senescence with short-term neoadjuvant treatment. In addition, serial tumor samples are collected for investigation of predictive and pharmacodynamics markers of response to treatment. The results of this trial will provide essential efficacy and tolerability information of this combination as well as biomarkers for patient selection for future adjuvant clinical trials.

1.2 Neoadjuvant Endocrine Therapy in ER+ Breast Cancer

1.2.1 Neoadjuvant Aromatase Inhibitor in Postmenopausal Women

Neoadjuvant endocrine therapy with an aromatase inhibitor is now considered a standard treatment option in postmenopausal women with estrogen dependent tumors that are 2 cm or greater (NCCN Guidelines http://www.nccn.org). The primary clinical goal in this setting is reduction in the size of the tumor to allow breast conservation [23]. Preoperative therapy has several additional advantages. From the standpoint of clinical practice, tumor response to neoadjuvant endocrine therapy provides an *in vivo* assessment of its endocrine sensitivity and predicts long term outcome. Most recently a prognostic model has been developed that integrates post-neoadjuvant endocrine therapy pathological stage, ER status and Ki67 level (a marker of cell proliferation) into a Preoperative Endocrine Prognostic Index (PEPI) [11, 24]. In addition, the level of Ki67 following short-term (2-4 weeks) of neoadjuvant endocrine therapy and degree of Ki67 suppression compared to baseline correlated with relapse and survival [12, 25, 26]. From a research standpoint, the neoadjuvant setting provides a fertile environment for using biological endpoints such as Ki67 based biomarkers to efficiently test new therapeutic strategies and to study mechanisms of endocrine therapy resistance [27].

P024 Trial

The P024 trial compared letrozole to tamoxifen in postmenopausal ER+ breast cancer. Letrozole proved superior to tamoxifen in the primary endpoints of clinical response rate, radiographic response rate, and the incidence of breast conservation [14]. Despite a response rate of 60% to letrozole in the 124 confirmed ER+ patients, only one patient achieved a pathologic complete response (pCR)[14]. While the low pCR rate can be an argument against neoadjuvant endocrine therapy, in fact a low rate of pCR is also seen with neoadjuvant chemotherapy regimens in ER+ disease [6]. Thus, ER+ and HER2- breast cancer must be relatively resistant to cancer therapy induced cell death, regardless of the treatment modality.

In the P024 study, assessment of the proliferative marker Ki67 was performed at baseline and at the time of definitive surgery. Interestingly pretreatment Ki67 score did not correlate with relapse free survival in the P024 study. However, subsequent analyses showed that a model (PEPI score) incorporating post treatment Ki67 score, ER status and pathologic stage predicted relapse free survival and breast cancer specific survival [11].

IMPACT Trial

The IMPACT (Immediate Preoperative Anastrozole, Tamoxifen or Combined with Tamoxifen) trial randomized 330 postmenopausal patients to anastrozole, tamoxifen or the combination for 12 weeks prior to definitive surgery. The overall clinical response rate was not significantly different between the three arms; however, significantly more patients classified as requiring mastectomy were eligible for Breast Conservation Surgery (BCS) on the anastrozole treatment arm. Analysis of tumor cell proliferation after 2 weeks of therapy showed significantly lower Ki67 values with anastrozole alone than with either tamoxifen or the combination[25, 26]. Consistent with results obtained in the P024 study, post treatment Ki67 score was a more powerful predictor of recurrence free survival than baseline Ki67 [25].

Surprisingly, neither tamoxifen nor anastrozole treatment in the IMPACT trial activated classical apoptotic cell death in breast tumors. In fact, anastrozole treatment resulted in a significant *decrease* in TUNEL staining in ER+ breast tumors [26]. This conclusion underscores the postulate that ER+ disease tends to be quite resistant to stimuli that can cause apoptosis in normal hormone regulated tissues. An important implication of these studies is that endocrine therapy primarily controls disease through cytostasis, but may not kill disseminated tumor cells. Consequently, when patients stop adjuvant endocrine therapy the relapse rate increases.

ACOSOG Z1031

The ACOSOG Z1031 trial is a randomized phase II study of postmenopausal women with clinical stage II or III ER positive breast cancer to receive anastrozole, exemestane, or letrozole 16-18 weeks prior to surgery. A total of 374 patients were enrolled between January 2006 and January 2009. Clinical response rates were similar among the three treatment arms: 69.1% (95% CI: 60.1-77.1%) for anastrozole, 62.9% (95% CI: 53.8-71.4%) for exemestane and 74.8% (95% CI: 66.3-82.15%) for letrozole. In addition, changes in Ki67 after treatment showed no difference between treatment arms (p=0.45). Geometric mean percentage change in Ki67 was 78% with anastrozole, 81.2% with exemestane and 87.1% with letrozole [28]. In this trial, the pCR rate was approximately 1% (data from Dr. Matthew Ellis).

1.2.2 Neoadjuvant Aromatase Inhibitor in Combination with Ovarian Suppression in Premenopausal Women

Ovarian suppression with GnRH agonist such as goserelin is effective in preventing relapse in premenopausal women with early stage ER+ breast cancer[29]. Goserelin in combination with tamoxifen is more efficacious than tamoxifen alone in TTP, RR and OS in premenopausal women with advanced disease[30]. Goserelin in combination with letrozole in premenopausal women has similar efficacy as compared to letrozole alone in postmenopausal women as the first line hormonal therapy in the metastatic setting[31]. Since aromatase inhibitors are more effective than tamoxifen as adjuvant hormonal therapy for postmenopausal women, there has been considerable interest in testing the combination of goserelin and an aromatase inhibitor in the early stage setting for premenopausal women with ER+ breast cancer.

The comparison of goserelin in combination with either anastrozole or tamoxifen as neoadjuvant therapy for premenopausal women with ER+ HER2- operable breast cancer from the randomized, double-blind, multicenter phase III study of tamoxifen or anastrozole in combination with goserelin (STAGE) was recently reported in abstract forms[32, 33]. Anastrozole and goserelin combination was found to be more effective than the tamoxifen combination in inducing higher response rate by caliper (70.4% vs 50.0%, p=0.004) as well as by ultrasound (58.2% vs 42.4%, p=0.027) and MRI (64.3% vs 37.4%, p<0.001)[32]. A greater reduction in Ki67 was also observed after 24 weeks of anastrozole plus goserelin (n=96, 21.9% to 2.9%) when compared to that of tamoxifen plus goserelin (n=96, 21.6% to 8.0%)[33]. In the same study, the levels of E1 and E2 reached steady state level at 1 month following goserelin. Therefore, we have chosen the combination of goserelin and anastrozole for this study in those who are premenopausal at study entry.

1.3 Cyclin D/Cdk4/6 Pathway in ER+ Breast Cancer

1.3.1 Interaction of Estrogens and Cyclin-Dependent Kinases in Breast Cancer Cells

Studies of ER-positive breast cancer cell lines indicate that estrogens[34] and antiestrogens[35] act on sensitive populations of cells in early to mid-G_1 phase. G_1/S transition is under the control of cyclin-dependent kinases (Cdks) activated by specific complex formation with regulatory cyclins. Cdk4 and Cdk6 are activated by binding to D-type cyclins and act early in G_1 phase[36-39]. A primary target of Cdk action in G_1 phase is the retinoblastoma susceptibility gene product (pRb), which mediates G_1 arrest through sequestration of transcriptional factors of the E2F-DP family. Phosphorylation of pRb and other members of the pocket protein family (p107 and p130) by active cyclin-Cdk complexes leads to release of E2F and DP transcription factors and transcription of requisite genes for S-phase entry[39]. D-type cyclins play an essential role in recognition of extracellular growth stimuli and initiation of G_1 transit[40, 41], and several lines of evidence have linked estrogen regulation of cellular proliferation to cyclin D1 expression. Estrogen-induced proliferation of normal uterine and breast

epithelium in vivo is associated with increased expression of cyclin D1 mRNA and protein[42-45]. Expression of cyclin D1 in breast tumor isolates correlates with ER-positive status[46-48]. MCF-7 breast cancer cells treated with estrogen exhibit increased expression of cyclin D1 mRNA and protein, formation of active cyclin D1-Cdk4 complexes, and phosphorylation of pRb leading to G_1/S transition[49-52]. Estrogen-induced S-phase entry in these cells is inhibited by microinjection of antibodies to cyclin D1[53]. Antiestrogen-induced growth arrest of ER-positive breast cancer cells is associated with decreased cyclin D1 expression[54]. Collectively, these studies are consistent with a model of estrogen action in which receptor activation induces increased cyclin D1 expression, Cdk4 activation, and cell cycle progression. An upstream role for cyclin D1 has been suggested by recent reports describing direct physical interactions between cyclin D1 and the ER, leading to recruitment of steroid receptor coactivators and activation of ER-dependent transcription. This occurs in the absence of hormone and is independent of D cyclin association with Cdk4 [55-58].

Constraint upon Cdk activity and G_1 progression is provided by the universal Cdk inhibitors of the Cip-Kip family, including $p21_{Cip1}$ and $p27_{Kip1}$, and the specific Cdk4 and Cdk6 inhibitors of the INK4 family, typified by $p16_{INK4a}$[41, 59-62]. The $p16_{INK4a}$ gene product inhibits formation of active D cyclin-Cdk complexes through specific binding interactions with Cdk4 or Cdk6 that prevent D cyclin-Cdk association[63-65]. Overexpression of $p16_{INK4a}$ in cells with functional pRb results in inhibition of both Cdk4-and Cdk6-associated kinase activity and pRb phosphorylation, with subsequent cell cycle arrest[63, 64]. In addition, inhibition of D cyclin-Cdk4 complex formation by $p16_{INK4a}$ prevents sequestration of $p21_{Cip1}$ and $p27_{Kip1}$ by these complexes in early G_1, leading to suppression of cyclin E-Cdk2 activity[66-68]. Adenoviral transduction of $p16_{INK4a}$ into MCF-7 cells leads to G_1 arrest associated with inhibited Cdk activity[69, 70]. Cell cycle progression induced by estradiol requires action of the steroid through mid-G_1, well beyond the point of cyclin D1-Cdk4 activation[50]. Functional association of cyclin D1-Cdk4 is required for estrogen-induced Cdk2 activation and G_1/S transition and estrogen regulates expression of $p21_{Cip1}$, $p27_{Kip1}$, and Cdc25A independent of D cyclin-Cdk4 function[71].

1.3.2 Deregulation of Cell Cycle Related Genes and Proteins in Breast Cancer

Cell cycle related genes and proteins are frequently deregulated in breast cancer. Approximately 15-20% of human breast cancers exhibit amplification of D1 (CCND1) gene[72-74], while the majority of human mammary carcinomas overexpress CCND1 protein[75-77]. Overexpression of CCND1 is seen early in breast cancer, and it is maintained at all stages of breast cancer progression, including metastatic lesions[75, 78]. There is a mounting body of evidence linking a specific CCND1 polymorphism (G/A870) to increased risk of cancer and outcome in a variety of tumor types including breast cancer. This polymorphism results in a splice variant, altered protein structure and enhanced oncogenic

activity in experimental models[79]. The continued presence of Cdk4-associated kinase activity is actually required to maintain breast tumorigenesis[80]. Direct analyses of primary tumors have revealed loss of Rb expression in 20-35% of tumors, and loss of heterozygosity or other alterations of the Rb locus in 7–37% of tumors[81-84]. In preclinical models, Rb depletion appears to be associated with resistance to antiestrogen therapy[85].

Finally, virtually all ER-positive cell lines harbor loss of 16_{ink4a}[86, 87], and low expression of Cdk inhibitors p21 and p27 and high expression level of cyclin E and D1 have all been associated with resistance to anti-estrogen therapy.

Data from the Cancer Genome Atlas project confirmed that ER+ breast cancer is enriched for CCND1 (Cyclin D1) amplification (luminal B: 58%; luminal A: 29%), gain of CDK4 (25% in luminal B versus 14% in luminal A), and loss of negative regulators including CDKN2A (p16) and CDKN2C (p18) [21]. In contrast to basal-like breast cancers, Rb is intact in most luminal breast cancer. Since a functional Rb is a pre-requisite for the efficacy of CDK4/6 inhibitors, luminal B breast cancers are ideal candidate for these agents and early success has been observed in clinical trial of CDK4/6 inhibitors.

1.4 PD 0332991

1.4.1 Mechanism of Action

PD 0332991 is a highly selective inhibitor of Cdk4/cyclinD_1 kinase activity. PD 0332991 has selectivity for Cdk4/6, with little or no activity against a large panel of 34 other protein kinases including other Cdks and a wide variety of tyrosine and serine/threonine kinases. Cdk6, another enzyme that also complexes with cyclin-D subunits, is also commonly expressed in mammalian cells and tumors. Cdk6 is highly homologous to Cdk4 and can perform the same function by phosphorylating Rb, thus potentially creating a redundant mechanism to promote cell cycle progression. Consequently, inhibition of both enzymes is necessary to ensure complete suppression of Rb phosphorylation and the greatest possible spectrum of antitumor activity. Results indicate that PD 0332991 inhibits Cdk6 with equivalent potency to Cdk4.

1.4.2 Nonclinical Studies

In vitro single-agent activity of PD 0332991

The only known natural substrate for Cdk4/cyclinD1 is the retinoblastoma gene product (Rb). Specific Cdk4 phosphorylation sites on Rb include serine-780 and serine-795. Therefore, the phosphorylation status of Rb at these specific sites in treated tumors can serve as an appropriate biomarker for target modulation by PD 0332991. The IC_{50} for reduction of Rb phosphorylation at serine-780 in the MDA-MB-435 breast carcinoma cell line was 0.066 μM. PD 0332991 was equally effective at reducing Rb phosphorylation at serine-795 in this tumor cell line with

an IC_{50} of 0.063 μM. Similar effects on serine-780 and serine-795 Phosphorylation were obtained in the Colo-205 colon carcinoma cell line.

PD 0332991 inhibits cellular proliferation and prevents cellular DNA synthesis by preventing cells from entering S phase of the cell cycle. PD 0332991 inhibited thymidine incorporation into the DNA of a panel of Rb-positive human breast, colon, and lung carcinomas, with IC_{50} values ranging from 0.040 to 0.17 μM. PD 0332991 was also effective in preventing cell cycle progression in human leukemias and in non-transformed human epithelial cells and fibroblasts and was equally effective in suppressing cell division in human tumor cell lines. A selective Cdk4/cyclin D inhibitor should cause a specific accumulation of cells in G_1, but have no effect on other phases of the cell cycle, in which cells should continue to progress and eventually decline in number. MDA-MB-453 breast carcinoma cells that were exposed to various concentrations of PD 0332991 for 24 hours show a significant increase in the percentage of cells in G_1 in the presence of as little as 0.04 μM PD 0332991 with a concomitant decline in other phases of the cell cycle.

Finally, to provide further evidence of the selectivity of PD 0332991, the compound was tested against Rb-negative tumor cells, which should not be sensitive to a specific Cdk4 inhibitor. PD 0332991 was tested against the MDA-MB-468 human breast carcinoma and the H2009 human non-small cell lung carcinoma, both of which have deleted Rb. The compound had no anti-proliferative activity on these cells when assayed at 3 μM (highest concentration tested), which is 1 to 2 orders of magnitude higher than the concentration necessary to inhibit Rb-positive tumor cells.

In Vivo Activity Studies

PD 0332991-0002 (hydrochloride salt) was used in all in vivo tumor models. Additionally, PD 0332991-0054 (isethionate salt) was used in the MDA-MB-435 breast carcinoma model, and had comparable efficacy to the hydrochloride salt.

The MTD in SCID mice was 150 mg/kg/day when administered orally, once a day, for 14 days. The MTD was defined as the highest dose that was nonlethal ($<LD_{10}$). At the MTD on this regimen, PD 0332991 has significant antitumor efficacy against multiple human tumor xenograft models. The Colo-205 model is exquisitely sensitive to PD 0332991. At doses as low as 12.5 mg/kg, a 13-day growth delay was obtained, indicating a 90% inhibition of tumor growth rate. PD 0332991 was inactive against the H23 lung and the SW-620 colon carcinomas. The lack of response may be associated with the presence of oncogenic K-ras mutations in SW-620 and H23; none of the xenografts sensitive to PD 0332991 had such mutations.

Further evidence that the anti-tumor activity observed in Rb-positive tumors is due to inhibition of Cdk4/Cdk6 protein kinase activity was obtained by testing PD 0332991 in the MDA-MB-468 breast carcinoma and the DU-145 prostate tumor

models. These are Rb negative tumors; neither of which responded to this compound. The lack of efficacy in Rb-negative tumors is consistent with the lack of anti-proliferative activity observed in vitro. Taken together, these results support the proposed mechanism of PD 0332991 (inhibition of Cdk4/6-mediated Rb phosphorylation) and the specificity of the compound demonstrated in enzyme activity tests.

Further studies investigated whether continuous daily dosing of PD 0332991 was needed for optimal efficacy. Four dosing schedules were employed against the MDA-MB-435 breast carcinoma model over 14 days of treatment, including continuous daily, every other day, every third day, and 3 courses of 3 days dosing followed by 4-day drug holidays. The design of this experiment was such that the total compound administered over the 2-week period was identical for each treatment schedule. The results show that a similar degree of efficacy was attained with all schedules, implying that an intermittent regimen is feasible without compromising activity. Similar experiments were conducted against the Colo-205 colon carcinoma model. Again, intermittent schedules were as efficacious as daily dosing, with tumor regressions occurring during all dosing regimens.

During the 14-day treatment period employed for most of the efficacy experiments, no cures were documented, and the tumors grew back after therapy. It is possible that a tumor variant had selectively grown back and acquired resistance to the compound. To address this possibility, Colo-205 colon tumors that had initially significantly regressed in response to treatment with PD 0332991 were harvested and reimplanted into naive mice. After the tumors grew to 100 to 150 mg, these tumor-bearing mice were treated with PD 0332991 with a dose and dosing schedule identical to the original experiment. The tumors responded with equal sensitivity to the drug and fully regressed, indicating that no resistance had developed during the initial treatment. A similar result was observed with retreated MDAMB-435 tumors.

<u>Pharmacokinetics</u>

The single-dose pharmacokinetics of PD 0332991 following IV or PO routes of administration were investigated in Sprague-Dawley rats and Beagle dogs (toxicology species), and in cynomolgus monkeys. PD 0332991 was administered intravenously to determine elimination kinetics and absolute bioavailability from the PO route. Following IV administration, mean plasma clearance values of PD 0332991 in all species were low to moderate and were all lower than the corresponding hepatic blood flow. The mean apparent volumes of distribution at steady state were approximately 10-fold greater than total body water. Mean absolute oral bioavailability of PD 0332991 was moderate in all species tested. In rats on Day 1 of repeat dose studies, mean PD 0332991 C_{max} and AUC values increased in a dose-related manner up to 300 mg/kg. In dogs, mean PD 0332991 C_{max} and AUC values increased in a dose-related manner up to 20 mg/kg on Day 1, and did not increase between 20 and 40 mg/kg. Mean PD 0332991 C_{max} and AUC values in female rats were less than in male rats (up to one ninth and up to

one sixteenth the values for male rats, respectively). There was no observed sex difference in systemic exposure in dogs. Mean PD 0332991 C_{max} and AUC values following 3 weeks of dosing indicate up to 3-fold accumulation upon multiple dosing in both rats and dogs.

1.4.3 Clinical Development of PD 0332991

Currently, fourteen studies evaluating the safety, efficacy, pharmacodynamics and PK of PD 0332991 as single agent or in combination have started. Results from earlier phase I trials have been reported and are discussed below.

1.4.4 Clinical Pharmacokinetics

To date pharmacokinetic data is available from four studies (see Section 8.1.3). The exposure ($AUC_{(0-10)}$ and C_{max}) increased in a dose-proportional manner over the dose range of 25-225 mg QD following PD 0332991 administration on Days 1 and 8 of Cycle 1. At a steady state (Day 14 or Day 21), PD 0332991 was absorbed with a median T_{max} of ~4 hours. PD 0332991 extensively penetrates into peripheral tissues, and was eliminated slowly; the mean elimination half-life (t1/2) was 26.5 hours.

The preliminary results from the recently performed food-effect study ("A5481021, a Phase 1, open-label 4 sequence 4 period crossover study of palbociclib (PD-0332991) in healthy volunteers to estimate the effect of food on the bioavailability of palbociclib") has provided evidence that when a single 125 mg dose of palbociclib was administered under fed conditions (including high fat or low fat meal given together with palbociclib, or moderate fat meal given 1 hour before and 2 hours after palbociclib) as a freebase formulation the palbociclib exposure levels were more uniform across the population than when taken in the fasting condition.

1.4.5 Clinical Toxicology

Since protocols A5481001 and A5481002 both tested PD 0332991 as a single agent in advanced cancers, the relevant safety data have been combined (see Section 8.1.11 Table 16). The most frequently reported AEs were predominantly considered treatment-related. These treatment-related events included fatigue, neutropenia, diarrhea, nausea, anemia, and thrombocytopenia. The most common Grade 4 adverse events were neutropenia and thrombocytopenia

<u>QT Interval Effects</u>
Data from non-clinical (in vitro and in vivo) studies indicated that PD 0332991 has the potential to delay cardiac repolarization as measured by prolongation of the QT interval on the ECG. Prolongation of QTcF (maximum increase of < 30 msec from baseline) was observed in a majority of patients in two Phase I trials with PD 0332991 as a single agent. No patient had a maximum QTcF value of ≥

500 msec during treatment. Notably, one female patient receiving PD 0332991 at 75 mg QD on Schedule 3/1 had a maximum QTcF increase of 67 msec from baseline to Cycle 1. Additionally, QTcF increases ranging from 39 to 51 msec compared to baseline persisted throughout her ECG collection period of 5 subsequent cycles. No significant changes in blood pressure, pulse rate and body weight have been observed in the two completed Phase 1 clinical studies in advanced cancers.

The patients enrolled in clinical studies should be closely monitored for potential cardiovascular symptoms. Appropriate monitoring should include clinical examinations, vital signs, routine ECGs, and AEs monitoring.

1.4.6 Developmental/Reproductive Toxicity

Fertility and teratology studies with PD 0332991 have not been conducted. Women of childbearing potential must have a negative pregnancy test prior to treatment with PD 0332991. Female patients must be surgically sterile or be postmenopausal, or must agree to use effective contraceptive during the period of the trial and for at least 90 days after completion of treatment.

1.5 Anastrozole

1.5.1 Mechanism of Action

Anastrozole is an FDA-approved agent used in the management of hormone receptor positive breast cancers. In postmenopausal women, the principal source of circulating estrogen (primarily estradiol) is conversion of adrenally-generated androstenedione to estrone by aromatase in peripheral tissues, such as adipose tissue, with further conversion of estrone to estradiol. Many breast cancers also contain aromatase; the importance of tumor-generated estrogens is uncertain. Treatment of breast cancer has included efforts to decrease estrogen levels, by ovariectomy premenopausally and by use of anti-estrogens and progestational agents both pre- and post-menopausally; and these interventions lead to decreased tumor mass or delayed progression of tumor growth in some women. Anastrozole is a potent and selective non-steroidal aromatase inhibitor. It significantly lowers serum estradiol concentrations and has no detectable effect on formation of adrenal corticosteroids or aldosterone [AstraZeneca, Package Insert].

1.5.2 Pharmacodynamics/kinetics

Inhibition of aromatase activity is primarily due to anastrozole, the parent drug. Studies with radiolabeled drug have demonstrated that orally administered anastrozole is well absorbed into the systemic circulation with 83 to 85% of the radiolabel recovered in urine and feces. Food does not affect the extent of absorption. Elimination of anastrozole is primarily via hepatic metabolism (approximately 85%) and to a lesser extent, renal excretion (approximately 11%),

and anastrozole has a mean terminal elimination half-life of approximately 50 hours in postmenopausal women. The major circulating metabolite of anastrozole, triazole, lacks pharmacologic activity. The pharmacokinetic parameters are similar in patients and in healthy postmenopausal volunteers. The pharmacokinetics of anastrozole are linear over the dose range of 1 to 20 mg and do not change with repeated dosing. Consistent with the approximately 2-day terminal elimination half-life, plasma concentrations approach steady-state levels at about 7 days of once daily dosing and steady-state levels are approximately three- to four-fold higher than levels observed after a single dose of anastrozole. Anastrozole is 40% bound to plasma proteins in the therapeutic range [AstraZeneca, Package Insert].

Effect on Estradiol: Mean serum concentrations of estradiol were evaluated in multiple daily dosing trials with 0.5, 1, 3, 5, and 10 mg of anastrozole in postmenopausal women with advanced breast cancer. Clinically significant suppression of serum estradiol was seen with all doses. Doses of 1 mg and higher resulted in suppression of mean serum concentrations of estradiol to the lower limit of detection (3.7 pmol/L). The recommended daily dose, anastrozole 1 mg, reduced estradiol by approximately 70% within 24 hours and by approximately 80% after 14 days of daily dosing. Suppression of serum estradiol was maintained for up to 6 days after cessation of daily dosing with anastrozole 1 mg.

Effect on corticosteroids: In multiple daily dosing trials with 3, 5, and 10 mg, the selectivity of anastrozole was assessed by examining effects on corticosteroid synthesis. For all doses, anastrozole did not affect cortisol or aldosterone secretion at baseline or in response to ACTH. No glucocorticoid or mineralocorticoid replacement therapy is necessary with anastrozole.

Other Endocrine Effects: In multiple daily dosing trials with 5 and 10 mg, thyroid stimulating hormone (TSH) was measured; there was no increase in TSH during the administration of anastrozole. Anastrozole does not possess direct progestogenic, androgenic, or estrogenic activity in animals, but does perturb the circulating levels of progesterone, androgens, and estrogens [AstraZeneca, Package Insert].

Studies in postmenopausal women demonstrated that anastrozole is extensively metabolized with about 10% of the dose excreted in the urine as unchanged drug within 72 hours of dosing, and the remainder (about 60% of the dose) is excreted in urine as metabolites. Metabolism of anastrozole occurs by N-dealkylation, hydroxylation and glucuronidation. Three metabolites of anastrozole have been identified in human plasma and urine. The known metabolites are triazole, a glucuronide conjugate of hydroxy-anastrozole, and a glucuronide of anastrozole itself. Several minor (less than 5% of the radioactive dose) metabolites have not been identified. Because renal elimination is not a significant pathway of elimination, total body clearance of anastrozole is unchanged even in severe (creatinine clearance less than 30 mL/min/1.73m2) renal impairment, dosing

adjustment in patients with renal dysfunction is not necessary. Dosage adjustment is also unnecessary in patients with stable hepatic cirrhosis [AstraZeneca, Package Insert].

1.6 Goserelin

1.6.1 Mechanism of Action

Goserelin is a synthetic analog of endogenous gonadotropin-releasing hormone (GnRH) also known as luteinizing hormone releasing hormone (LHRH) agonist. LHRH regulates follicle-stimulating hormone (FSH) and luteinizing hormone synthesis and secretion by the anterior pituitary gland, which in turn stimulates the production of sex hormones estrogen and testosterone by the ovary and testis respectively. In response to LHRH, FSH and LH synthesis initially increases, causing a transient increase in circulating levels of sex hormones. These hormones are however, regulated by feedback loops, so further hormone release is suppressed. Chronic administration of goserelin leads to sustained suppression of pituitary gonadotropins. With continued administration for more than 1-3 weeks, the pituitary gland down-regulates and desensitizes LHRH receptors, reducing FSH and LH secretion. Although the physiologic effects are complicated, the end result of continuous goserelin administration is an effective chemical castration. In women the estradiol levels transiently increases and later falls to postmenopausal levels by three weeks of continuous therapy. Normal pituitary and gonadal functions typically returns within three months of discontinuing goserelin.

1.6.2 Pharmacodynamics/kinetics

In females, a down-regulation of the pituitary gland by chronic exposure to goserelin leads to suppression of gonadotropin secretion, a decrease in serum estradiol to levels consistent with the postmenopausal state, and would be expected to lead to a reduction of ovarian size and function, reduction in the size of the uterus and mammary gland, as well as a regression of sex hormone-responsive tumors, if present. Serum estradiol is suppressed to levels similar to those observed in postmenopausal women within 3 weeks following initial administration; however, after suppression was attained, isolated elevations of estradiol were seen in 10% of the patients enrolled in clinical trials. Serum LH and FSH are suppressed to follicular phase levels within four weeks after initial administration of drug and are usually maintained at that range with continued use of goserelin. In 5% or less of women treated with goserelin, FSH and LH levels may not be suppressed to follicular phase levels on day 28 post treatment with use of a single 3.6 mg depot injection. In certain individuals, suppression of any of these hormones to such levels may not be achieved with goserelin. Estradiol, LH and FSH levels return to pretreatment values within 12 weeks following the last implant administration in all but rare cases [AstraZeneca, Package Insert].

The pharmacokinetics of goserelin have been determined in both male and female healthy volunteers and patients. In these studies, goserelin was administered as a single 250 μg (aqueous solution) dose and as a single or multiple 3.6 mg depot

dose by subcutaneous route. The absorption of radiolabeled drug was rapid, and the peak blood radioactivity levels occurred between 0.5 and 1.0 hour after dosing.

Goserelin is released from the depot at a much slower rate initially for the first 8 days, and then there is more rapid and continuous release for the remainder of the 28-day dosing period. Despite the change in the releasing rate of goserelin, administration of goserelin every 28 days resulted in testosterone levels that were suppressed to and maintained in the range normally seen in surgically castrated men. When goserelin 3.6 mg depot was used for treating male and female patients with normal renal and hepatic function, there was no significant evidence of drug accumulation. However, in clinical trials the minimum serum levels of a few patients were increased. These levels can be attributed to interpatient variation. The apparent volumes of distribution determined after subcutaneous administration of 250 µg aqueous solution of goserelin were 44.1 and 20.3 liters for males and females, respectively. The plasma protein binding of goserelin obtained from one sample was found to be 27.3%. Metabolism of goserelin, by hydrolysis of the C-terminal amino acids, is the major clearance mechanism. The metabolism of goserelin in humans yields a similar but narrow profile of metabolites to that found in other species. All metabolites found in humans have also been found in toxicology species. Clearance of goserelin following subcutaneous administration of the solution formulation of goserelin is very rapid and occurs via a combination of hepatic metabolism and urinary excretion. More than 90% of a subcutaneous radiolabeled solution formulation dose of goserelin is excreted in urine. Approximately 20% of the dose in urine is accounted for by unchanged goserelin. The total body clearance of goserelin (administered subcutaneously as a 3.6 mg depot) was significantly ($p<0.05$) greater (163.9 versus 110.5 L/min) in females compared to males. [AstraZeneca, Package Insert].

1.7 Rationale to Investigate PD 0332991 in Combination with Anastrozole

In preclinical studies, endocrine resistance was associated with persistent cyclin D expression and RB phosphorylation despite efficient blockade of ER and the poor prognosis luminal B breast cancers were associated with a gene expression signature of RB-dysfunction [20]. PD 0332991 was shown to have anti-tumor activity for multiple tumor types including breast cancer in both in vitro and in vivo studies [88, 89]. In preclinical studies, PD 0332991 was particularly effective in inhibiting cell growth of luminal breast cancer subtype [88]. A synergistic anti-tumor effect was also observed when combined with tamoxifen in tamoxifen-sensitive and resistant cell lines [88]. Importantly, treatment with PD 0332991 effectively suppressed proliferation of ER+ breast cancer cell lines resistant to anti-estrogen and led to irreversible cell cycle arrest and features of cellular senescence [20]. These studies provided a preclinical rationale to combine endocrine therapy and a Cdk4/6 inhibitor to improve tumor control for ER+ breast cancer.

The combination of PD 0332991 and endocrine therapy has shown highly promising results in patients with advanced ER+ HER2- breast cancer. In the randomized phase 2 study of letrozole with or without PD 0332991 as first line therapy for metastatic ER+, HER2- breast cancer, the PFS was significantly better in the combination arm (26.1 months vs 7.5 months (HR 0.37, 95% CI 0.21 – 0.63, p<0.001) [22]. The response rate for the combination arm (n = 84) was 31% vs. 26% for the letrozole arm (n = 81) and the clinical benefit rate was 68% vs. 44%, respectively. Toxicities were tolerable. The most commonly reported treatment-related AEs in the combination arm were neutropenia (grade 1/2: 23%; grade 3: 55%, grade 4: 6%), leukopenia (grade 1/2: 29%; grade 3: 17%, grade 4: 0%), anemia (grade 1/2: 24%; grade 3: 5%, grade 4: 1%), and fatigue (grade 1/2: 35%; grade 3: 2%, grade 4: 2%) and nausea (grade 1/2: 23%; grade 3:2%, grade 4: 0%). The promising clinical data led to the FDA fast-track listing of PD 0332991.

In the Ki67 analysis of the serial tumor biopsy samples collected from the first 7 patients enrolled in this trial, comparing data for C1D15 (2 weeks on PD 0332991 and 6 weeks on anastrozole) and C1D1 (4 weeks on single agent anastrozole), the addition of PD 0332991 to anastrozole led to further reduction in Ki67 (Figure 1) in 4 patients. In these responsive tumors, Ki67 was reduced to <1% after 2 weeks of combination therapy, indicating a profound anti-proliferative effect of PD 0332991. In contrast, Ki67 was not suppressed by either anastrozole or the combination in 3 other patients, indicating treatment resistance. Interestingly, one patient (PD001) had a tumor Ki67 >10% on C1D1, but achieved complete cell cycle arrest, Ki67 at 0.5% on C1D15 after combination therapy, indicating that endocrine resistant tumors could be responsive to the combination therapy with PD 0332991.

Based on these preliminary data and the accumulating evidence that PD 0332991 exerts anti-tumor effects through inhibition of cell cycle, inducing senescence, without apoptosis, we have elected to modify the primary endpoint of the study from pathologic complete response at surgery to complete cell cycle arrest on treatment. We propose to investigate whether the combination of PD 0332991 and anastrozole is able to improve the rate of complete cell cycle arrest when compared to the historical control of single agent aromatase inhibitors and also adding an endocrine therapy resistant cohort and the PIK3CA mutant cohort.

Figure 1 Tumor Ki67 response (preliminary data from the first 7 patients enrolled in the current trial).

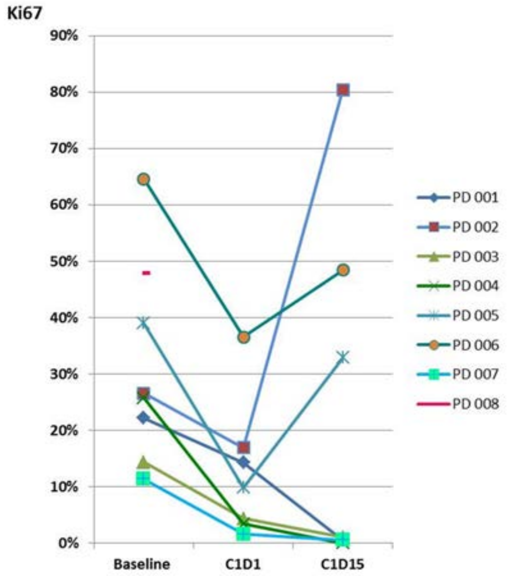

Ongoing Ki67 data from current trial			
ID#	Baseline	C1D1	C1D15
PD 001	22%	14%	0.5%
PD 002	27%	17%	80%
PD 003	14%	4%	1%
PD 004	26%	3%	0%
PD 005	39%	10%	33%
PD 006	65%	37%	48%
PD 007	11%	2%	< 1%

Note that 4 patients had Ki67 <1% on C1D15, indicating profound inhibition of cell proliferation by PD 0332991. The 3 patients with Ki67 >10% on C1D15 were considered resistant to therapy and have been taken off study per protocol.

Baseline (pre-registration): prior to anastrozole
C1D1: post 1 month of single agent anstrozole
C1D15: post 2 weeks after the addition of PD 0332991

Since PD 0332991 is commonly associated with myelosuppression, surgery is scheduled in 3-5 weeks following the last dose of PD 0332991 in this trial. As a result, patients are off PD 0332991 at the time of surgery. Therefore the effect of combination therapy on cell cycle arrest will be evaluated at an earlier time point, Cycle 1 Day 15, when patients are taking both PD 0332991 and anastrozole.

In this trial, we define complete cell cycle arrest as tumor Ki67 $\leq$2.7% since this is the Ki67 cutpoint developed for PEPI score calculation in the PO24 trial [11] and validated in the our Ki67 scoring SOP [90].

We will use data from the ACOSOG Z1031 trial, a large neoadjuvant aromatase inhibitor trial in ER+ HER2- clinical stage II or III breast cancer, as the historical control as it enrolled the same patient population [28]. In the neoadjuvant ACOSOG Z1031 trial, 95 of 215 (44%) patients had Ki67 $\leq$2.7% on 2-4 week tumor biopsy (Personal Communication with Matthew Ellis).

1.7.1 PIK3CA mutation in ER-positive breast cancer and rationale to investigate PD 0332991 in both PIK3CA wild type and mutant populations

PIK3CA, the alpha catalytic subunit of PI3K, is the most common significantly mutated genes identified in luminal breast cancer, occurring at a frequency of 45% and 29% in luminal A and luminal B, respectively [21, 91-93]. Up to 80% of the mutations are restricted to "hotspots" within the helical (HD) and the kinase domains (KD) encoded by exons 9 and 20, respectively [94]. The three hot spot mutations include E542K, E545K and H1047R [94]. Consistent with potentially

important roles of these mutations in the process of tumorigenesis, the majority of these mutations are missense mutations that occur in evolutionarily conserved regions [94]. Many of the *PIK3CA* mutations, including the 3 hot spot mutations, have been shown to increase levels of phosphorylated AKT (pAKT) and induce cellular transformation and invasion *in vitro* and tumor formation *in vivo* when introduced into human mammary epithelial cells [95, 96]. Tumor cells with mutations in PIK3CA have shown to be highly dependent on p110 alpha for cell survival [97].

The relationship between PIK3CA mutation and endocrine therapy responsiveness in ER+ breast cancer was investigated retrospectively in several neoadjuvant endocrine trials [93, 98]. No interaction was between PIK3CA mutation and response to endocrine therapy was identified in these studies [93, 98].

The relationship between PIK3CA mutation and responsiveness of ER+ breast cancers to CDK4/6 inhibitors has not been investigated. In preclinical studies, PD0332991 was found to be effective in a subpopulation of both PIK3CA mutant and wild type cancer [88]. The most promising predictors of response identified in preclinical studies have been the presence of intact Rb or down regulation of p16; however, this has not been confirmed in the recently reported clinical trial of letrozole in combination with PD 0332991 as first line therapy for metastatic ER+ breast cancer [22]. Since PIK3CA mutation is an important driver event in ER+ breast cancer, and direct inhibitors of PI3K, including those that target specifically the alpha catalytic subunit, are being developed to treat tumors with PIK3CA mutation, it is important to investigate the anti-tumor effect and predictors of response to PD 0332991 in PIK3CA wild type and mutant population separately.

The study was designed initially to focus on the population of ER+ breast cancer without PIK3CA hotspot mutations since trials of inhibitors against PI3K pathway, such as the NCI9170, were ongoing the same time for the population with PIK3CA hotspot mutations. As the NCI9170 trial that enrolls patients with PIK3CA mutant tumors is approaching its enrollment target as of November 2013, we plan to open the PIK3CA mutant cohort for this trial, starting in institutions that do not have NCI9170 open.

1.8 Rationale for Adding Adjuvant PD 0332991 in Combination with Anastrozole in Patients who Derived benefit from the Addition of PD 0332991 during Neoadjuvant Therapy (Amendment #7)

Between 4/23/2013 and 4/24/2015, the study enrolled 50 patients with clinical stage II or III ER+ HER2- breast cancer to the PIK3CA mutant and PIK3CA wild type (WT) cohorts. Of these 50 patients, 45 (PIK3CA WT, n=28; PIK3CA mutant, n=15; PIK3CA unknown, n=2) were evaluable for Ki67 analysis at C1D15 after completion of 6 weeks of anastrozole and 2 weeks of PD 0332991 (for the primary endpoint). The data was

presented at 2015 SABCS {Ma, 2015 #3841}. Complete cell cycle arrest (CCCA) was achieved in 39 of the 45 (87%, 90% CI: 75-94%) evaluable patients, 22 of 28 (79%, 90% CI: 62-90%) patients in the PIK3CA WT cohort and all 15 (100%, 90% CI: 82-100%) patients in the PIK3CA mutant cohort. The study met its primary endpoint of achieving a CCCA rate of 66% in C1D15. In addition, 26 patients who did not achieve CCCA with anastrozole alone on C1D1 achieved complete CCCA in C1D15 post 2 weeks of combination therapy. These data justify the evaluation of PD 0332991 in the adjuvant setting. The international PALLAS (PALbociclib CoLlaborative Adjuvant Study) trial is currently enrolling patients with high risk ER+ HER2- breast cancer to receive 2 years of palbociclib (PD 0332991) in the adjuvant setting. However, patients who received neoadjuvant PD 0332991 (palbociclib) are not eligible for the adjuvant PALLAS trial. We therefore propose to include in this trial the option of adjuvant PD 0332991 with anastrozole for 23 cycles after chemotherapy and radiation therapy for those who derived benefit from PD 0332991 in the neoadjuvant setting in this trial. This includes:

- The 26 patients who achieved complete cell cycle arrest only after the addition of PD 0332991 (C1D1 Ki67 >2.7% and C1D15 Ki67 ≤ 2.7%) (in the PIK3CA WT, mutant, or unknown cohorts).
- Patients who have a Ki67 ≤ 10% on C1D15 biopsy in the endocrine resistant cohort (including patients enrolled prior to activation of Amendment #7).

All patients (with the exception of those who withdraw consent for follow-up and those who did not undergo surgery) will be followed for invasive disease recurrence and overall survival for 5 years in the adjuvant setting or until recurrence (whichever comes first). Blood will be drawn for serum, plasma, and circulating cell-free DNA (cfDNA) yearly.

1.9 Correlative Studies Background

1.9.1 Early Tumor Ki67 Assessment during Neoadjuvant Endocrine Therapy to Determine Treatment Response

In this trial, we plan to perform tumor biopsy to assess Ki67 following one month of endocrine therapy (pre-PD 0332991) and 2 weeks on combination of PD 0332991 and anastrozole (Cycle 1 Day 15). This is based on data from previous neoadjuvant endocrine trials which indicated that 2-4 week tumor Ki67 expression on neoadjuvant endocrine therapy is predictive of individual patient outcome long term[11, 25, 26]. In the IMPACT trial, 2-week Ki67 was a significant independent predictor of RFS (HR = 1.95; 95% CI = 1.23–3.07; P = .004) [25]. The 5-year RFS rates were 85%, 75%, and 60% for the lowest, middle, and highest values of 2-week Ki67 expression, respectively[25]. In the P024 trial, while baseline Ki67 was not associated with relapse, post 16-week treatment Ki67 levels had a robust association with RFS (HR = 1.4, CI = 1.2–1.6 per natural log unit increase; $P < .001$), and breast cancer–specific survival (HR = 1.4, CI = 1.1–1.7; $P = .009$) [11].

To further investigate these findings, Ellis et al examined the interaction between

Ki67 levels and a PAM50-based definition of luminal A breast cancer versus luminal B breast cancer. By using ROC methodology, a cut point of Ki67 10% served as the best surrogate for the LumA versus LumB distinction [99]. The 10% Ki67 cut point was then applied to the baseline and early on-treatment data in two data sets (Table 2), Preoperative Letrozole study (POL) [13] and IMPACT trial [25]. At baseline the dichotomized Ki67 definition was not significantly predictive for surgical Ki67 level, PEPI score or RFS in these modest sized sample sets. In contrast, high levels of Ki67 on the one month POL samples predicted a higher level of Ki67 in the surgical samples at four months after treatment initiation (P=.01), a poorer PEPI score (P=0.01), a smaller number of patients in the PEPI-0 group (P=0.08) and worse RFS (P=0.003). IMPACT data confirmed that a 2-week Ki67 >10% predicted higher Ki67 in the surgical specimen (P=0.001), a poorer PEPI score (P=0.001), smaller numbers of patients in the PEPI-0 group (P= 0.004) and worse RFS (P=0.008) (Table 2.3).

Table 2 Early Ki67 Assessments and Outcome in IMPACT and POL Trials

POL 4W Ki67	% PEPI 0	RFS (events)
>10%	1/19 (5%)	5/21 (23%)
≤10%	10/36 (28%)	1/41 (2.4%)
P Value	P=0.08 (Fisher)	P=0.003 (log rank)
IMPACT 2W Ki67	% PEPI 0	RFS (events)
>10%	0/32 (0%)	9/35 (26%)
≤10%	21/101 (21%)	13/118 (11%)
P Value	P=0.004 (Fisher)	P=0.008 (log rank)

In this trial, to avoid futile therapy, we plan to obtain tumor biopsy post 2 weeks of PD 0332991 in combination with anastrozole. Patients whose Day 15 Ki67>10% will be considered resistant to the combination therapy. These patients will be recommended alternative therapies such as immediate surgery or neoadjuvant chemotherapy. The pre-PD 0332991 Ki67 will not be used for clinical decision making since PD 0332991 will be added to the treatment regimen. It is, however, important to obtain the pre-PD 0332991 Ki67 since this allows a comparison between anastrozole alone and the combination of anastrozole and PD 0332991 on the degree of Ki67 suppression.

1.9.2 To Assess Tumor Cell Apoptosis on Biopsies Taken Pre-PD 0332991 and Cycle 1 Day 15

We will investigate whether there is an increase in tumor cell apoptosis with the combination of PD 0332991 and anastrozole (or anastrozole in combination with goserelin). Anastrozole 1mg daily dosing reduced estradiol level by 70% by 24 hours and by approximately 80% after 14 days of daily dosing [AstraZeneca,

Package Insert], therefore, full estrogen deprivation is expected by 28 days of anastrozole administration. In the case of goserelin, serum estradiol is suppressed to levels similar to those observed in postmenopausal women within 3 weeks following administration. In addition, only 5% or less of women treated with goserelin, FSH and LH may not be suppressed to follicular phase levels on day 28 post treatment with a single 3.6mg depot injection [AstraZeneca, Package Insert].

1.9.3 To Assess the Degree of Ki67 Suppression before (Pre-PD 0332991) and after PD 0332991 (Cycle 1 Day 15)

The degree of Ki67 suppression following 2-4 weeks of therapy has been associated with the effectiveness of endocrine agents or the combination of endocrine agent with an mTOR inhibitor. In the IMPACT trial, suppression of Ki67 after 2 and 12 weeks was significantly greater with anastrozole than with tamoxifen (P = 0.004) which mirrored the result based on RFS in the ATAC trial [100]. These data indicate that Ki67 response early on therapy could potentially be employed as a measurement of treatment efficacy.

1.9.4 To Assess Effect of PD 0332991 on Markers of Tumor Cell Senescence

Studies in preclinical models of ER+ breast cancer indicated that PD 0332991 induced stable cell cycle arrest that was fundamentally distinct from those elicited by ER antagonists and was capable of inducing aspects of cellular senescence, as measured by expression of senescence-associated b-galactosidase [20]. In addition, candidate proteins important for senescence including p16, IL6, cyclin D1, activated mTOR (such as pS6), FOXM1 phosphorylation, will be examined [19, 101]. These markers will be assayed on tumors collected at all time points. Since surgery occurs after at least 3 weeks after the last dose of PD 0332991, a comparison of these markers as well as Ki67 level between surgery and C1D15 sample will be informative in determining whether 4 months of combination therapy induced irreversible cell cycle arrest and cells have entered senescence.

1.9.5 Evaluation of Target Inhibition on the Cdk4/6-Rb Pathway

The pharmacodynamic effect of PD 0332991 in combination with anastrozole on the Cdk4/6-Rb pathway activities will be assessed by phosphoroproteomics and immunohistochemistry analysis on serial tumor biopsies. Since PD 0332991 inhibits Cdk4/6 phosphorylation of Rb at Ser $^{801/811}$ and Ser^{780}, the levels of total and phosphorylated RB will be examined at baseline, post 1 cycle of single agent anastrozole, then post 2 weeks of PD 0332991 in combination with anastrozole.

1.9.6 Preoperative Endocrine Prognostic Index (PEPI)

In a multivariable analysis conducted on the P024 trial, three other post-neoadjuvant endocrine therapy tumor factors were determined to have independent prognostic value for relapse and death after relapse in addition to

Ki67 [11]. These included pathological tumor size (T1/2 versus T3/4), pathological node status (positive or negative), the natural logarithm of the Ki67 value and the ER status of the tumor. A prognostic score, the preoperative endocrine prognostic index (PEPI), was developed, which weighs each of these factors according to their associated hazard ratios. PEPI was then validated in an independent data set from the IMPACT trial [11]. No relapses were recorded in either trial in patients with T1, N0 tumors with a PEPI score of 0 (residual tumor with Ki67 index of 2.7% - natural logarithm of 1- or less with maintained ER expression) or in the rare patient with a pCR. PEPI has also recently been validated in the POL Trial (PreOperative Letrozole trial: A multicenter phase II trial of letrozole in postmenopausal women with clinical stage II or III hormone receptor positive breast cancer) [13]. In the combined analysis of P024 trial/POL trial, no relapse was observed with a median follow up of 61.3 months in the 24 patients in the PEPI 0 category. PEPI 0 as a prognostic marker on therapy therapy is being validated prospectively in the A011106 trial. An improvement in the rate of PEPI 0 following neoadjuvant PD 0332991 and anastrozole compared to the historical control would be of great interest for the design of adjuvant trials of PD 0332991.

1.9.7 Assessment of Serum Estradiol Level Before and Following PD 0332991

The goal of this study is to ensure that PD 0332991 does not interact with anastrozole in reducing serum estradiol levels in addition to monitor changes in estradiol in premenopausal women being treated with goserelin, anastrozole before and after PD 0332991 during the course of therapy. Since there are no obvious theoretical pharmacokinetic interactions between the two agents, we do not anticipate a change in serum estradiol levels following PD 0332991 administration. Blood will be collected from premenopausal women for sensitive estradiol measurement, to be done at the clinical laboratory, at baseline, end of 4 weeks of anastrozole (or in combination with goserelin), Cycle 3 Day 1, and at time of surgery. In patients on goserelin because of initial premenopausal status prior to pre-registration, estradiol level must be in the postmenopausal range per institutional standard after Cycle 0 therapy to be eligible for the study drug therapy on this trial. In addition, these patients should go off study drug therapy and be considered inevaluable for the primary endpoint if the estradiol level rose to premenopausal range on Cycle 3 Day 1 or at Surgery or any time during Cycles 1-4 of treatment.

2.0 OBJECTIVES

Since surgery occurs 3 to 5 weeks following the last dose of PD 0332991 in order to avoid complications related to PD 0332991-induced myelosuppression, we propose to assess the primary endpoint of complete cell cycle arrest (Ki67 $\leq$ 2.7%) on C1D15 while patients are receiving PD 0332991. The value of Ki67 on C1D15 and at surgery will provide data on the

potential reversibility of Ki67 decrease when off PD 0332991 for at least 3 weeks. These data may help to hypothesize regarding the duration of the biologic effect of PD 0332991.

Amendment #5:
Please note that as of Amendment #5, a fifth cycle of daily PD 0332991 (consisting of 10-12 days of treatment, plus continued daily anastrozole) is added prior to surgery based on preliminary analysis of the first 18 patients showing an increase in tumor Ki67 at the time of surgery which was performed 3 to 5 weeks after the last dose of PD 0332991 (Cycle 4 Day 21). This data indicates that PD 0332991 has a reversible effect on cell cycle arrest. However, alternative explanations exist—for example, the emergence of resistance clones. To invesitigate these possibilities, we propose to re-initiate PD 0332991 (and continue anastrozole) for patients whose blood counts and other AEs recover sufficiently after 4 complete neoadjuvant cycles of PD 0332991 + anastrozole, and treat for an additional 10 to 12 days, with the last dose of PD 0332991 and anastrozole being administered on the day before surgery. Based on previous phase I studies, the mean ANC within 10-12 days of treatment is between 2.2 k-4.2 k/mcL (personal communication with Pfizer), therefore we expect majority of our patients will be able to complete Cycle 5. In fact, a neoadjuvant study consisting of 14 days of aromatase inhibitor +/- PD 0332991 before surgery is ongoing, and none of the first 9 patients randomized to receive PD 0332991 had ANC < 1,000 on Day 10 (personal communication with Pfizer).

2.1 Primary Objective

1. To determine the rate of complete cell cycle arrest, defined by Ki67 ≤2.7%, following 2 weeks (C1D15) of neoadjuvant PD 0332991 in combination with anastrozole in women with clinical stage II or III ER+/HER2- breast cancer without PIK3CA hot spot mutation (PIK3CA Wild Type Cohort).

2. To determine the rate of complete cell cycle arrest, defined by Ki67 ≤2.7%, following 2 weeks (C1D15) of neoadjuvant PD 0332991 in combination with anastrozole in women with clinical stage II or III ER+/HER2- endocrine-resistant breast cancer.

2.2 Secondary Objectives

1. To determine the rate of complete cell cycle arrest, defined by Ki67 ≤2.7%, following 2 weeks (C1D15) of neoadjuvant PD 0332991 in combination with anastrozole in women with clinical stage II or III ER+/HER2- breast cancer with PIK3CA hot spot mutation.

2. To determine the rate of complete cell cycle arrest, defined by Ki67 ≤2.7%, following 2 weeks (C1D15) of neoadjuvant PD 0332991 in combination with anastrozole in women with clinical stage II or III ER+/HER2- endocrine-resistant breast cancer.

The following objectives will be assessed in PIK3CA mutant cohort and in PIK3CA Wild Type (WT) cohort combined or separately, and assessed separately in the endocrine resistant cohort.

3. To compare the rate of complete cell cycle arrest between C1D1 and C1D15

4. To assess Ki67 level on serially collected tumor specimens (baseline, C1D1, C1D15, and surgery)

5. To determine the rate of PEPI 0 score with the study drug therapy

6. To determine the pathologic complete response (pCR) rate of study drug therapy

7. To assess the rate of clinical response and radiologic response in the neoadjuvant setting

8. To determine the safety profile (on therapy and post 30 days of therapy completion) of the study therapy during 4 months of neoadjuvant therapy.

9. To determine the safety profile of the study therapy during 2 years of adjuvant therapy.

10. To assess the concentrations of anastrozole prior to and 90 minutes following anastrozole (without PD 0332991) on Cycle 1 Day 1 and to compare these concentrations to the concentrations of anastrozole prior to and 90 minutes following both anastrozole and PD 0332991 on Cycle 1 Day 15

11. To assess the concentration of PD 0332991 prior to and 90 minutes following both anastrozole and PD 0332991 on Cycle 1 Day 15

12. To assess the long term outcomes of patients treated in this trial.

2.3 Exploratory Objectives

The following objectives will be assessed in PIK3CA mutant cohort and in PIK3CA WT cohort combined or separately, and assessed separately in the endocrine resistant cohort.

1. To assess tumor cell apoptosis index and senescence markers on serially collected tumor specimens

2. To examine the pharmacodynamic effect of PD 0332991 in combination with anastrozole using serially collected tumor specimens (baseline, C1D1, 2 weeks on combination therapy, and surgery)

3. To explore molecular mechanisms which could affect tumor response to the combination PD 0332991 and anastrozole by tumor genomic, transcriptomic, and proteomic analysis

4. To document pathologic staging (tumor size, lymph node status) following

neoadjuvant chemotherapy in patients who had tumor Ki67>10% on C1D15 and elected to receive neoadjuvant chemotherapy

5. To explore circulating markers predictive of cancer recurrence.

3.0 PATIENT SELECTION

There are three cohorts of patients for this trial: 1) PIK3CA Wild Type Cohort, 2) PIK3CA Mutant Cohort, and 3) Endocrine Resistant Cohort. **As of Amendment #7, enrollment to the PIK3CA Wild Type and Mutant Cohorts is closed; patients may enroll to the Endocrine Resistant Cohort for neoadjuvant treatment and to the Adjuvant Cohort for adjuvant treatment.**

Applicable to the PIK3CA Wild Type and Mutant Cohort:
Patients who were pre-registered to NCI 9170 trial (Phase II Trial of Neoadjuvant MK-2206 in Combination with either Anastrozole if Postmenopausal or Anastrozole and Goserelin if Premenopausal in Women with Clinical Stage 2 or 3 PIK3CA Mutant Estrogen Receptor Positive and HER2 Negative Invasive Breast Cancer), started anastrozole (or anastrozole plus goserelin if premenopausal) ≤ 6 weeks, and were found negative for PIK3CA hotspot mutations are eligible to be screened for the wild type cohort.

In institutions without NCI9170 open, or after completion of enrollment to NCI9170 in institutions where it is open, patients will be pre-registered to this trial and those with PIK3CA mutations will be enrolled to the PIK3CA mutant cohort.

Applicable to the Endocrine Resistant Cohort:
Pre-registration is not required for patients to be enrolled in the endocrine resistant cohort, as PIK3CA mutation status will not be assessed for those patients.

Patients who are potentially eligible to receive adjuvant treatment with PD 0332991 are required to be screened using the eligibility criteria in Section 3.5.

3.1 Pre-registration Eligibility Criteria for the PIK3CA Mutant Cohort

Note: enrollment to this cohort is CLOSED as of Amendment #7.

3.1.1 Inclusion Criteria

1. Clinical T2-T4c, any N, M0 invasive ER+ (Allred Score of 6-8) and HER2 negative (0 or 1+ by IHC or FISH negative for amplification) breast cancer, by AJCC 7th edition clinical staging, with the goal being surgery to completely excise the tumor in the breast and the lymph node.

   Note: Patients with invasive ER+ (Allred Score of 6-8) HER2- breast cancer or DCIS in the contralateral breast the patient are eligible

2. Female ≥18 years of age.

3. ECOG performance status of 0, 1 or 2 (Appendix A).

4. Life expectancy > 4 months.

5. If premenopausal, patient must be willing to comply with pregnancy requirements laid out in Section 5.5.

6. Adequate organ and marrow function as defined below:
   a. leukocytes ≥ 3,000/mcL
   b. absolute neutrophil count ≥ 1,500/mcL
   c. platelets ≥ 100,000/mcL
   d. total bilirubin ≤ upper normal institutional limits
   e. AST(SGOT)/ and ALT(SGPT) ≤ 2.5 X institutional upper limit normal
   f. Creatinine ≤ upper normal institutional limits

7. Able to understand and willing to sign an IRB-approved written informed consent document.

3.1.2 Exclusion Criteria

1. Prior treatment of this cancer including:
   a. Surgery,
   b. Radiation therapy,
   c. Chemotherapy,
   d. Biotherapy,
   e. Hormonal therapy
   f. Investigational agent prior to study entry.

2. Receiving any other investigational agents.

3. Prior therapy with any Cdk4 inhibitor.

4. Any of the following in the previous 6 months:
   a. myocardial infarction
   b. severe/unstable angina
   c. coronary/peripheral artery bypass graft
   d. symptomatic congestive heart failure
   e. cerebrovascular accident
   f. transient ischemic attack
   g. symptomatic pulmonary embolism.

5. Uncontrolled intercurrent illness including, but not limited to:
   a. ongoing or active infection
   b. symptomatic congestive heart failure

c. unstable angina pectoris
d. uncontrolled symptomatic cardiac arrhythmia,
e. psychiatric illness/social situations that would limit compliance with study requirements.

6. Pregnant/nursing.

7. Unwilling to employ adequate contraception.

8. Known HIV-positive on combination antiretroviral therapy.

   NOTE: HIV-positive patients on combination antiretroviral therapy are ineligible because of the potential for pharmacokinetic interactions with PD 0332991. In addition, these patients are at increased risk of lethal infections when treated with marrow-suppressive therapy.

9. Evidence of inflammatory cancer (clinical presentation of skin erythema involving more than one third of the breast or pathological evidence of dermal lymphatic involvement)

10. Known metastatic disease.

11. Current use of anticoagulation therapy.

12. Previous excisional biopsy of the breast cancer or sentinel lymph node biopsy.

13. Any condition that impairs patient's ability to swallow PD 0332991 tablets (*e.g.*, gastrointestinal tract disease resulting in an inability to take oral medication or a requirement for IV alimentation, prior surgical procedures affecting absorption)

14. History of allergic reactions attributed to compounds of similar chemical or biologic composition to PD 0332991 or other agents used in the study.

15. Corrected QT (QTc) interval >470 msec.

3.2 Registration Eligibility Criteria for the PIK3CA Mutant Cohort

Note: enrollment to this cohort is CLOSED as of Amendment #7.

3.2.1 Inclusion Criteria

The criteria below must be met for registration onto the study in addition to the pre-registration criteria, except treatment with endocrine therapy for this cancer is allowed prior to registration.

1. For the PIK3CA mutant cohort: tumor PIK3CA mutation present

2. In premenopausal women, serum estradiol level in postmenopausal range ≤ 7 days prior to registration.

3.2.2 Exclusion Criteria

The criteria below must be met for registration onto the study in addition to the pre-registration criteria.

1. Current use or anticipated need for food or drugs that are known strong CYP3A4 inhibitors (i.e. grapefruit juice, verapamil, ketoconazole, miconazole, itraconazole, posaconazole, erythromycin, clarithromycin, telithromycin, indinavir, saquinavir, ritonavir, nelfinavir, lopinavir, atazanavir, amprenavir, fosamprenavir, nefazodone, diltiazem, and delavirdine) or inducers (i.e. dexamethasone, glucocorticoids, progesterone, rifampin, phenobarbital, St. John's wort).

3.3 Eligibility Criteria for the PIK3CA Wild Type Cohort

Note: enrollment to this cohort is CLOSED as of Amendment #7.

3.3.1 Inclusion Criteria

1. Clinical T2-T4c, any N, M0 invasive ER+ (Allred Score of 6-8) and HER2 negative (0 or 1+ by IHC or FISH negative for amplification) breast cancer, by AJCC 7th edition clinical staging, with the goal being surgery to completely excise the tumor in the breast and the lymph node.

   Note: Patients with invasive ER+ (Allred Score of 6-8) HER2- breast cancer or DCIS in the contralateral breast the patient are eligible

2. For the PIK3CA wild type cohort: tumor PIK3CA mutation absent

   Note that if a patient did not have sufficient research tissue for PIK3CA sequencing at pre-registration or if PIK3CA sequencing result is delayed, she could be registered and enrolled on the PD991 trial without assigning to a particular cohort at the time of enrollment. PIK3CA sequencing will be performed in the future on tumors collected at subsequent time points to assign the treatment cohort or when the PIK3CA sequencing data is available.

3. For the endocrine resistant cohort: Ki67 > 10% by central testing at Washington University AMP laboratory from a tumor biopsy performed after at least 2 weeks on neoadjuvant endocrine therapy.

a. Note that prior neoadjuvant endocrine therapy could include any endocrine therapy (including aromatase inhibitor, tamoxifen, fulvestrant) alone or in combination, or endocrine therapy in combination with any investigational agent that is not a Cdk 4/6 inhibitor.
b. Patients who had a Day 17 Ki67 > 10% from the NCI9170 trial are eligible for the endocrine resistant cohort.
c. Note that enrollment to the endocrine resistant cohort will depend on the funding availability. Please contact the study chair before enrolling patients to this cohort.

4. Female ≥18 years of age.

5. ECOG performance status of 0, 1 or 2 (Appendix A).

6. Life expectancy > 4 months.

7. If premenopausal, patient must be willing to comply with pregnancy requirements laid out in Section 5.5.

8. Adequate organ and marrow function as defined below:
   a. Leukocytes ≥ 3,000/mcL
   b. absolute neutrophil count ≥ 1,500/mcL
   c. platelets ≥ 100,000/mcL
   d. total bilirubin ≤ upper normal institutional limits
   e. AST(SGOT)/ and ALT(SGPT) ≤ 2.5 X institutional upper limit normal
   f. Creatinine ≤ upper normal institutional limits

9. In premenopausal women, serum estradiol level in postmenopausal range ≤ 7 days prior to registration.

10. Able to understand and willing to sign an IRB-approved written informed consent document.

3.3.2 Exclusion Criteria

1. Prior treatment of this cancer including:
   a. Surgery,
   b. Radiation therapy,
   c. Chemotherapy,
   d. Biotherapy,
   e. Hormonal therapy
   f. Investigational agent prior to study entry.

2. Receiving any other investigational agents.

3. Prior therapy with any Cdk4 inhibitor.

4. Any of the following in the previous 6 months:
   a. myocardial infarction
   b. severe/unstable angina
   c. coronary/peripheral artery bypass graft
   d. symptomatic congestive heart failure
   e. cerebrovascular accident
   f. transient ischemic attack
   g. symptomatic pulmonary embolism.

5. Uncontrolled intercurrent illness including, but not limited to:
   a. ongoing or active infection
   b. symptomatic congestive heart failure
   c. unstable angina pectoris
   d. uncontrolled symptomatic cardiac arrhythmia,
   e. psychiatric illness/social situations that would limit compliance with study requirements.

6. Pregnant/nursing.

7. Unwilling to employ adequate contraception.

8. Known HIV-positive on combination antiretroviral therapy.

   NOTE: HIV-positive patients on combination antiretroviral therapy are ineligible because of the potential for pharmacokinetic interactions with PD 0332991. In addition, these patients are at increased risk of lethal infections when treated with marrow-suppressive therapy.

9. Evidence of inflammatory cancer (clinical presentation of skin erythema involving more than one third of the breast or pathological evidence of dermal lymphatic involvement)

10. Known metastatic disease.

11. Current use of anticoagulation therapy.

12. Previous excisional biopsy of the breast cancer or sentinel lymph node biopsy.

13. Any condition that impairs patient's ability to swallow PD 0332991 tablets (*e.g.*, gastrointestinal tract disease resulting in an inability to take oral medication or a requirement for IV alimentation, prior surgical procedures affecting absorption)

14. History of allergic reactions attributed to compounds of similar chemical or biologic composition to PD 0332991 or other agents used in the study.

15. Corrected QT (QTc) interval >470 msec.

16. Current use or anticipated need for food or drugs that are known strong CYP3A4 inhibitors (i.e. grapefruit juice, verapamil, ketoconazole, miconazole, itraconazole, posaconazole, erythromycin, clarithromycin, telithromycin, indinavir, saquinavir, ritonavir, nelfinavir, lopinavir, atazanavir, amprenavir, fosamprenavir, nefazodone, diltiazem, and delavirdine) or inducers (i.e. dexamethasone, glucocorticoids, progesterone, rifampin, phenobarbital, St. John's wort).

3.4 Eligibility Criteria for the Endocrine Resistant Cohort

3.4.1 Inclusion Criteria

1. Clinical T2-T4c at diagnosis or screening, any N, M0 invasive ER+ (Allred Score at least 3 or > 1% ER positivity) and HER2 negative (0 or 1+ by IHC or FISH negative or equivocal) breast cancer, by AJCC 7th edition clinical staging, with the goal being surgery to completely excise the tumor in the breast and the lymph node.

   Note: Patients with invasive breast cancer that is ER pos, HER2 neg or equivocal or DCIS in the contralateral breast are eligible; multi-focal diseases are not excluded. The dominant lesion will be followed per protocol.

2. Ki67 > 10% by central testing at Washington University AMP laboratory from a tumor biopsy performed after at least 2 weeks on neoadjuvant endocrine therapy.

   If Ki67 is > 10% by local testing, the Ki67 slide and H&E slide need to be reviewed by the study pathologist to confirm eligibility (discuss with Study Chair). For patients external to Washington University, please contact the Washington University coordinator by email so that a screening ID# can be assigned prior to shipment of the slides.

   Note that prior neoadjuvant endocrine therapy could include any endocrine therapy (including aromatase inhibitor, tamoxifen, fulvestrant) alone or in combination, or endocrine therapy in combination with any investigational agent that is not a Cdk 4/6 inhibitor.

3. Female ≥18 years of age.

4. ECOG performance status of 0, 1 or 2 (Appendix A).

5. Pre- or post-menopausal women are eligible

   If premenopausal, patient must be willing to comply with pregnancy requirements laid out in Section 5.5 and agrees with GnRH agonist therapy for ovarian suppression during the study.

6. Adequate organ and marrow function as defined below:
   a. Leukocytes ≥ 3,000/mcL
   b. Absolute neutrophil count ≥ 1,500/mcL
   c. Platelets ≥ 100,000/mcL
   d. Total bilirubin ≤ upper normal institutional limits
   e. AST(SGOT)/ and ALT(SGPT) ≤ 2.5 X institutional upper limit normal
   f. Creatinine ≤ upper normal institutional limits

7. Able to understand and willing to sign an IRB-approved written informed consent document.

3.4.2 Exclusion Criteria

1. Prior treatment of this cancer including:
   a. Surgery
   b. Radiation therapy
   c. Chemotherapy

2. Receiving any other investigational agents.

3. Prior therapy with any Cdk4 inhibitor.

4. Any of the following in the previous 6 months:
   a. myocardial infarction
   b. severe/unstable angina
   c. coronary/peripheral artery bypass graft
   d. symptomatic congestive heart failure
   e. cerebrovascular accident
   f. transient ischemic attack
   g. symptomatic pulmonary embolism.

5. Uncontrolled intercurrent illness including, but not limited to:
   a. ongoing or active infection
   b. symptomatic congestive heart failure
   c. unstable angina pectoris
   d. uncontrolled symptomatic cardiac arrhythmia,
   e. psychiatric illness/social situations that would limit compliance with study requirements.

6. Pregnant/nursing.

7. Unwilling to employ adequate contraception.

8. Known HIV-positive on combination antiretroviral therapy.

   NOTE: HIV-positive patients on combination antiretroviral therapy are ineligible because of the potential for pharmacokinetic interactions with PD 0332991. In addition, these patients are at increased risk of lethal infections when treated with marrow-suppressive therapy.

9. Known metastatic disease.

10. Current use of anticoagulation therapy.

11. Previous excisional biopsy of the breast cancer or sentinel lymph node biopsy.

12. Any condition that impairs patient's ability to swallow PD 0332991 tablets (*e.g.*, gastrointestinal tract disease resulting in an inability to take oral medication or a requirement for IV alimentation, prior surgical procedures affecting absorption)

13. History of allergic reactions attributed to compounds of similar chemical or biologic composition to PD 0332991 or other agents used in the study.

14. Corrected QT (QTc) interval >470 msec.

15. Current use or anticipated need for food or drugs that are known strong CYP3A4 inhibitors (i.e. grapefruit juice, verapamil, ketoconazole, miconazole, itraconazole, posaconazole, erythromycin, clarithromycin, telithromycin, indinavir, saquinavir, ritonavir, nelfinavir, lopinavir, atazanavir, amprenavir, fosamprenavir, nefazodone, diltiazem, and delavirdine) or inducers (i.e. dexamethasone, glucocorticoids, progesterone, rifampin, phenobarbital, St. John's wort). See Appendix H

3.5 Eligibility Criteria for the Adjuvant Cohort

3.5.1 Inclusion Criteria

1. Derived benefit from PD 0332991 in the neoadjuvant setting in this trial. This includes the 26 patients who achieved complete cell cycle arrest only after the addition of PD 0332991 (C1D1 Ki67 >2.7% and C1D15 Ki67 ≤ 2.7%) from the main study (PIK3CA WT, mutant, or unknown cohorts) as well as any patients who have a Ki67 ≤ 10% on C1D15 biopsy in the endocrine resistant cohort.

2. ECOG performance status of 0, 1 or 2 (Appendix A).

3. If premenopausal, patient must be willing to comply with pregnancy requirements laid out in Section 5.5.

4. Adequate organ and marrow function as defined below:
   a. leukocytes ≥ 3,000/mcL
   b. absolute neutrophil count ≥ 1,500/mcL
   c. platelets ≥ 100,000/mcL
   d. total bilirubin ≤ upper normal institutional limits
   e. AST(SGOT)/ and ALT(SGPT) ≤ 2.5 X institutional upper limit normal
   f. Creatinine ≤ upper normal institutional limits

5. Underwent surgery of the breast and axilla for curative intent.

6. At least 4 weeks post completion of adjuvant chemotherapy and radiation therapy if indicated.

7. Patients who already started on adjuvant hormonal therapy are eligible under the following conditions:
   a. For the 26 patients who enrolled in the initial cohorts and derived benefit from neoadjuvant PD 0332991 (C1D1 Ki67 >2.7% and C1D15 Ki67 ≤ 2.7%), adjuvant PD 0332991 should be initiated as soon as possible if adjuvant hormonal therapy has been initiated and the patient has completed radiation if indicated.
   b. For patients who enrolled in the endocrine resistant cohort and derived benefit from neoadjuvant PD 0332991 (C1D15 Ki67 ≤ 10%), adjuvant PD 0332991 should be initiated within 6 months or sooner after initation of adjuvant hormonal therapy.

8. Able to understand and willing to sign an IRB-approved written informed consent document.

3.5.2 Exclusion Criteria

1. Any of the following in the previous 6 months:
   a. myocardial infarction
   b. severe/unstable angina
   c. coronary/peripheral artery bypass graft
   d. symptomatic congestive heart failure
   e. cerebrovascular accident
   f. transient ischemic attack
   g. symptomatic pulmonary embolism.

2. Uncontrolled intercurrent illness including, but not limited to:

a. ongoing or active infection
b. symptomatic congestive heart failure
c. unstable angina pectoris
d. uncontrolled symptomatic cardiac arrhythmia
e. Psychiatric illness/social situations that would limit compliance with study requirements.

3. Pregnant/nursing.

4. Unwilling to employ adequate contraception.

5. Known HIV-positive on combination antiretroviral therapy.

   NOTE: HIV-positive patients on combination antiretroviral therapy are ineligible because of the potential for pharmacokinetic interactions with PD 0332991. In addition, these patients are at increased risk of lethal infections when treated with marrow-suppressive therapy.

6. Known metastatic disease.

7. Any condition that impairs patient's ability to swallow PD 0332991 tablets (e.g., gastrointestinal tract disease resulting in an inability to take oral medication or a requirement for IV alimentation, prior surgical procedures affecting absorption)

8. History of allergic reactions attributed to compounds of similar chemical or biologic composition to PD 0332991 or other agents used in the study.

9. Corrected QT (QTc) interval >470 msec.

10. Current use or anticipated need for food or drugs that are known strong CYP3A4 inhibitors (i.e. grapefruit juice, verapamil, ketoconazole, miconazole, itraconazole, posaconazole, erythromycin, clarithromycin, telithromycin, indinavir, saquinavir, ritonavir, nelfinavir, lopinavir, atazanavir, amprenavir, fosamprenavir, nefazodone, diltiazem, and delavirdine) or inducers (i.e. dexamethasone, glucocorticoids, progesterone, rifampin, phenobarbital, St. John's wort). See Appendix H

3.6 Inclusion of Women and Minorities

Women and members of all races and ethnic groups are eligible for this trial.

4.0 PRE-REGISTRATION AND REGISTRATION PROCEDURES

4.1 Pre-Registration

This subsection applies only to patients enrolling to the PIK3CA Mutant and WT cohorts. Enrollment to those cohorts is closed as of Amendment #7.

Patients will be pre-registered to the NCI 9170 trial if it is active for patient enrollment at the participating institution unless patient is not an appropriate candidate for the NCI9170 trial per treating physician. Otherwise, patients will be pre-registered through this trial using the following procedures for PIK3CA sequencing. No pre-registration is required for the Endocrine Resistant Cohort.

Patients must not start any protocol intervention prior to pre-registration through the Siteman Cancer Center. All eligible and consenting patients will be pre-registered to this protocol for the purposes of PIK3CA sequencing; if mutation is identified, the patient will then be registered to the PIK3CA Mutant Cohort if eligible for study treatment. If mutation is not identified, the patient will then be registered to the PIK3CA Wild Type Cohort if eligible for study treatment. Pre-registration is not required for patients to be enrolled in the endocrine resistant cohort, as PIK3CA mutation status will not be assessed.

The following steps must be taken:

1. Confirmation of patient pre-registration eligibility by Washington University
2. Pre-Registration of patient in the Siteman Cancer Center database
3. Assignment of unique patient number (UPN)

Once the patient has been entered in the Siteman Cancer Center database, the WUSM coordinator will forward verification of enrollment and the UPN via email.

4.1.1 Confirmation of Patient Eligibility for Pre-Registration

Confirm patient eligibility by scanning or faxing the information listed below to the research coordinator listed in the *Siteman Cancer Center Clinical Trials Core Protocol Procedures for Secondary Sites* packet and Caroline Bumb (cbumb@dom.wustl.edu) at least one business day prior to registering patient:

1. Your name and contact information (telephone number, fax number, and email address)

2. Your site PI's name, the registering MD's name, and your institution name
3. Patient's race, sex, and DOB
4. Three letters (or two letters and a dash) for the patient's initials
5. Currently approved protocol version date
6. Copy of signed consent form (patient name may be blacked out)
7. Planned date of sample shipment
8. Completed pre-registration eligibility checklist, signed and dated by a member of the study team
9. Copy of appropriate source documentation confirming patient eligibility

4.1.2 Patient Pre-Registration in the Siteman Cancer Center Database

Registrations may be submitted Monday through Friday between 8am and 5pm CT. Urgent late afternoon or early morning enrollments should be planned in advance and coordinated with the Washington University research coordinator. Registration will be confirmed by the research coordinator or his/her delegate by email or fax within one business day. Verification of eligibility and pre-registration should be kept in the patient chart.

Patients at all sites must be pre-registered through the Siteman Cancer Center database at Washington University.

4.1.3 Assignment of UPN

Each patient will be identified with a unique patient number (UPN) for this study. Patients will also be identified by first, middle, and last initials. If the patient has no middle initial, a dash will be used on the case report forms (CRFs). All data will be recorded with this identification number on the appropriate CRFs.

4.1.4 Additional Notes

- Pre-registration tests/procedures (see Section 10.0) must be completed within the guidelines specified on the test schedule.
- At the time of IRB submission, site should request several biopsy kits by contacting the Alliance Central Specimen Bank (address and phone numbers listed in Section 9.1).
- Following pre-registration and research tumor biopsy, patient may start Cycle 0 anastrozole (and goserelin if premenopausal) according to the instructions in Section 5.1.

4.2 Registration

The information in this section applies to patients enrolling to ALL cohorts.

Patients must not start any protocol intervention prior to registration through the Siteman Cancer Center.

The following steps must be taken before registering patients to this study:

1. Confirmation of patient eligibility by Washington University
2. Registration of patient in the Siteman Cancer Center database
3. Assignment of unique patient number (UPN) if not pre-registered through this trial

Once the patient has been entered in the Siteman Cancer Center database, the WUSM coordinator will forward verification of enrollment via email.

4.2.1 Confirmation of Patient Eligibility

Confirm patient eligibility collecting the information listed below and scanning and emailing to the research coordinator listed in the *Siteman Cancer Center Clinical Trials Core Protocol Procedures for Secondary Sites* packet and Caroline Bumb at cbumb@ wustl.edu at least one business day prior to registering patient:

1. Your name and contact information (telephone number, fax number, and email address)
2. Your site PI's name, the registering MD's name, and your institution name
3. Patient's race, sex, and DOB
4. Three letters (or two letters and a dash) for the patient's initials
5. Currently approved protocol version date
6. Copy of signed consent form (patient name may be blacked out)
7. Planned date of enrollment
8. Completed eligibility checklist (Appendix B), signed and dated by a member of the study team
9. Copy of appropriate source documentation confirming patient eligibility

4.2.2 Patient Registration in the Siteman Cancer Center Database

Registrations may be submitted Monday through Friday between 8am and 5pm CT. Urgent late afternoon or early morning enrollments should be planned in advance and coordinated with the Washington University research coordinator. Registration will be confirmed by the research coordinator or his/her delegate by email within one business day. Verification of eligibility and registration should be kept in the patient chart.

All patients at all sites must be registered through the Siteman Cancer Center database at Washington University.

4.2.3 Assignment of UPN

Each patient will be identified with a unique patient number (UPN) for this study. Patients will also be identified by first, middle, and last initials. If the patient has

no middle initial, a dash will be used on the case report forms (CRFs). All data will be recorded with this identification number on the appropriate CRFs.

5.0 TREATMENT PLAN

5.1 Neoadjuvant Treatment

Treatment will be administered on an outpatient basis. Patients will be instructed to make use of medication diaries (Appendices D and E) to act as a records for administration of PD 0332991 and anastrozole. Appropriate dose modifications are described in Section 6.0. No investigational or commercial agents or therapies other than those described below may be administered with the intent to treat the patient's malignancy.

<u>Cycle 0 (Only applicable for PIK3CA Mutant and WT cohorts)</u>
Before Cycle 1 Day 1 starts, patients who enrolled in the PIK3CA mutant or WT cohort should have completed Cycle 0. Cycle 0 therapy is 28 days of anastrozole (1mg PO daily) and, if premenopausal, goserelin 3.6mg SC every 28 days.

<u>Cycles 1-5</u>
Treatment with PD 0332991 combined with anastrozole (and goserelin if premenopausal) is to be 4 28-day cycles followed by a fifth cycle of 10-12 days duration consisting of daily PD 0332991 and anastrozole, with the last dose of both drugs to be given the day before surgery.

<table>
<tr><th colspan="5">REGIMEN DESCRIPTION</th></tr>
<tr><th>Agent</th><th>Dose</th><th>Route</th><th>Schedule</th><th>Cycle Length</th></tr>
<tr><td>PD 0332991</td><td>125 mg</td><td>PO</td><td>Daily
(Days 1-21)</td><td rowspan="3">Cycles 1-4: 28 days
Cycle 5: 10-12 days
Adjuvant treatment: 28 days</td></tr>
<tr><td>Anastrozole</td><td>1 mg</td><td>PO</td><td>Daily
(Days 1-28)</td></tr>
<tr><td>Goserelin*</td><td>3.6 mg</td><td>SC</td><td>Q28 days</td></tr>
<tr><td colspan="5">* only if premenopausal; although it is preferred to be given on Day 1 of each cycle, it may be administered any day of the cycle to accommodate the Q28-day cycle for goserelin.</td></tr>
</table>

PD 0332991 should be taken with food. Anastrozole should be taken daily with or without food.

For both PD 0332991 and anastrozole:

- If a patient misses a day's dose entirely, she must be instructed not to make it up the next day but just take her regular dose the following day.
- If a patient vomits anytime after taking a dose, she must be instructed not to make it up but to resume subsequent does the next day as prescribed.
- If a patient inadvertently takes an extra dose during a day, she must be instructed to not take the next day's dose.

Special Considerations for Cycle 5

Cycle 5 Day 1 of PD 0332991 may be delayed by up to 3 weeks to allow ANC to recover to ≥ 1500/mcL and platelets to recover to ≥ 100,000/mcL and other treatment related AEs to recover to ≤ grade 1 as well as to accommodate surgery scheduling. Anastrozole is continued in all patients during the neoadjuvant period. Patients who receive goserelin should also continue goserelin every 28 days during the neoadjuvant period.

CBC+D is to be performed 3 days (±1 day) prior to surgery in patients who receive Cycle 5 therapy. Treatment with PD 0332991 should be interrupted if ANC < 1,000/mcL, platelets < 100,000/mcL, or patients present with any ≥ grade 2 treatment-related toxicities. If a patient needs to stop treatment prematurely in Cycle 5, she will have surgery on the scheduled date unless contraindicated, as the recovery of neutropenia is usually prompt after stopping PD 0332991 due to its mechanisms of action (investigator brochure). However, surgery may be rescheduled at the discretion of the treating physician and the study chair. Patients should continue with anastrozole until the day before surgery regardless of whether they complete Cycle 5 of PD 0332991.

Patients whose ANC and/or platelets do not recover within 3 weeks following the completion of Cycle 4 will proceed to surgery as planned (3 to 5 weeks following the last dose of PD 0332991 (Cycle 4 Day 21)). For these patients, anastrozole is to be continued until the day of surgery, with the last dose of anastrozole given the day before surgery.

5.2 Surgery

Standard surgery (breast and axillary lymph node surgery) will be performed per institutional standards 2-4 weeks following the completion (Day 28) of Cycle 4 in those who did not receive Cycle 5. In patients who receive Cycle 5, surgery occurs on Day 11, 12, or 13 of Cycle 5.

Note that tissue collection is mandatory at the time of surgery for all patients.

5.3 Post Surgery Therapy

Standard adjuvant treatment, including chemotherapy, hormonal therapy, and radiation if needed, are recommended after surgery at the discretion of the treating physician.

Patients who derived benefit from the combination therapy have the option of taking PD 0332991 in combination with anastrozole for 23 cycles after surgery and adjuvant chemotherapy and radiation if indicated. Patients are determined to have derived benefit if their Ki67 is ≤ 10% on C1D15 tumor biopsy if they are enrolled to the endocrine resistant cohort (including patients who enrolled prior to activation of Amendment #7) **OR** if they are among the 26 patients who enrolled initially to the main trial who required PD 0332991 to achieve complete cell cycle arrest.

PD 0332991 should be re-started at least 4 weeks after the completion of chemotherapy

and radiation therapy if these treatments were planned. Hormonal therapy may be started earlier (during radiation therapy) if desired.

Anastrozole is preferred in this trial to combine with PD 0332991 in the adjuvant setting as this is the same regimen used in the neoadjvuant setting, but alternative hormonal therapy such as another aromatase inhibitor or tamoxifen is allowed if patient could not tolerate adjuvant anastrozole. Goserelin should be administered if aromatase inhibitor is used in patients who are pre-menopausal determined by institutional standard.

After completion of adjuvant PD 0332991, patients should continue standard of care hormonal therapy (for example, anastrozole) to complete at least 5 years of adjuvant hormonal therapy. Further therapy after that is at the discretion of the treating physician.

5.4 General Concomitant Medication and Supportive Care Guidelines

While taking PD 0332991, patients should be instructed to avoid food or drugs that are known strong CYP3A4 inhibitors or inducers. Please refer to Appendix H for a list of prohibited medications.

No specific antidotes exist for the treatment of PD 0332991 overdose. Since renal excretion of PD 0332991 is minimal, the benefit of hemodialysis in the treatment of a PD 0332991 overdose is probably negligible. The treatment of overdose of PD 0332991 should consist of general supportive measures. If indicated, elimination of unabsorbed drug should be achieved by emesis or gastric lavage.

No prophylactic medications are required prior to administration of anastrozole or goserelin.

5.5 Women of Childbearing Potential

Women of childbearing potential (defined as women with regular menses, women with amenorrhea, women with irregular cycles, women using a contraceptive method that precludes withdrawal bleeding, and women who have had a tubal ligation) are required to have a negative serum pregnancy test within 7 days prior to the first dose of the study agent.

Women of childbearing potential are required to use two forms of acceptable contraception, including one barrier method, during participation in the study and for 90 days following the last dose of PD 0332991.

If a patient is suspected to be pregnant, all study drugs should be immediately discontinued. In addition a positive urine test must be confirmed by a serum pregnancy test. If it is confirmed that the patient is not pregnant, she may resume dosing.

5.6 Duration of Therapy

If at any time the constraints of this protocol are considered to be detrimental to the patient's health and/or the patient no longer wishes to continue protocol therapy, the protocol therapy should be discontinued and the reason(s) for discontinuation documented in the case report forms.

In the absence of treatment delays due to adverse events, neoadjuvant treatment may continue for 4 cycles plus the fifth short cycle or one of the following criteria applies:

- Documented and confirmed disease progression or Ki67>10 % on C1D15
- Death
- Adverse event(s) that, in the judgment of the investigator, may cause severe or permanent harm or which rule out continuation of study drug
- General or specific changes in the patient's condition render the patient unacceptable for further treatment in the judgment of the investigator
- Suspected pregnancy
- Serious non-compliance with the study protocol
- Lost to follow-up
- Patient withdraws consent
- Investigator removes the patient from study
- The Siteman Cancer Center decides to close the study

Adjuvant treatment (in patients eligible to receive it) may continue for up to 23 cycles or until one of the following criteria applies:

- Documented and confirmed disease progression
- Death
- Adverse event(s) that, in the judgment of the investigator, may cause severe or permanent harm or which rule out continuation of study drug
- General or specific changes in the patient's condition render the patient unacceptable for further treatment in the judgment of the investigator
- Suspected pregnancy
- Serious non-compliance with the study protocol (missing 50% dosing in any cycle due to non-compliance or missing study required procedures due to non-compliance determined by site investigator)
- Lost to follow-up
- Patient withdraws consent
- Investigator removes the patient from study
- The Siteman Cancer Center decides to close the study

Patients who prematurely discontinue treatment for any reason will be followed as indicated in the study calendar.

5.7 Treatment/Follow-up Decision Tree

Any patient who registers on study and then withdraws her consent prior to receiving any PD 0332991 will be considered a cancellation. No further follow-up is required. Future

treatment is at the discretion of their treating physician.

If a patient is found not to have fulfilled all the eligibility requirements after starting PD 0332991, she will be removed from study treatment. All study forms up to the point of study treatment discontinuation are to be submitted. These patients will be observed for AEs for 30 days following the last dose of PD 0332991 or the resolution of AEs at least possibly related to PD 0332991, whichever comes later. Future treatment is at the discretion of the treating physician. No further follow-up is required.

All patients except for those who withdrew consent for follow up or who refused to undergo surgery will be followed for 30 days after surgery, then yearly to document recurrence and survival status for 5 years or until recurrence whichever comes first (see Section 10.3).

Patients who received adjuvant PD 0332991 will be followed as described in Section 10.3 during adjuvant PD 0332991 therapy, then yearly following completion of PD 0332991 to complete a total of 5 years of follow-up post surgery. Should a patient discontinue adjuvant PD 0332991 early, she would be followed annually following the completion of PD 0332991 to get as close as possible to 5 years of post-surgery follow-up.

If protocol therapy was discontinued due to Ki67 > 10% on C1D15 or estradiol level in the premenopausal range in patients on goserelin, or discontinued PD 0332991 prior to completion of planned duration of therapy, the patient will be observed for AEs for 30 days following the last dose of PD 0332991 or the resolution of AEs at least possibly related to PD 0332991, then yearly to complete 5 years of follow up after surgery or until recurrence (whichever comes first). Neoadjuvant chemotherapy per physician choice is recommended before surgery. Pathologic response will be documented.

If there is physical evidence for clinical progression with bi-dimensional tape, ruler, or caliper tumor measurements of the primary tumor, a mammogram and/or ultrasound of the breast should be done to confirm/rule out progressive disease. If there is physical evidence for clinical progression with clinical assessment of the lymph node mass, an ultrasound of the axilla should be done to confirm/rule out progressive disease.

If progression is confirmed by ultrasound **OR** mammographic imaging, discontinue the study drug and operate as soon as possible or begin other anti-neoplastic approaches such as chemotherapy or radiation therapy at the treating physician's discretion. NOTE: Other anti-neoplastic approaches such as chemotherapy or radiation must not be administered while the patient is taking study drug.

For these patients:

- Obtain the serum and plasma samples (can be obtained at surgery)
- Obtain core biopsies (two frozen in OCT and two formalin-fixed) before the patient receives non-protocol chemotherapy (could be obtained at surgery)
- Complete case report form indicating whether the patient will go to immediate surgery or begin other anti-neoplastic approaches such as chemotherapy or

radiation therapy. If patient goes to immediate surgery, complete surgery and pathology case report forms.

- Follow-up for 30 days following the last dose of PD 0332991 or the resolution of AEs at least possibly related to PD 0332991, then yearly to complete 5 years of followup after surgery or until recurrence (whichever comes first).

If disease progression is not confirmed by ultrasound or mammographic imaging, study drug may be continued at the investigator's discretion, and the protocol followed as described. These patients are not eligible for adjuvant PD 0332991 therapy.

Clinical progression outside the primary site (i.e., the development of a new breast mass or the development of clinical suspicion for advanced disease) should lead to further imaging evaluation and if confirmed, study drug will be discontinued. Optional tumor biopsy and blood will be obtained prior to the subsequent neoadjuvant therapy or at the time of surgery is surgery is the next step. Subsequent management is at the investigator's discretion. The patient will go off study.

Patients in whom surgery is not performed will be observed for 30-60 days after last dose of study drug and will not undergo annual follow-up.

If a patient is found to be pregnant, the study drug will be discontinued immediately. The patient will be followed for adverse events through the end of this pregnancy. Future treatment decisions are at the discretion of the patient's treating physician.

If a patient withdraws consent at any point in this study, all study data should be submitted up to the point of consent withdrawal.

5.8 Duration of Follow-up

Patients who receive the study therapy will be observed post-surgery for 30-60 days or resolution of AEs, whichever comes later, then yearly for recurrence and survival status for 5 years. Patients who received adjuvant PD 0332991 will be followed as described in Section 10.3 during adjuvant PD 0332991 therapy, then yearly following completion of PD 0332991 to complete a total of 5 years of follow-up post-surgery.

6.0 DOSE DELAYS/DOSE MODIFICATIONS

Table 3. Dose Modification Table.

Dose Level	PD 0332991 Days 1-21 of each 28-day cycle	Anastrozole (daily)
1 (starting dose)	125 mg/day	1 mg/day
-1	100 mg/day	1 mg/day
-2	75 mg/day	1 mg/day
Note that PD 0332991 dose reduction below 75 mg/day is not allowed		

6.1 Dose Modifications for Anastrozole

No dose adjustment is permitted for anastrozole but interruptions are allowed per physician discretion. During adjuvant phase, switching to another hormonal therapy is allowed.

6.2 Dose Modifications for PD 0332991

Patients will be monitored for toxicity and the dose of PD 0332991 may be adjusted as indicated in Table 3. Dose reduction by 1, and if needed, 2 dose levels will be allowed depending on the type and severity of toxicity encountered (Table 4). Patients requiring more than 2 dose reductions will be discontinued from the study.

Recommended dose reductions for PD 0332991 are detailed in Table 4. Doses may be held as needed for toxicity resolution during a cycle. Doses omitted for toxicity are not replaced or restored within the same cycle (meaning that the cycle remains 28 days regardless of the number of doses of taken).

Treatment with PD 0332991 should be permanently discontinued if toxicity has not recovered to grade ≤ 2 within two weeks (including the scheduled 1-week off treatment period within a cycle).

Table 4. PD 0332991 Dose Modifications Based on Worst Treatment-Related Toxicity in the Previous Cycle

Worst Toxicity During Previous Cycle	Action	New Dose Level
Grade 4 neutropenia	Hold until ANC ≥ 1000/mm^3	Decrease by one dose level
Grade 4 thrombocytopenia	Hold until platelets ≥ 50,000	Decrease by one dose level
Grade 3 neutropenia associated with a documented infection or fever ≥ 38.5 ºC	Hold until ANC ≥ 1000/mm^3 without fever	Decrease by one dose level
Grade 3 neutropenia without fever	Hold until ANC ≥ 1000/mm^3	1st occurrence: resume at the same dose<br>2nd occurrence: decrease by one dose level<br>3rd occurrence: decrease by one dose level
Grade ≥ 3 non-hematologic toxicity (includes nausea, vomiting, diarrhea, and hypertension only if persisting despite maximal medical treatment)	Hold until recover to ≤ grade 1 (or ≤ grade 2 if investigator does not consider the AE a safety risk)	Decrease by one dose level
Inability to deliver at least 80% of the planned dose of PD 0332991 or anastrozole due to adverse events possibly related to study treatment		Decrease by one dose level
Grade ≥ 2 non-hematologic toxicity (except alopecia) that persists longer than 4 weeks despite		Decrease by one dose level

maximal supportive care and is unacceptable to patient and/or investigator		

6.2.1 Dose Adjustments Due to QTc Prolongation

Any patients who develops new grade 2 or greater ECG QT corrected interval prolonged at any time during the study will need to have the ECG repeated immediately for confirmation.

Grade 2: no adjustments; continue at same dose level
Grade 3 (reversible cause identified and corrected): withhold treatment until QTc ≤ 470 msec, then resume treatment at the same dose level
Grade 3 (no reversible cause identified): withhold treatment until QTc ≤ 470 msec, then decrease PD 0332991 by one dose level
Grade 4: permanently discontinue PD 0332991

6.3 Re-Treatment Criteria for Neoadjuvant Cycles 1-4 and Adjuvant Therapy

A new cycle of treatment with PD 0332991 may begin only if:

- ANC ≥1,000/mcL.
- Platelet count ≥50,000/mcL.
- Non-hematologic toxicities have returned to baseline or Grade ≤1 severity (or, at the Investigator's discretion, Grade ≤2 if not considered a safety risk for the patient).

Criteria for dose interruption within cycle:

- ANC <1,000/mcL.
- Platelet count <50,000/mcL.

Re-treatment within the cycle may only be started when ANC ≥1,000/mcL and platelet count ≥50,000/mcL.

Doses omitted for toxicity within a cycle are not replaced or restored within the same cycle (meaning that the cycle remains 28 days regardless of the number of doses of taken).

If these conditions are not met, anastrozole treatment may be continued but treatment with PD 0332991 must be delayed by one week. If, after a one-week delay, all toxicities have recovered within the limits described above, treatment with PD 0332991 can be resumed.

If the patient has not recovered after 2 weeks (including the scheduled 1-week off treatment period within a cycle) despite dose reduction to the lowest dose level, treatment with PD 0332991 will be permanently discontinued.

Adjuvant PD 0332991 may be interrupted for a maximum of 4 weeks due to intercurrent illness or surgery (discuss with study chair).

Anastrozole is administered in a continuous regimen and therefore no re-treatment criteria apply.

6.4 Re-Treatment Criteria for Cycle 5

Cycle 5 Day 1 with PD 0332991 may begin only if:

- ANC ≥1,500/mcL
- Platelet count ≥100,000/mcL
- Non-hematologic toxicities have returned to baseline or Grade ≤1 severity (or, at the Investigator's discretion, Grade ≤2 if not considered a safety risk for the patient).

If these conditions are not met, anastrozole treatment will be continued but treatment with PD 0332991 will be delayed by one to three weeks. If all toxicities have recovered within the limits described above within 3 weeks, treatment with PD 0332991 for Cycle 5 will be initiated. If the patient has not recovered after a 3 week delay, treatment with PD 0332991 will be permanently discontinued and patients should proceed to surgery within 3 to 5 weeks after the last dose of PD0332991 (Cycle 4 Day 21) if possible.

Anastrozole is administered in a continuous regimen and therefore no re-treatment criteria apply.

7.0 REGULATORY AND REPORTING REQUIREMENTS

The entities providing oversight of safety and compliance with the protocol require reporting as outline below.

The Washington University Human Research Protection Office (HRPO) requires that all events meeting the definition of unanticipated problem or serious noncompliance be reported as outlined in Section 7.2.

The FDA requires that all serious and unexpected adverse events be reported as outlined in Section 7.4. In addition, any fatal or life-threatening adverse experiences where there is a reasonable possibility of relationship to study intervention must be reported.

Pfizer requires that all events be reported as outlined in Section 7.7.

7.1 Definitions

7.1.1 Adverse Events (AEs)

Definition: any unfavorable medical occurrence in a human subject including any abnormal sign, symptom, or disease.

Grading: the descriptions and grading scales found in the revised NCI Common Terminology Criteria for Adverse Events (CTCAE) version 4.0 will be utilized for all toxicity reporting. A copy of the CTCAE version 4.0 can be downloaded from the CTEP website.

Attribution (relatedness), Expectedness, and Seriousness: the definitions for the terms listed that should be used are those provided by the Department of Health and Human Services' Office for Human Research Protections (OHRP). A copy of this guidance can be found on OHRP's website:
http://www.hhs.gov/ohrp/policy/advevntguid.html

7.1.2 Serious Adverse Event (SAE)

Definition: any adverse drug experience occurring at any dose that results in any of the following outcomes:

- Death
- A life-threatening adverse drug experience
- Inpatient hospitalization or prolongation of existing hospitalization
- A persistent or significant disability/incapacity (i.e., a substantial disruption of a person's ability to conduct normal life functions)
- A congenital anomaly/birth defect
- Any other experience which, based upon appropriate medical judgment, may jeopardize the subject and may require medical or surgical intervention to prevent one of the outcomes listed above

All unexpected SAEs must be reported to the FDA.

7.1.3 Unexpected Adverse Experience

Definition: any adverse drug experience, the specificity or severity of which is not consistent with the current investigator brochure (or risk information, if an IB is not required or available).

Events that are both serious AND unexpected must be reported to the FDA.

7.1.4 Life-Threatening Adverse Experience

Definition: any adverse drug experience that places the subject (in the view of the investigator) at immediate risk of death from the reaction as it occurred, i.e., it does not include a reaction that, had it occurred in a more severe form, might have caused death.

Life-threatening adverse experiences must be reported to the FDA.

7.1.5 Unanticipated Problems

Definition:

- unexpected (in terms of nature, severity, or frequency) given (a) the research procedures that are described in the protocol-related documents, such as the IRB-approved research protocol and informed consent document; and (b) the characteristics of the subject population being studied;
- related or possibly related to participation in the research (in this guidance document, possibly related means there is a reasonable possibility that the incident, experience, or outcome may have been caused by the procedures involved in the research); and
- suggests that the research places subjects or others at a greater risk of harm (including physical, psychological, economic, or social harm) than was previously known or recognized.

7.1.6 Noncompliance

Definition: failure to follow any applicable regulation or institutional policies that govern human subjects research or failure to follow the determinations of the IRB. Noncompliance may occur due to lack of knowledge or due to deliberate choice to ignore regulations, institutional policies, or determinations of the IRB.

7.1.7 Serious Noncompliance

Definition: noncompliance that materially increases risks, that results in substantial harm to subjects or others, or that materially compromises the rights or welfare of participants.

7.1.8 Protocol Exceptions

Definition: A planned deviation from the approved protocol that are under the research team's control. Exceptions apply only to a single participant or a singular situation.

Local IRB Pre-approval of all protocol exceptions must be obtained prior to the event. For secondary sites, the Washington University PI will issue approval of the exception, but it must also be submitted to the local IRB with documentation of approval forwarded to Washington University. HRPO approval is not required for protocol exceptions occurring at secondary sites.

7.2 Reporting to the Human Research Protection Office (HRPO) at Washington University

The PI is required to promptly notify the IRB of the following events:

- Any unanticipated problems involving risks to participants or others which occur at WU, any BJH or SLCH institution, or that impacts participants or the conduct of the study.
- Noncompliance with federal regulations or the requirements or determinations of the IRB.
- Receipt of new information that may impact the willingness of participants to participate or continue participation in the research study.

These events must be reported to the IRB within **10 working days** of the occurrence of the event or notification to the PI of the event. The death of a research participant that qualifies as a reportable event should be reported within **1 working day** of the occurrence of the event or notification to the PI of the event.

7.3 Reporting to the Quality Assurance and Safety Monitoring Committee (QASMC) at Washington University

The PI is required to notify the QASMC of any unanticipated problem occurring at WU or any BJH or SLCH institution that has been reported to and acknowledged by HRPO as reportable. (Unanticipated problems reported to HRPO and withdrawn during the review process need not be reported to QASMC.)

QASMC must be notified within **10 days** of receipt of IRB acknowledgment via email to a QASMC auditor.

7.4 Reporting Requirements for Secondary Sites

The research team at each secondary site is required to promptly notify the Washington University PI and research coordinator of all reportable events (as described in Section 7.6) within **1 working day** of the occurrence of the event or notification of the secondary site's PI of the event. This notification may take place via email if there is not yet enough information for a formal written report (using an FDA MedWatch form). A formal written report must be sent to the Washington University PI and research coordinator within **10 working days** of the occurrence of the event or notification of the secondary site's PI of the event. The death of a research participant that qualifies as a reportable event should be reported within **1 working day** of the occurrence of the event or notification of the secondary site's PI of the event.

The research team at a secondary site is responsible for following its site's guidelines for reporting applicable events to its site's IRB according to its own institutional guidelines. The research team at Washington University is responsible for reporting all applicable events to the FDA.

7.5 Reporting to Secondary Sites

The Washington University PI (or designee) will notify the research team at each secondary site of all reportable events that have occurred at other sites within **10 working days** of the occurrence of the event or notification of the PI of the event. This includes events that take place both at Washington University and at other secondary sites, if applicable.

7.6 Reporting to the FDA

The conduct of the study will comply with all FDA safety reporting requirements. **PLEASE NOTE THAT REPORTING REQUIREMENTS FOR THE FDA DIFFER FROM REPORTING REQUIREMENTS FOR HRPO/QASMC.** It is the responsibility of the Washington University principal investigator to report any unanticipated problem to the FDA as follows:

- Report any unexpected fatal or life-threatening adverse experiences (Section 7.1.4) associated with use of the drug by telephone or fax no later than **7 calendar days** after initial receipt of the information.
- Report any serious, unexpected adverse experiences (Section 7.1.2), as well as results from animal studies that suggest significant clinical risk within **15 calendar days** after initial receipt of this information.

All MedWatch forms will be sent by the investigator or investigator's team to the FDA at the following address or by fax:

Food and Drug Administration
Center for Drug Evaluation and Research
Division of Oncology Drug Products
5901-B Ammendale Rd.
Beltsville, MD 20705-1266
FAX: 1-800-FDA-0178

Secondary sites must submit a completed MedWatch form to the Washington University PI and research coordinator within **4 calendar days** (for fatal or life-threatening adverse experiences) or **11 calendar days** (for serious, unexpected adverse experiences). The Washington University PI will be responsible for submitting all MedWatch forms from secondary sites to the FDA within the timeframes specified above.

7.7 Reporting to Pfizer

Within 24 hours of first awareness of the event (immediately if the event is fatal or life-threatening), the PI or designee will report to Pfizer by facsimile any serious adverse drug experience (as defined in Section 7.9) that occurs during the SAE reporting period (as defined in Section 7.11) in a study subject assigned to receive PD 0332991. Such SAEs will be reported using MedWatch form and the Pfizer Reportable Event Fax Cover Sheet (Appendix G) should also be included. SAEs should be reported as soon as they are determined to meet the definition, even if complete information is not yet available.

Even though there may not be an associated SAE, exposure to PD 0332991 during pregnancy or lactation is reportable.

Secondary sites must submit a completed MedWatch form to the Washington University PI and research coordinator within the specified time frame. The Washington University PI will be responsible for submitting all MedWatch forms from secondary sites to Pfizer.

7.8 Timeframe for Reporting Required Events

Adverse events will be tracked for 30 days following the last day of study treatment.

8.0 PHARMACEUTICAL INFORMATION

8.1 Study Agent (PD 0332991)

8.1.1 PD 0332991 Description

Laboratory Code: PD 0332991-0000
Molecular Weight: 447.5
Molecular Formula: $C_{24}H_{29}H_7O_2$

Formulation: Capsules that use common compendial excipients (corn starch, microcrystalline cellulose, sodium starch glycolate, magnesium stearate (nonbovine)) will be used in clinical programs. The capsule shells are manufactured from gelatin NF.

8.1.2 Clinical Pharmacology

PD 0332991 is a highly selective inhibitor of Cdk4/cyclinD$_1$ kinase activity (IC_{50} = 11 nM; K_i = 2 nM). PD 0332991 has selectivity for Cdk4/6, with little or no activity against a large panel of 34 other protein kinases including other Cdks and a wide variety of tyrosine and serine/threonine kinases. Cdk6, another enzyme that also complexes with cyclin-D subunits, is also commonly expressed in mammalian cells and tumors. Cdk6 is highly homologous to Cdk4 and can perform the same function by phosphorylating Rb, thus potentially creating a redundant mechanism to promote cell cycle progression. Consequently, inhibition of both enzymes is necessary to ensure complete suppression of Rb phosphorylation and the greatest possible spectrum of antitumor activity. Results indicate that PD 0332991 inhibits Cdk6 with equivalent potency to Cdk4.

8.1.3 Pharmacokinetics and Drug Metabolism

To date pharmacokinetic data have been reported for four studies (A5481001, A5481002, A5481003 and A5481004). Final PK data are available from studies A5481001 and A5481002. Pharmacokinetic parameters are available from all 74

patients enrolled in Protocol A5481001 following a single-dose (Day 1 of Cycle 1), and from 51 patients following multiple-dose administration (Day 8 of Cycle 1) of daily doses ranging from 25 to 225 mg of PD 0332991 (Table 4.). On Day 1, all patients had detectable plasma concentrations of PD 0332991 at the first measured time point (1 hour) following oral administration. The exposure ($AUC_{(0-10)}$ and C_{max}) increased in a dose-proportional manner over the dose range of 25-225 mg QD following PD 0332991 administration on Days 1 and 8 of Cycle 1, although some variability (low to moderate) around these doses was observed particularly at the 150 mg QD dose level (Table 5).

Table 5
Summary of PD 0332991 Mean and Median Plasma PK Parameters by Dose (Day 1 and Day 8 Data Combined)

Treatment Description (QD)	Study Day	C_{max}[1] (ng/mL)	T_{max}[2] (hour)	$AUC_{(0-10)}$[1, 3] (ng.hour/mL)
25 mg	1 (n=3)	9.6 (63)	4.0 (4.0-4.0)	58 (51)
	8 (n=3)	15.9 (32)	4.0 (2.0-7.0)	119 (32)
50 mg	1 (n=3)	20.7 (3)	4.0 (4.0-4.3)	134 (5)
	8 (n=3)	35.7 (16)	4.1 (2.0-7.0)	274 (15)
75 mg	1 (n=7)	28.7 (24)	4.0 (4.0-10.0)	199 (20)
	8 (n=6)	58.6 (24)	4.0 (4.0-9.0)	492 (27)
100 mg	1 (n=6)	45.6 (45)	4.0 (2.0-10.0)	332 (34)
	8 (n=6)	71.2 (31)	5.5 (4.0-10.0)	513 (45)
125 mg	1 (n=22)	51.6 (43)	7.0 (2.0-24.4)	299 (44)
	8 (n=13)	86.2 (34)	4.0 (1.0-10.0)	724 (38)
150 mg	1 (n=7)	83.8 (17)	4.0 (4.0-9.8)	633 (9)
	8 (n=6)	161 (44)	7.0 (7.0-10.0)	1342 (42)
200 mg	1 (n=20)	80.8 (35)	5.7 (1.0-10.2)	525 (36)
	8 (n=8)	174 (17)	4.0 (2.0-7.0)	1395 (23)
225 mg	1 (n=6)	104 (58)	4.0 (4.0-7.0)	718 (55)
	8 (n=6)	186 (64)	4.5 (1.0-7.0)	1491 (64)

[1] C_{max} and $AUC_{(0-10)}$: mean (%CV)
[2] T_{max}: Median (Range)
[3] For $AUC_{(0-10)}$, the number of patients on Day 1 for the 100 mg, 125 mg, 150 mg and 200 mg groups were 5, 21, 5 and 19 respectively and on Day 8 for the 75 mg, 100 mg and 125 mg groups were 5, 4 and 12 respectively

Steady-state PK parameters are available (Table 6) for nine patients on Day 14 of Cycle 1 (receiving 200 mg SC 0332991 QD for 2 weeks) and four patients on Day 21 of Cycle 1 (receiving 125 mg QD for 3 weeks). PD 0332991 was absorbed with a median Tmax of ~4 hours. The mean PD 0332991 Vz/F was 3103 L, which is significantly greater than total body water (42 L), indicating that PD 0332991 extensively penetrates into peripheral tissues. PD 0332991 was eliminated slowly; the mean elimination half-life (t½) was 26.5 hours and the mean CL/F was 86.1 L/hour. PD 0332991 accumulated following repeated dosing with a median Rac of 2.4, which is consistent with the elimination half-life.

Table 6
Summary of the Steady-State Mean Plasma PK Parameters on Day 14 (200 mg) and Day 21 (125 mg) Following Oral Administration of PD 0332991 Dose Corrected to 125 mg Dose Level (N=13)

Treatment Description	C_{max}[1] (ng/mL)	T_{max}[2] (hour)	$AUC_{(0-24)}$[1] (ng.hour/mL)	$AUC_{(0-72)}$[1] (ng.hour/mL)	$t_{1/2}$[1] (hour)	CL/F[1] (L/hour)	V_z/F[1] (L)	R_{ac}[2,3]
Dose corrected 125 mg QD (n=13)	104 (48)	4.2 (2-9.8)	1863 (59)	3549 (71)	26.5 (26)	86.1 (50)	3103 (40)	2.4 (1.5-4.2)

[1] mean (%CV)
[2] Median (Range)
[3] For Rac, n=12 ($AUC_{(0-24)}$ was not estimable for Patient 10021099 on Cycle 1, Day 1 in the 200 mg group)
Note: Combined PK parameter data from Day 14 (200 mg) and Day 21 (125 mg) dose corrected to the 125 mg dose level.

Renal excretion of PD 0332991 was a minor route of elimination with ~1.7% of the drug excreted unchanged in urine over the 10-hour collection period in the 125 mg and 200 mg dose group, combined. The mean renal clearance (CLR) was 6.59 L/hour.

An exploratory evaluation of the circulating metabolites for PD 0332991 was conducted in plasma samples obtained from patients treated with PD 0332991 200 mg QD. Preliminary assessment of the pooled plasma samples on Day 14 of Cycle 1 indicated that the glucuronide conjugate of PD 0332991 and the lactam of PD 0332991 were the main metabolites present in plasma. Other metabolites observed were the glucuronide conjugates of hydroxylated PD 0332991 and the glucuronide conjugate of reduced PD 0332991.

The preliminary results from the recently performed food-effect study ("A5481021, a Phase 1, open-label 4 sequence 4 period crossover study of palbociclib (PD-0332991) in healthy volunteers to estimate the effect of food on the bioavailability of palbociclib") has provided evidence that when a single 125 mg dose of palbociclib was administered under fed conditions (including high fat or low fat meal given together with palbociclib, or moderate fat meal given 1 hour before and 2 hours after palbociclib) as a freebase formulation the palbociclib exposure levels were more uniform across the population than when taken in the fasting condition.
Drug-drug interaction between PD 0332991 and letrozole was evaluated during the Phase 1 portion of a breast cancer study (A5481003). The preliminary data indicate a lack of a potential for drug-drug interaction between PD 0332991 and letrozole when administered in combination.

8.1.4 Supplier(s)

Pfizer will supply the study agent. The study agent will be free of charge to the patient.

8.1.5 Dosage Form and Preparation

Medication will be provided in non-patient specific bottles containing either 125 mg, 100 mg, or 75 mg capsules. The patient number and the protocol number should be recorded on the bottle label in the spaces provided. Site personnel must ensure that patients clearly understand the directions for self-medication. Patients should be given a sufficient supply to last until their next study visit. Unused drug and/or empty bottles should be returned to the site at the next study visit. PD 0332991 is an agent that must be handled and administered with care. Patients should be instructed to keep their medication in the bottles provided and not transfer it to any other container. Due to possible unknown hazards associated with topical and environmental exposure to experimental agents, capsules must not be opened and/or emptied into any vehicle for oral ingestion; capsules must be swallowed intact.

8.1.6 Storage and Stability

Please store PD 0332991 capsules according to storage conditions on the label. Medication should be kept in a secured locked area at the study site in accordance with applicable regulatory requirements. Returned medication should be stored separately from medication that needs to be dispensed.

To ensure adequate records, PD 0332991 capsules will be accounted for as instructed by Pfizer. Unless otherwise authorized by Pfizer, at the end of the clinical trial all drug supplies unallocated or unused by the subjects must be returned to Pfizer or its designee. All containers of PD 0332991 that were sent to the investigator throughout the study must be returned to the sponsor or designee, whether they are used or unused, and whether they are empty or contain capsules.

8.1.7 Administration

Patients should be instructed to swallow PD 0332991 capsules whole and not to chew them prior to swallowing. No capsule should be ingested if it is broken, cracked, or otherwise not intact. Patients should take PD 0332991 with food and should be encouraged to take their dose at approximately the same time each day.

8.1.8 Special Handling Instructions

Females of childbearing potential should not handle or administer the study agent unless they are wearing gloves.

8.1.9 Pregnancy

Fertility and teratology studies with PD 0332991 have not been conducted; therefore, safety for pregnant women of childbearing capacity and for the fetus cannot be implied from the existing data. If the drug is used during pregnancy, or if the patient becomes pregnant while receiving this drug, the patient should be apprised of the potential hazard to the fetus.

PD 0332991 caused testicular degeneration in rats and dogs. The incidence and severity was dose related and correlated with decreases in testicular weight in the rat. Testicular degeneration was not reversed after cessation of treatment and progressed in severity in both species. Testicular degeneration produced by PD 0332991 is consistent with Cdk inhibition and alterations in cell cycle kinetics.

Women of childbearing potential must have a negative pregnancy test prior to treatment with PD 0332991. Female patients must be surgically sterile or be postmenopausal, or must agree to use effective contraceptive during the period of the trial and for at least 90 days after completion of treatment. The decision of effective contraception will be based on the judgment of the principal investigator or a designated associate.

8.1.10 QT Interval

The patients enrolled in clinical studies should be closely monitored for potential cardiovascular symptoms. Appropriate monitoring should include clinical examinations, vital signs, routine ECGs, and AEs monitoring. In case of QTc prolongation, concomitant conditions such as electrolyte unbalances or use of medications affecting the QT interval should be ruled out or corrected. In case of clinically significant toxicities, PD 0332991 administration should be interrupted and the dose reduced as indicated in clinical protocols.

In Study A5481001 using QTcF, 46 of 73 patients had a maximum increase from baseline of <30 msec and no patient had a maximum on treatment value of ≥500 msec. Notably, one female patient who had received PD 0332991 at 75 mg QD on Schedule 3/1, had a maximum QTcF increase of 67 msec from baseline to Cycle 1. Additionally, QTcF increases ranging from 39 to 51 msec compared to baseline persisted throughout her ECG collection period of 5 subsequent cycles. After 7 cycles, the dose was increased to 100 mg QD. The patient remained on treatment for a total of 39 cycles with no cardiac related adverse events. QT data analysis for study A5481002 indicated no clinically significant mean changes with ECGs. Using Fridericia's correction in the A5481002 study, all 17 subjects in the analysis had a maximum increase from baseline of <30 msec and a maximum post-baseline value for QTc of <500 msec.

8.2 Anastrozole

8.2.1 Anastrozole Description

Anastrozole is a nonsteroidal aromatase inhibitor.

Chemical Name or Amino Acid Sequence: 1,3-Benzenediacetonitrile, a, a, a', a'-tetramethyl-5-(1H-1,2,4-triazol-1-ylmethyl)
Other Names: Arimidex
Classification: Aromatase inhibitor
Molecular Formula: $C_{17}H_{19}N_5$ **M.W.:** 293.4
Approximate Solubility: Anastrozole has moderate aqueous solubility (0.5 mg/mL at 25°C); solubility is independent of pH in the physiological range. Anastrozole is freely soluble in methanol, acetone, ethanol, and tetrahydrofuran, and very soluble in acetonitrile.

8.2.2 Mode of Action

Anastrozole is a potent and selective non-steroidal aromatase inhibitor. It significantly lowers serum estradiol concentrations and has no detectable effect on formation of adrenal corticosteroids or aldosterone [AstraZeneca, Package Insert].

8.2.3 How Supplied

Anastrozole tablets are manufactured by AstraZeneca. Anastrozole tablets for oral administration contain 1 mg of anastrozole.

Anastrozole is commercially available and will be billed to the patient or her insurance.

8.2.4 Dosage Form and Preparation

Anastrozole is an off-white powder. Each tablet contains as inactive ingredients: lactose, magnesium stearate hydroxypropylmethylcellulose, polyethylene glycol, povidone, sodium starch glycolate, and titanium dioxide [AstraZeneca, Package Insert].

8.2.5 Storage

Store at controlled room temperature at 20-25°C.

8.2.6 Method of Administration

Patients should be instructed to take anastrozole tablets by mouth with or without food.

8.2.7 Potential Drug Interactions

Anastrozole is generally safe to administer with other medicines. However, concomitant use of agents and herbal products that alter ER function are specifically not allowed.

For further information, please refer to the FDA-approved package insert for anastrozole.

8.3 Goserelin

8.3.1 Goserelin Description

Synthetic decapeptide analogue of GnRH.

Chemical Name or Amino Acid Sequence: [D-Ser(Bu^t)^6,Azgly^{10}]. Its chemical structure is pyro-Glu-His-Trp-Ser-Tyr-D-Ser(Bu^t)-Leu-Arg-Pro-Azgly-NH_2 acetate.
Other Names: Zoladex
Classification: GnRH agonist
Molecular Formula: [$C_{59}H_{84}N_{18}O_{14}$•$(C_2H_4O_2)_x$ where x = 1 to 2.4]
M.W.: 1269
Approximate Solubility: Goserelin is freely soluble in glacial acetic acid. It is soluble in water, 0.1M hydrochloric acid, 0.1M sodium hydroxide, dimethylformamide and dimethyl sulfoxide. Goserelin acetate is practically insoluble in acetone, chloroform and ether [AstraZeneca, Package Insert].

8.3.2 Mode of Action

Goserelin has actions similar to those of naturally occurring GnRH (also known as LHRH). Normally, GnRH is released in a pulsatile manner to maintain levels of gonadotropins. Goserelin, in contrast, is continuously administered, which leads to down-regulation of the GnRH receptor on the pituitary gland and ultimately decreased production of FSH and LH.

8.3.3 How Supplied

Goserelin is supplied as a sterile, biodegradable product containing goserelin acetate equivalent to 3.6 mg of goserelin. Goserelin is designed for subcutaneous injection with continuous release over a 28-day period. Goserelin acetate is dispersed in a matrix of D,L-lactic and glycolic acids copolymer (13.3-14.3 mg/dose) containing less than 2.5% acetic acid and up to 12% goserelin-related substances and presented as a sterile, white to cream colored 1-mm diameter cylinder, preloaded in a special single use syringe with a 16-gauge x 36 +/- 0.5 mm siliconized needle with protective needle sleeve (SafeSystem™ Syringe) in a

sealed, light and moisture proof, aluminum foil laminate pouch containing a desiccant capsule. Studies of the D,L-lactic and glycolic acids copolymer have indicated that it is completely biodegradable and has no demonstrable antigenic potential [AstraZeneca, Package Insert].

Goserelin is commercially available and will be billed to the patient or her insurance.

8.3.4 Storage

Store at controlled room temperature (do not exceed 25°C).

8.3.5 Method of Administration

Goserelin should be administered subcutaneously every 28 days into the anterior abdominal wall below the navel line using an aseptic technique under the supervision of a physician.

8.3.6 Potential Drug Interactions

Goserelin is generally safe to administer with other medicines.

For further information, please refer to the FDA-approved package insert for goserelin.

9.0 CORRELATIVE STUDIES

9.1 Sample Collection, Processing, and Shipment

9.1.1 Sample Collection/Shipment Kit for Neoadjuvant Tumor and Blood Collection

The kit is a two-chamber kit in which it is possible to send both frozen and ambient specimens. The kit is stocked with all the necessary items needed to draw blood and obtain tissue specimens. It also contains the necessary materials used to process and prepare specimens for shipment in accordance to IATA regulations. Finally the kit contains all required documentation and labels required to return the kit to the Washington University Tissue Procurement Core Facility (address listed below).

It is advised that the baseline samples are harvested during ultrasound guided clip placement to optimize tissue accrual.

Sample collection/shipment kits are available for institutions by sending the request to Washington University Tissue Procurement Facility below:

Wash U Alliance/ACOSOG-CBS/TPS
425 S. Euclid Ave, Room 5120
St. Louis, MO 63110-1005
Phone: 314-454-7615
Email: tbank@wudosis.wustl.edu

9.1.2 Sample Collection and Schedule in the Neoadjuvant Setting (PIK3CA Mutant and WT Cohorts)

All sample collection is mandatory.

Correlative Study	Blood / Tumor	Type of Tube	Volume to Collect	Time point	Process at Site?	Temperature Conditions for Storage / Shipping
Tumor for PIK3CA testing	Core biopsies	2 cores in OCT<br>2 cores in 10% formalin	14-G core needle	Pre-anastrozole	No	Cores in OCT should be immediately frozen; cores in formalin should be stored / shipped at ambient temperature
Tumor for research♣	Core biopsies	2 cores in OCT<br>2 cores in 10% formalin	14-G core needle	Pre-PD0332991, C1D15, Surgery*	No	Cores in OCT should be immediately frozen; cores in formalin should be stored / shipped at ambient temperature
Plasma for research**	Whole blood	EDTA (purple)	10 mL	Pre-anastrozole†, Pre-PD0332991, C1D15, Surgery	Yes	-80ºC
Plasma for PK studies	Whole blood	K2EDTA (purple)	10 mL	Pre-PD0332991#, C1D15#	Yes	-80ºC (or -20ºC)
Serum for research**	Whole blood	Clot-tube (red)	10 mL	Pre-anastrozole†, Pre-PD0332991, C1D15, Surgery	Yes	-80ºC
Germline DNA for research	Whole blood	EDTA (purple)	10 mL	Pre-anastrozole†, Pre-PD0332991	No	Ambient
Whole blood for plasma circulating DNA	Whole blood	Cell-free DNA BCT	10 mL	Pre-anastrozole, Pre-PD0332991, C1D15, Surgery	No	Room temperature, same-day shipment (avoid Friday blood draw)

Pre-anastrozole: C0D1; Pre-PD0332991: C1D1
* Additional surgical tumor specimens are required; please refer to instruction 'g' below
** Blood could be collected the same time as the PK samples (either prior to or after drug administration) on Pre-PD0332991 and C1D15.
† For patients who pre-registered to this protocol only.
# Prior to and 90 minutes following drug administration (see Section 9.4.7).
♣ Tumor collection prior to PD0332991 is not required for the endocrine resistant cohort if the patient had undergone research tumor collection as part of the tumor biopsy for the Ki67 analysis prior to registration to this trial.

Instructions for sample collection and processing:

Note that sample collection/shipment kits are available for the study.

a: For biopsies at each time point: 4 cores (14 G core needle) will be taken at each time point, with 2 in 10% formalin and 2 freshly frozen in OCT. Care needs to be taken to reduce the ischemic time to as much as possible to less than 30 min.

b: A repeat biopsy may be needed in consented patients if the first biopsy did not yield enough tumor cells for Ki67.

c: Samples can be obtained at the time of surgery. Care needs to be taken to reduce the ischemic time to as much as possible to less than 30 minutes.

d: **Plasma processing:** Upon collection of the blood PK samples, keep the samples on wet ice at all times prior to processing to plasma. The blood samples must be processed to plasma and placed in the freezer at -20°C within one hour of collection. To process the blood samples to plasma, centrifuge the blood samples at approximately 4°C at 1700xg for approximately 10 minutes. Using a separate pipette for each time point, transfer the plasma samples into pre-labeled amber polypropylene cryovials and store at approximately -20°C until shipment. As much as practical, keep the blood and plasma samples away from direct sunlight and unfiltered lab light. Ship the samples on dry ice to the analytical labs.

e: **Germ line DNA processing:** The EDTA tube should be mixed several times and labeled with the patient's study number, date of birth, and collection date and time. Whole blood specimens are shipped in the specimen kit and must be received by the Washington University Tissue Procurement Facility **within 48 hours of the time of collection**. **Do not freeze whole blood.**

f: **Whole blood for plasma circulating DNA:** The cell free DNA collection BCT tube (provided with the kits) should be mixed several times and labeled with the patient's study number, date of birth, and collection date and time. Whole blood specimens are shipped in the specimen kit and must be received by the Washington University Tissue Procurement Facility within 48 hours of the time of collection. Do not freeze whole blood.

g: Slide submission for the surgical specimen is required for all patients for research, in addition to the biopsies described. The request of these additional surgical materials is due to the concern that the biopsy samples from the surgical specimens may not contain sufficient tumor for correlative studies of residual tumors.

h: When the local pathological analysis of tumor samples from the definitive surgical procedure is complete, 10 unstained *Superfrost Plus* slides and the corresponding pathology report should be submitted to the Washington University Tissue Procurement Facility. Alternatively, a tissue block containing the residual cancer may be submitted and stored at the Washington University Tissue Procurement Facility unless a request is received to return the block (at which point 10 sections and 4 1-mm TMA cores will be taken, and then the block will be returned). Further fixed material from the diagnostic biopsy or surgical specimen may be requested by the Washington University Tissue Procurement Facility at a later date to complete sample pairs if insufficient tumor is present in the specimens that were previously provided. Samples should be shipped as soon as possible after surgery.

9.1.3 Sample Collection and Schedule in the Neoadjuvant Setting (Endocrine Resistant Cohort)

All sample collection is mandatory.

Correlative Study	Blood / Tumor	Type of Tube	Volume to Collect	Time point	Process at Site?	Temperature Conditions for Storage / Shipping
Tumor for research♣	Core biopsies	2 cores in OCT<br>2 cores in 10% formalin	14-G core needle	Pre-PD0332991, C1D15, Surgery*	No	Cores in OCT should be immediately frozen; cores in formalin should be stored / shipped at ambient temperature
Plasma for research	Whole blood	EDTA (purple)	10 mL	Pre-PD0332991, C1D15, Surgery	Yes	-80ºC
Serum for research	Whole blood	Clot-tube (red)	10 mL	Pre-PD0332991, C1D15, Surgery	Yes	-80ºC
Germline DNA for research	Whole blood	EDTA (purple)	10 mL	Pre-PD0332991	No	Ambient, same-day shipment (avoid Friday blood draw)
Whole blood for plasma circulating DNA	Whole blood	Cell-free DNA BCT	10 mL x2	Pre-PD0332991, C1D15, Surgery	No	Ambient, same-day shipment (avoid Friday blood draw)

Pre-PD0332991: C1D1

♣ Tumor collection prior to PD0332991 is not required for the endocrine resistant cohort if the patient had undergone research tumor collection as part of the tumor biopsy for the Ki67 analysis prior to registration to this trial. Consent to research of archival tumor specimens collected prior to and during this trial is required for all patients.

* Additional surgical tumor specimens are required; please refer to instruction 'g' below

Instructions for sample collection and processing:

Note that sample collection/shipment kits are available for the study.

a: For biopsies at each time point: 4 cores (14 G core needle) will be taken at each time point, with 2 in 10% formalin and 2 freshly frozen in OCT. Care needs to be taken to reduce the ischemic time to as much as possible to less than 30 min.

b: A repeat biopsy may be needed in consented patients if the first biopsy did not yield enough tumor cells for Ki67.

c: Samples can be obtained at the time of surgery. Care needs to be taken to reduce the ischemic time to as much as possible to less than 30 minutes.

d: **Serum and plasma processing:** Keep the samples on wet ice at all times prior to processing to plasma. The blood samples must be processed to plasma and placed in the freezer at -20°C within one hour of collection. To process the blood samples to plasma, centrifuge the blood samples at approximately 4°C at 1700xg for approximately 10 minutes. Using a separate pipette for each time point, transfer the plasma samples into pre-labeled amber polypropylene cryovials and store at approximately -20°C until shipment. As much as practical, keep the blood and plasma samples away from direct sunlight and unfiltered lab light. Ship the samples on dry ice to the analytical labs.

e: **Germ line DNA processing:** The EDTA tube should be mixed several times and labeled with the patient's study number, date of birth, and collection date and time. Whole blood specimens are shipped in the specimen kit and must be received by the Washington University Tissue Procurement Facility **within 48 hours of the time of collection**. **Do not freeze whole blood.**

f: **Whole blood for plasma circulating DNA:** The cell free DNA collection BCT tube (provided with the kits) should be mixed several times and labeled with the patient's

study number, date of birth, and collection date and time. Whole blood specimens are shipped in the specimen kit and must be received by the Washington University Tissue Procurement Facility within 48 hours of the time of collection. Do not freeze whole blood.

g: Slide submission for the surgical specimen is required for all patients for research, in addition to the biopsies described. The request of these additional surgical materials is due to the concern that the biopsy samples from the surgical specimens may not contain sufficient tumor for correlative studies of residual tumors.

h: When the local pathological analysis of tumor samples from the definitive surgical procedure is complete, 10 unstained *Superfrost Plus* slides and the corresponding pathology report should be submitted to the Washington University Tissue Procurement Facility. Alternatively, a tissue block containing the residual cancer may be submitted and stored at the Washington University Tissue Procurement Facility unless a request is received to return the block (at which point 10 sections and 4 1-mm TMA cores will be taken, and then the block will be returned). Further fixed material from the diagnostic biopsy or surgical specimen may be requested by the Washington University Tissue Procurement Facility at a later date to complete sample pairs if insufficient tumor is present in the specimens that were previously provided. Samples should be shipped as soon as possible after surgery.

9.1.4 Sample Collection and Schedule during Follow-up

<u>Patients who received adjuvant PD 0332991</u>

Research blood includes the collection of plasma, serum, and whole blood for cfDNA. Research blood will be collected on Day 1 +/- 3 days of adjuvant Cycles 1 and 12 and at the end of treatment (either end of Cycle 23 or end of the last cycle received if the patient ends treatment early). Additional research draws will take place annually for 3 years following the last dose of PD 0332991 (+/- 8 weeks).

<u>All other patients</u>

Research blood includes the collection of plasma, serum, and whole blood for cfDNA. Research blood will be collected 1 year post surgery, then annually for a total of 5 years following surgery or until recurrence (whichever comes first).

Blood for all patients will be collected as follows:

- 10 mL in an EDTA (purple top) tube for plasma to be stored at -80°C; to process the blood samples to plasma, centrifuge the blood samples at approximately 4°C at 1700xg for approximately 10 minutes.
- 10 mL in a clot (red top) tube for serum to be stored at -80°C
- 10 mL each in 2 cell-free DNA BCT tubes to be stored at room temperature and shipped same day to the Washington University Tissue Procurement Core Facility; the cell free DNA collection BCT tube (provided) should be mixed several times and labeled with the patient's study number, date of birth, and collection date and time. Whole blood specimens are shipped and must be received by the Washington University Tissue Procurement Facility within 48 hours of the time of collection.

9.1.5 Shipment of Samples

All samples should be labeled with institutional surgical pathology number (tumor samples), study number, patient ID number patient initials, sample collection date and time and be accompanied by the completed specimen submission forms (Appendix F/K/L (as appropriate)).

All samples should be shipped to the Wash U Tissue Procurement Core Facility.

Specimens may be sent to the Wash U Tissue Procurement Facility on Monday through Thursday for next day delivery. **The Bank cannot receive specimens on Saturdays, Sundays, or holidays. Do not send specimens on Friday, Saturday, or the day before a holiday**.

The institution is expected to pay the cost of mailing specimens and will be reimbursed through capitation fees set for each individual study.

Arrange for Federal Express pick-up through your usual institutional procedure. Ship specimens to the address below:

Wash U Alliance/ACOSOG-CBS/TPS
425 S. Euclid Ave, Room 5120
St. Louis, MO 63110-1005
Phone: (314) 454-7615
E-mail: tbank@wudosis.wustl.edu

On the day that specimens are sent to the specimen bank, please contact the bank by phone, fax, or e-mail to notify what is being sent and when the shipment is expected to arrive.

9.2 Real Time Integral Biomarker Studies

Note that the PIK3CA (Gene ID: 5290) hot spot sequencing assay that covers exons 1, 4, 7, 9 and 20, is done while the patient was pre-registered to the NCI 9170 trial or through this trial. Briefly, sequencing of these 5 exons and exon-intron splice junctions should be performed using the established protocol at the CLIA certified GPS@WU, a CAP-accredited (#27556-03) and CLIA-licensed (#26D0698685) laboratory environment under the unified supervision by faculty in the Departments of Genetics and Pathology and Immunology at Washington University.

Tumor biopsy will be sectioned and H&E stained for evaluation of tumor cellularity by a pathologist and processed to tumor DNA and RAN extraction at Washiongton University once it arrives at the Alliance Central Specimen Bank. The first of the two formalin fixed and paraffin embedded (FFPE) tumor cores will be sectioned and stained by H&E to assess for tumor cellularity. If the total normal of tumor nuclei is greater than 75% in the biopsy, serial sections will be immediately prepared for DNA and RNA extraction. If

tumor cellularity is less than 75%, the second frozen core will be evaluated. In cases where both FFPE tumor biopsies contain less than 75% neoplastic cellularity, macro- or micro-dissection of tumor rich area will be performed if there is sufficient tumor. If neither baseline sampling core contains sufficient tumor cellularity, the frozen tissue will be examined. If needed a second biopsy may be required if the patient consents.

High quality DNA sequence reads that are concordant on both strands will be analyzed from each amplicon. Variant calls (as compared to reference sequence) will be reported as a qualitative / non-ordered categorical result. The position (nucleotide, exon, amino acid, and protein domain), sequence variant, and predicted change (if any) in corresponding amino acid sequence will be reported. If a non-synonymous variant identified has never been reported to be a somatic mutation, germ-line DNA will be sequenced from the same patient to determine whether this is a somatic mutation or a polymorphism. The result will be further classified as: 1) Previously described somatic mutation; 2) Novel somatic mutation, not a germ-line polymorphism. Results will be emailed to the study coordinator at the participating site. Contact cbumb@ wustl.edu for questions related to the sequencing result.

Patients with or without a somatic mutation in PIK3CA hotspot region are eligible for the trial.

9.3 Tumor Ki67 Assessment on Cycle 1 Day 1 (Pre-PD 0332991) and Cycle 1 Day 15

9.3.1 Rationale

If Ki67 is over 10% on Cycle 1 Day 15, patient will be taken off the study drug therapy and recommended to either immediate surgery or neoadjuvant chemotherapy. If Ki67 is 10% or less, the patient will continue therapy for a total of 5 cycles of combination PD 0332991 and endocrine therapy.

9.3.2 Tissue Processing and Ki67 Analysis

Upon receipt of the specimen, the 2 fixed biopsy specimens (A & B) at each time point will be further processed for tumor Ki67 analysis at the CLIA certified Anatomic and Molecular Pathology Core Labs at Barnes Jewish Hospital at St. Louis (CLIA number 26D2013203). After embedding in paraffin, one section from the tumor block will be stained with H&E to assess biopsy adequacy. Another section from the block will be incubated with antibody against Ki67 (clone 30-9) and then assayed using the Ventana Benchmark platform. The block and any remaining sections will be returned to the Tissue Procurement Core Facility at the completion of testing. Both cores will be reviewed and will be taken into account for the scoring. Ki67 scoring is reported as a quantitative/continuously distributed value.

9.3.3 Ki67 Scoring and Reporting

Ki67 scoring will be performed using a standard SOP established at the CLIA laboratory, with the result expressed as immunoreactive over total numbers of cells. The Ki67 data for C1D15 biopsy is available real time to the treating physician and the patients. Patients will be continued on protocol therapy while awaiting analysis results. Results of tumor Ki67 will be reported within 10 working days upon receipt of the samples. Wash U Path Coordinator will email and fax the results to both the CRA listed on Specimen Submission CRF that is sent with the tissue samples and Wash U CRA. Contact cbumb@wustl.edu for questions related to the Ki67 result.

Note: If the biopsy yielded no tumor cells, the patient may continue on study drug therapy or proceed with a second biopsy for Ki67 determination.

9.4 Laboratory Correlative Studies

Laboratory correlative studies are performed on leftover tumor specimens following the integral biomarker analysis (Section 9.2) from baseline, on Cycle 1 Day 1 (pre-PD 0332991), Cycle 1 Day 15, and on surgical specimens.

9.4.1 To assess tumor cell apoptosis on serial tumor specimens

Methods: FFPE tumor blocks obtained from biopsies at baseline and post therapy will be sectioned at 5 micron sections for immunohistochemistry analysis of cleaved caspase 3 or TUNEL staining with established method at the Study PI and Co-PI's laboratory. Apoptotic index will be calculated as the percentage of tumor cells staining positive for apoptotic markers (either cleaved caspase 3 or TUNEL staining).

9.4.2 To assess Ki67 level on serial tumor biopsies

Methods: Ki67 at baseline, Cycle 1 Day 1 (pre-PD 0332991), Cycle 1 Day 15, and surgery will be performed at the CLIA certified Anatomic and Molecular Pathology (AMP) facility at Washington University on FFPE sections of serially collected tumor biopsies. Ki67 index will be calculated as the percentage of tumor cells stained positive at each time point.

9.4.3 To assess effect of PD 0332991 on markers of tumor cell senescence

Methods: Candidate proteins important for senescence including p16, cyclin D1, activated mTOR (such as pS6), IL6, senescence associated beta-galactosidase, FOXM1 phosphorylation, will be examined by IHC.

9.4.4 To examine the pharmacodynamic effect of PD 0332991 in combination with endocrine therapy on Cdk4/6 Rb signaling activities

Methods: The pharmacodynamic effect of PD 0332991 in combination with anastrozole on Cdk4/6-Rb signaling will be assessed by phosphoroproteomics and immunohistochemistry analysis of pRB and other candidate molecules on serial tumor biopsies.

9.4.5 Assessment of serum estradiol level before and following PD 0332991

Sensitive estradiol level in premenopausal women will be tested in the clinical laboratory at the end of 4 weeks of anastrozole (or in combination with goserelin) (at baseline), Cycle 1 Day 15, and surgery.

9.4.6 Explore potential markers of sensitivity and resistance

In preclinical studies, RB proficient cell lines with low p16 expression were found to be most sensitive to PD 0332991 in ovarian cancer. In addition, copy number variations of CDKN2A, RB, CCNE1, CCND1 were associated with response[102]. Therefore, tumor specimens collected on Cycle 1 day 1 will be analyzed for the protein expression of these genes by immunohistochemistry. Gene copy number changes will be assessed by aCGH method. These studies will be performed in the study PI and Co-PI's laboratory. Other studies include hypothesis generating exploratory DNA and RNA studies as well as proteomic analysis.

9.4.7 Pharmacokinetics

Ten mL of blood will be drawn into each of 2 K_2EDTA tubes at the following time points to evaluate the concentrations of PD 0332991 and the effect of PD 0332991 on the concentrations of anastrozole:

- Prior to initiation of PD 0332991 but after patients have taken anastrozole alone or in combination with goserelin for at least 2 weeks (this could be done on Cycle 1 Day 1):
    - pre-dose of anastrozole
    - 90 minutes following this same dose of anastrozole
- Cycle 1 Day 15 (may take place up to Day 21 if samples are missed)
    - prior to both anastrozole and PD 0332991
    - 90 minutes following administration of both drugs

Process blood to plasma within 30-45 minutes by centrifuging in a refrigerated centrifuge (1700 x g for about 10 minutes at 4ºC) within 1 hour of collection. The collection tube containing PD 0332991 should be covered with aluminum foil to protect from light. Transfer plasma into pre-labeled amber polypropylene storage cryovials and store at -20ºC. Ship the samples on dry ice to the following address:

Wash U Alliance/ACOSOG-CBS/TPS
425 S. Euclid Ave., Room 5120

St. Louis, MO 63110-1005
Phone: (314) 454-7615 // E-mail: tbank@wudosis.wustl.edu

At the completion of the 1st stage and the 2nd stage of the study, batched samples will be sent by Washington University CRA to the following addresses for analysis:

For PD 0332991 analysis:

Maria Edwards c/o PPD
2244 Dabney Road
Richmond, VA 23230
Phone: (804) 977-8430 // E-mail: maria.edwards@ppdi.com

For anastrozole analysis:

Robert Twieg c/o Covance Laboratories, Inc.
8211 SciCor Drive, Suite B
Indianapolis, IN 46214
Phone: (317) 715-3964 // E-mail: Robert.Twieg@covance.com

10.0 STUDY CALENDARS

10.1 Pre-Registration and Cycle 0 Calendar

This applies only to the PIK3CA WT and Mutant Cohorts (closed as of Amendment #7).

	≤ 14 days prior to pre-registration	Pre-Registration	≤ 14 days Pre-Anastrozole	Cycle 0 Day 1 [A] (≤ 7 days post pre-registration)
**TREATMENT**				
Anastrozole				Days 1-28[B]
Goserelin[C]				X
**CLINICAL ASSESSMENT**				
H&P, PS	X		X	
Clinical measurements of breast lesions[D]	X		X	
Adverse event assessment				
CBC and differential	X		X	
Serum chemistry[E]	X		X	
Serum pregnancy test[C]	X			
Serum estradiol	X			
EKG (12 lead)	X			
**RADIOLOGY**				
Mammogram and ultrasound of breast and axillary masses[F]	X[F]			
**SAMPLE COLLECTION**				
Tumor biopsies [R]			X[G]	
Research blood samples [R]			X[H]	

A. Cycle 0 Day 1 is to start ≤ 7 days post pre-registration. Tumor biopsy and blood collections must be done prior to the start of anastrozole.

B. All effort is to be made to keep Cycle 0 length at 28 days, but up to 42 days of Cycle 0 therapy is allowed prior to the start of Cycle 1 Day 1 due to delays in PIK3CA sequencing result or scheduling difficulties. In this situation, goserelin should be administered every 28 days throughout the study regardless of cycle days.

C. In premenopausal women only

D. Using a standard cm calibrated caliper, tape or ruler, the longest axis and the perpendicular axis of the tumor are to be measured and recorded in metric notation. In patients with synchronous lesions, it is recommended that both lesions be measured and biopsied the same if they are both >1cm with at least one dimension.

E. Serum chemistry is albumin, alkaline phosphatase, total bilirubin, bicarbonate, BUN, calcium, chloride, creatinine, glucose, potassium, total protein, SGOT/AST, SGPT/ALT, sodium

F. Mammogram and ultrasound of the diseased breast must be completed within 42 days of pre-registration. Imaging studies must include bidimensional breast tumor measurements, although retrospective measurement of the imaging study is allowed after patient pre-registered on the study.

G. Pre-anastrozole core biopsies for correlative studies are required prior to the initiation of anastrozole therapy. Samples obtained at the time of diagnosis before registration to this trial (for other studies) may be submitted if they were collected according to the tissue acquisition instructions in Section 9. It is advised that the baseline samples are harvested during ultrasound guided clip placement to optimize tissue accrual.

H. For patients pre-registered to this trial only.

R. Research funded

10.2 Neoadjuvant Study Treatment Calendar (ALL Cohorts)

	≤ 14 days prior to registration	C1D1[A]	C1D15 ±1 day	C2-5 D1±3 days	3 (+/-1) days prior to surgery	Off Study - Progression	Surgery[B]	Post-surgery[C]
**TREATMENT**								
Anastrozole[E]		Daily until the day of surgery (if no progression)						
Goserelin[E]		Every 28 days (if no progression)						
PD 0332991[F]		3 weeks on and 1 week off [G,T]				D/C		
**CLINICAL ASSESSMENT**								
H&P, PS	X	X		X		X		X
Clinical meas. of breast lesions[J]	X	X		X		X		Exam
Adverse event assessment	X	X		X		X		X
CBC + differential	X			X	X[Y]			X
Serum chemistry[K]	X			X				X
Serum pregnancy test[L]		X			X	X		
Drug compliance assessment				X				
EKG (lead II) [N]	X						X	
**RADIOLOGY**								
Mammogram and US of breast and axillary masses[O]	X[H]			X[H]		X		
**SAMPLE COLLECTION**								
Tumor biopsies [R]		X[W]	X[P]	X[Q]		X[S]	X	
Research blood [R]		X[U]	X[P,V]			X[S]	X	

A. C1D1 office visit, labs, EKG,as well as pre-PD 0332991tumor biopsies may be done within 14 days prior to C1D1.
B. Please refer to section 5.1. Surgery occurs between Cycle 5 day 11 and day 13 in patients who received Cycle 5 treatment. In patients who did not receive Cycle 5 treatment, surgery occurs 3 to 5 weeks following the last dose of PD 0332991.
C. 30-60 days post-surgery, then yearly to document recurrence and survival for 5 years.
E. Anastrozole (and goserelin if premenopausal) should have been started at least 28 days prior to C1D1 **(only applies to PIK3CA Mutant and Wild Type cohorts).
F. Patients will keep a pill diary (Appendix D).
G. If C1D15 Ki67 is > 10%, patient will discontinue PD 0332991 and be observed for AEs 30-60 days following the last dose, then followed per protocol. Further treatment is at the discretion of the treating physician.
H. Prior to start of PD 0332991 (within 12 weeks prior to registration) and at the end of cycle 4 before surgery.
J. 2D measurements of the breast lesion should be performed. Using a standard cm calibrated caliper, tape or ruler, the longest axis and the perpendicular axis of the tumor are to be measured and recorded in metric notation. In patients with synchronous lesions, it is recommended that both lesions be measured and biopsied the same if they are both >1cm with at least one dimension; otherwise, the dominant mass is followed clinically and by Ki67
K. Serum chemistry is albumin, alkaline phosphatase, total bilirubin, bicarbonate, BUN, calcium, chloride, creatinine, glucose, potassium, total protein, SGOT/AST, SGPT/ALT, sodium
L. In premenopausal women only
N. EKG will be performed at baseline, pre-surgery, and as needed. Surgery may be delayed if QTc prolongation has occurred.
O. Mammogram and ultrasound of the diseased breast must be completed within 12 weeks prior to registration. Imaging studies must include bidimensional breast tumor measurements, but retrospective measurement is allowed in the study.
P. C1D15 biopsy and blood draw may be done up to C1D20 for scheduling convenience, although C1D15 is strongly preferred.
Q. Optional biopsy on C2D15, C3D15, or C4D15 in patients with indeterminate Ki67 on C1D15 biopsy or who missed the C1D15 biopsy.
R. Research funded. Research blood samples are mandatory except where indicated.
S. Optional
T. If counts recover within 3 weeks of the end of C4, re-start PD 0332991 for 10 to 12 doses (C5) (last dose to be the day before surgery).
U. PKs to be drawn on a day prior to initiation of PD 0332991 (may be C1D1) pre-anastrozole and again 90 minutes post-anastrozole. Other research blood include serum, plasma, whole blood for germline DNA and whole blood for circulating tumor DNA is also collected on C1D1. Details are described in Section 9.1.2. PK samples are not required in the endocrine resistant cohort.
V. PKs to be drawn pre-dose of both drugs and again 90 minutes post-dose of both drugs. PK samples are not required in the endocrine resistant cohort.
W. Not required in endocrine resistant cohort if a research biopsy was performed prior to registration or an archival tumor block is available

Y. Only required in patients who are in Cycle 5.

10.3 Post Surgery Treatment Calendar

Patients who had a Ki67<10% on the neoadjuvant C1D15 tumor biopsy in the endocrine resistant cohort (before or after activation of Amendment #7) and the 26 patients who enrolled initially to the main trial (PIK3CA WT or mutant cohort) who required PD 0332991 to achieve complete cell cycle arrest have the option of restarting PD 0332991 in combination with anastrozole for adjuvant treatment (23 cycles after surgery). These patients will need to be consented and screened for the adjuvant portion of the study, and may only begin treatment with adjuvant PD 0332991 if they meet the eligibility criteria in Section 3.4 and if they have provided informed consent.

PD 0332991 should be re-started at least 4 weeks after the completion of chemotherapy and radiation therapy if these treatments were planned. Anastrozole may be started earlier while the patient receives radiation therapy if desired. After completion of PD 0332991, patients should continue standard of care hormonal therapy (for example anastrozole) to complete at least 5 years of therapy. Further therapy after that is at the discretion of treating physician.

	Screening	Adjuvant Cycles 1-2		Adjuvant Cycles 3 to 23	End of C23 or early termination	Follow up Yearly post the last dose of palbociclib to complete 5 years of follow up post surgery or recurrence[F]
Day of cycle	-30 to 0	D1 +/- 3 days	D14 +/- 3 days	D1 +/- 7 days (C3, 6, 9, 12, 15, 18, 21)	4 weeks	+/- 8 weeks
**TREATMENT**						
Endocrine Therapy [A]		Daily				
PD 0332991		Days 1-21 each cycle				
**CLINICAL ASSESSMENT**						
Informed consent	X					
Concomitant medication	X	X		X	X	
H&P, PS	X	X		X	X	
Adverse event assessment	X	X	X	X	X	X
ECG	X					
Disease monitoring[B]	X	Per standard of care				
CBC+ differential	X	X	X	X	X	X
Serum chemistry [C]	X	X		X	X	X
Serum/urine pregnancy test[G]	X	X		X	X	
Drug compliance assessment[D]		X		X	X	
Research blood[E]		X		X (C12 only)	X	X

A. Premenopausal women requires goserelin
B. Disease monitoring is per standard of care.
C. Serum chemistry is albumin, alkaline phosphatase, total bilirubin, bicarbonate, BUN, calcium, chloride, creatinine, glucose, potassium, total protein, SGOT/AST, SGPT/ALT, sodium.
D. Patients will keep a pill diary (Appendix D).
E. Research blood includes the collection of plasma, serum and whole blood for cfDNA.

F. For patients who did not receive adjuvant PD 0332991 the follow up is yearly for 5 years post surgery or until recurrence whichever comes first.

G. In premenopausal women only.

11.0 DATA SUBMISSION SCHEDULE

Case report forms with appropriate source documentation will be completed according to the schedule listed in this section. Electronic data management systems will be used in this trial in collaboration with the Center for Biomedical Informatics core at Washington University.

ClinPortal is a web-based clinical studies data management system that will be used for capture of clinical data from this trial. The case report forms developed for this trial will be transformed to electronic format. An electronic study calendar will drive the study's data collection workflow. An Oracle database securely stores PHI in compliance with HIPAA and IRB regulations.

Identical study databases will be created in ClinPortal for each participating center; each center has access only to data from its own participants. Washington University, as the data coordinating center, has access to data from all sites.

Training in entering data in ClinPortal is required before a participating center will be given access to its site's database. The Center for Biomedical Informatics at Washington University offers monthly web-based training for external users; the schedule may be found at the following URL: http://cbmi.wustl.edu/?q=clinportal-training-details. Users must RSVP at least 3 days prior to the training sessions.

In addition, a participating center must have IRB approval of this protocol prior to initiation of ClinPortal data entry training, which must be completed prior to site study activation.

Case Report Form	Submission Schedule
Original Consent Form	Prior to (pre-) registration
Pre-registration Form<br>Registration Form<br>Eligibility Form<br>Pre-Study Form	Prior to starting treatment
Treatment Form<br>Labs Form	Every cycle
Adjuvant Form	Every cycle of adjuvant therapy
Toxicity Form	Continuous
Treatment Summary Form	Completion of treatment
Disease-Related Assessment Form	Day 1 of each cycle and end of Cycle 4
MedWatch Form	See Section 7.0 for reporting requirements
Surgery Form	1 month post surgery
Correlative Studies Form	Refer to Section 9.0
Follow-Up Form	Annually for 5 years after surgery

Any queries generated by Washington University must be responded to within 28 days of receipt by the participating site. The Washington University research team will conduct a regular review of data status at all secondary sites, with appropriate corrective action to be requested as needed.

12.0 MEASUREMENT OF EFFECT

12.1 Neoadjuvant Treatment

Complete cell cycle arrest: This is defined as Ki67 ≤ 2.7%, which will be assessed at C1D1, C1D15, and surgery. In patients without C1D15 Ki67, Ki67 on D15 of subsequent cycles could be used instead.

Clinical Evaluation: Prior to Cycle 0 (or performed during pre-registration for study NCI 9170), and at the end of each neoadjuvant treatment cycle cycles (that is, at the end of Cycles 1-4) the longest axis and the perpendicular axis of the measurable lesion should be measured and recorded in metric notation by tape, ruler or caliper technique on the case report forms.

Radiologic evaluation of tumor size: Mammogram and ultrasound imaging will be performed prior to Cycle 0 (or performed during pre-registration for study NCI 9170) and at the end of Cycle 4 combination therapy for bidimensional measurement of the tumor.

WHO criteria will be used to assess clinical and radiologic responses.

Complete Response (CR) is defined as the disappearance of all known disease based on a comparison between the pre-treatment measurements and the measurements taken at the completion of neo-adjuvant therapy (that is, at the end of cycle 4 neo-adjuvant combination therapy). In addition there is no appearance of new lesions.

Partial Response (PR) is defined as a 50% or greater decrease in the product of the bi-dimensional measurements of the lesion (total tumor size) between the pre-treatment measurements and the measurements taken at the completion of neo-adjuvant therapy (that is, at the end of cycle 4 neo-adjuvant combination therapy). In addition there can be no appearance of new lesions or progression of any lesion.

No Change (NC): a 50% decrease in total tumor size cannot be established nor has a 25% increase in the size of the lesion been demonstrated.

Progressive Disease (PD): A 25% or greater increase in the total tumor size of the lesion from its pretreatment measurements or the appearance of new lesions.

12.2 Treatment Resistance

A patient is said to have resistance disease if her Ki67 is > 10 % on biopsy taken on Cycle 1 Day 15 or if progressive disease is documented any time during neoadjuvant endocrine therapy.

12.3 Surgery

A pathologic complete response (pCR) is defined as no histology evidence of invasive tumor cells in the surgical breast specimen and sentinel or axillary lymph nodes.

All eligible women who have been begun treatment with combination therapy and have tumor Ki67 determined on C1D15 (or a subsequent cycle) are included in the analysis of complete cell cycle arrest. Women with tumor Ki67>2.7% on therapy are considered to have a non-complete cell cycle arrest.

12.4 Post-surgery

Local recurrence is defined as histologic evidence of ductal carcinoma in situ or invasive breast cancer in the ipsilateral breast or chest wall.

Regional recurrence is defined as the cytologic or histologic evidence of disease in the ipsilateral internal mammary, ipsilateral supraclavicular, ipsilateral infraclavicular and/or ipsilateral axillary nodes or soft tissue of the ipsilateral axilla.

Distant recurrence is defined as the cytologic, histologic, and/or radiographic evidence of disease in the skin, subcutaneous tissue, lymph nodes (other than local or regional metastasis), lung, bone narrow, central nervous system or histologic and/or radiographic evidence of skeletal or liver metastasis.

Second primary breast cancer is defined histologic evidence of ductal carcinoma in situ or invasive breast cancer in the contralateral breast or chest wall.

Second primary cancer (non-breast) is defined as any non-breast second primary cancer other than squamous or basal cell carcinoma of the skin, melanoma in situ, or carcinoma in situ of the cervix is to be reported and should be confirmed histologically whenever possible.

13.0 DATA AND SAFETY MONITORING

In compliance with the Washington University Institutional Data and Safety Monitoring Plan, the Data and Safety Monitoring Committee (DSMC) will meet to review toxicity data at least every 6 months following the activation of the first secondary site. The report will be prepared by the statistician with assistance from the study team and will be submitted to the Quality Assurance and Safety Monitoring Committee (QASMC). This report will include:

- HRPO protocol number, protocol title, Principal Investigator name, data coordinator name, regulatory coordinator name, and statistician
- Date of initial HRPO approval, date of most recent consent HRPO approval/revision, date of HRPO expiration, date of most recent QA audit, study status, and phase of

study
- History of study including summary of substantive amendments; summary of accrual suspensions including start/stop dates and reason; and summary of protocol exceptions, error, or breach of confidentiality including start/stop dates and reason
- Study-wide target accrual and study-wide actual accrual including numbers from participating sites
- Protocol activation date at each participating site
- Average rate of accrual observed in year 1, year 2, and subsequent years at each participating site
- Expected accrual end date and accrual by site.
- Objectives of protocol with supporting data and list the number of participants who have met each objective
- Measures of efficacy
- Early stopping rules with supporting data and list the number of participants who have met the early stopping rules
- Summary of toxicities at all participating sites
- Abstract submissions/publications
- Summary of any recent literature that may affect the safety or ethics of the study

Until such a time as the first secondary site activates this protocol, a semi-annual DSM report to be prepared by the study team will be submitted to the QASM Committee beginning 6 months after study activation at Washington University.

The study principal investigator and Research Patient Coordinator will monitor for serious toxicities on an ongoing basis. Once the principal investigator or Research Patient Coordinator becomes aware of an adverse event, the AE will be reported to the HRPO and QASMC according to institutional guidelines.

A DSMC will consist of no fewer than 3 members including 2 clinical investigators and a biostatistician. Like investigators, DSMC members are subject to the Washington University School of Medicine policies regarding standards of conduct. Individuals invited to serve on the DSMC will disclose any potential conflicts of interest to the trial principal investigator and/or appropriate university officials, in accordance with institution policies. Potential conflicts that develop during a trial or a member's tenure on a DSMC must also be disclosed.

Refer to the Washington University Quality Assurance and Data Safety Monitoring Committee Policies and Procedures for full details on the responsibilities of the DSMC at http://www.siteman.wustl.edu/uploadedFiles/Research_Programs/Clinical_Research_Resources/Protocol_Review_and_Monitoring_Committee/QASMCQualityAssurcance.pdf

14.0 AUDITING

Since Washington University is the coordinating center, each site will be audited annually by Siteman Cancer Center personnel (QASMC) unless the outside institution has an auditing mechanism in place and can provide a report. The outside sites will be asked to send copies of all

audit materials, including source documentation. The audit notification will be sent to the Washington University Research Patient Coordinator, who will obtain the audit materials from the participating institution.

Notification of an upcoming audit will be sent to the research team one month ahead of the audit. Once accrual numbers are confirmed, and approximately 30 days prior to the audit, a list of the cases selected for review (up to 10 for each site) will be sent to the research team. However, if during the audit the need arises to review cases not initially selected, the research team will be asked to provide the additional charts within two working days.

Additional details regarding the auditing policies and procedures can be found at https://siteman.wustl.edu/wp-content/uploads/2015/10/QASMC-Policies-and-Procedures-03.31.2015.pdf

15.0 STATISTICAL CONSIDERATIONS

15.1 Purpose

This is an open label phase II trial consisting of three independent cohorts that are run separately—the PIK3CA wild type cohort, the PIK3CA mutant cohort, and the endocrine resistant cohort—in women with clinical stage 2 or 3 estrogen receptor positive and HER2 negative invasive breast cancer to assess the anti-tumor activity (in terms of rate of complete cell cycle arrest) and safety profile of neoadjuvant PD 0332991 in combination with anastrozole for postmenopausal women or with anastrozole and goserelin for premenopausal women.

15.2 Primary Endpoint

The primary endpoint of this trial is complete cell cycle arrest. All eligible women who begin combination treatment and have an evaluable tumor Ki67 on Cycle 1 Day 15 will be included in the analyses of the primary study endpoint.

A patient with tumor Ki67 value > 2.7% on Cycle 1 Day 15 of combination treatment is considered to not have had a complete cell cycle arrest.

15.3 Trial Design

The expectation for complete cell cycle arrest, defined as $\leq$ 2.7%, at 2-4 weeks post neoadjuvant aromatase inhibitor alone, is 44% based on data from Z1031 Cohort B. Since PIK3CA mutation status does not affect neoadjuvant Ki67 response, the expected rate of complete cell cycle arrest for the PIK3CA mutant and wild type cohorts for single agent anastrozole is expected to be 44%.

The study is designed to ensure the sample size for the PIK3CA wild type cohort for the primary endpoint analysis. For the PIK3CA wild type cohort, thirty-three eligible patients

will be enrolled. The sample size was chosen using Fleming's single-stage phase II design to test the hypothesis that adding PD 0332991 would result in at least 50% improvement (44% versus 66%) in the complete cell cycle arrest rate, with 80% power and at 1-sided 0.05 significance level. If at least 20 patients in the cohort meet the criteria for complete cell cycle arrest, this regimen would be considered for future randomized trials.

Patients pre-registered to this trial and found to have PIK3CA mutation in the tumor are eligible to receive therapy in the PIK3CA mutant cohort. However, the enrollment to both cohorts will stop when 33 patients with PIK3CA wild type tumors have been enrolled and analyzed for the primary endpoint.

For the PIK3CA mutant cohort, depending on the frequency of mutation, 14 to 17 patients will be enrolled. The sample size for mutant cohort is determined primarily based on clinical feasibility rather than statistical power. However, according to the general guidelines regarding the sample size for translational studies[103], a sample size of 10-20 patients would provide reasonable precision for the estimation of pilot information regarding efficacy. If 10 complete cell-cycle arrest are observed out of 15 patients, for example, we will have 80% confidence that the "true" rate will fall between is ranged 47% and 83%.

For the endocrine resistant cohort, a Simon optimal two stage phase II clinical trial design will be used to assess whether the complete cell cycle arrest rate is at most 5% against the alternative that the complete cell cycle arrest rate is at least 20%. The study design proposed below yields a 90% chance of detecting a complete cell cycle arrest rate of at least 20% at an alpha level of 0.1.

> Stage 1: Enroll 12 patients. If at least one complete cell cycle arrest is documented among these 12 patients, continue to Stage 2. Otherwise terminate enrollment for this patient cohort and declare that this regimen has insufficient anti-tumor activity in the neoadjuvant setting to recommend it for further testing in this patient population.
>
> Stage 2: Enroll an additional 25 patients. If at most 3 complete cell cycle arrests are documented among the 37 total patients enrolled, this regimen will be considered to have insufficient anti-tumor activity in the neoadjuvant setting to recommend it for further testing in this patient population. Otherwise, this regimen may be recommended for further testing in the neoadjuvant setting for this patient population. For such a design, we will only have 7% chance to erroneously stop the trial at the 1st-stage if the true the complete cell cycle arrest rate is at least 20%. Conversely, there will be 54% chance to stop the trial at the 1st-stage if the true the complete cell cycle arrest rate is 5% or less.

Adverse events, the pace of accrual, other scientific discoveries, or changes in standard of care will be taken into account in any decision to terminate this trial earlier than designed.

A 90% confidence interval for the true complete cell cycle arrest rate will be calculated using the Duffy-Santner approach.

15.4 Sample Size and Trial Duration

We anticipate that 4-6 patients per month with clinical stage 2 or 3 estrogen receptor positive and HER2 negative invasive breast cancer will be screened for the *PIK3CA*. Approximately 40% of the patients have breast cancer with PIK3CA hotspot mutations. Thus, approximately 55 women with clinical stage 2 or 3 estrogen receptor positive and HER2 negative invasive breast cancer will be screened to enroll 33 evaluable patients to the PIK3CA wild type cohort.

The period encompassing enrollment, study treatment, and surgery will be about 20 months.

For the endocrine resistant cohort, we anticipate enrolling about one patient per month, completing Stage 1 in the approximately one year. If Stage 1 is successful, we will consider adding more sites to the study.

15.5 Data Analysis

All data analyses will be performed separately for each cohort unless otherwise specified.

Demographic and clinical characteristics of the sample, as well as response, toxicity by grade, and loss to follow-up will be summarized using descriptive statistics. The rate of complete cell cycle arrest will be calculated and the corresponding 90% confidence interval (CI) will be provided. The rates for pCR and PEPI 0 and their 90% CIs will also be calculated. The differences in overall survival (OS) and relapse-free survival (RFS) for patients with or without complete cell cycle arrest on C1D1 will be described using Kaplan-Meier product limit estimator and compared by log-rank tests. Similar analysis will be performed for complete cell cycle arrest on C1D15 or patients with or without PEPI 0, with the two cohorts combined or separately. A trajectory analysis (see below) will also be performed for this comparison.

Analysis of other secondary endpoints:

Clinical response rate: The clinical response rate will be estimated by the number of patients whose disease meets the WHO criteria of complete or partial response prior to surgery divided by the total number of eligible patients who began combination neoadjuvant treatment. A 90% confidence interval for the true clinical response rate will be calculated using the Duffy-Santner approach for the endocrine resistant cohort (2-stage design). The complete cell cycle arrest rates and the corresponding 90% confidence intervals will also be calculated for PIK3CA wild type and mutant cohorts separately and combined.

Radiological response rate: The radiological response rate will be estimated by the number of patients whose disease meets with WHO criteria for complete or partial response at the evaluation prior to surgery divided by the total number of eligible patients who began combination neo-adjuvant therapy. A 90% confidence interval for the true radiographic response rate will be calculated using the Duffy-Santner approach. The analysis will be performed with the two cohorts separately and combined.

Adverse Events: All eligible patients that have intitiated treatment will be considered evaluable for assessing adverse event rate(s). The maximum grade for each type of adverse event will be recorded for each patient using the NCI-CTCAE v4.0, and frequency tables will be reviewed to determine patterns. Additionally, the relationship of the adverse event(s) to the study treatment will be taken into consideration.

Ki67 suppression on Cycle 1 Day 1, Cycle 1 Day 15 and surgery: Following the approach of Dowsett et al. [26], the percent change in the Ki67 level from baseline will be determined on the log scale. A 95% t-confidence interval for the mean percent change in the Ki67 level from baseline will be constructed (if appropriate). The rate of complete cell cycle arrest on C1D1, C1D15, and surgery will be described by contingency tables compared by McNemar tests. Using trajectory analysis, we will also assess the association between distinct patterns of Ki67 change over time and clinical outcomes. Specifically, trajectory groups will be formulated to describe clusters of individuals with similar temporal patterns of Ki67 over time using finite mixture models with SAS procedure TRAJ. This method enables identification of the number of discrete trajectory types, and characterization of the shape and estimation of the prevalence of each type. We will choose the best models based on statistical tests (including Bayesian and Akaike information criterion, each of which test goodness-of-fit) and clinical judgment.

To assess the concentrations of anastrozole and PD 0332991: The concentrations of anastrozole prior to and 90 minutes following anastrozole (without PD 0332991) on Cycle 1 Day 1, as well as the concentrations of anastrozole prior to and 90 minutes following both anastrozole and PD 0332991 on Cycle 1 Day 15 will be summarized using means and standard deviations, and the differneces will be compared using two-way ANOVA for repeated measurement data. Data transformation will also be performed as necessary to better satisfy normality assumption. Similar analysis will be performed for the concentration of of PD 0332991.

To assess the long term outcomes of patients treated in this trial: The long-term outcomes (i.e., pathologic complete response (pCr), local and distant recurrence, etc, as defined in section 12.4) will also be assessed in the PIK3CA wild and mutnt cohorts (separately and combined) and the endocrine resistant cohort, respectively. The categorical outcome will be summarized using contingency tables and compared by Fisher's exact test, while the time-to-event outcomes will be described using Kaplan-Meier product limit estimators and compared by log-rank test.

15.6 Correlative Studies

Tumor cell apoptotic index: Apoptosis studies based on the TUNEL assay conducted in the context of the IMPACT study showed that the rate of apoptosis actually *declined* after two weeks of anastrozole treatment when the percentage TUNEL positive cells was less than one percent, with an SD of less than one percent [12]. Given the very low background of TUNEL positivity, a modest sized study is sufficient to demonstrate an increase in cell death. We will use the TUNEL assay to assess apoptosis at baseline and in the sample taken on PD 0332991. The TUNEL-positive cells will be counted using the point counting approach used for Ki67 and the apoptosis index will be calculated as the number of positive cells divided by the number of all cells and multiplied by 100.

Steady state levels of estradiol: Steady state levels of estradiol on serially collected serum samples will be examined in premenopausal women to confirm the efficacy of the anastrozole goserelin therapy in suppressing both ovarian and peripheal estradiol production. The change of estradiol level over time will be described by summary statistics and also compared using 2-way ANOVA for repeated measurement data

Pharmacodynamic markers and markers of tumor cell senescence: The pharmacodynamic effect of PD 0332991 in combination with anastrozole on RB phosphorylation and markers of senescence will be assessed by phosphoroproteomics and immunohistochemistry analysis on serial tumor biopsies. The change of RB phosphorylation and senescence markers over time will be described by summary statistics and also compared using 2-way ANOVA for repeated measurement data.

Explore potential markers of sensitivity and resistance: In preclinical studies, RB proficient cell lines with low p16 expression were found to be most sensitive to PD 0332991 in ovarian cancer. In addition, copy number variations of CDKN2A, RB, CCNE1, CCND1 were associated with response [102]. Therefore, tumor specimens collected on Cycle 1 day 1 will be analyzed for the protein expression of these genes by immunohistochemistry. Gene copy number changes will be assessed by circular binary segment (CBS) analysis using Signal Map Software (Nimblegen, Madison, WI, USA) and its association with clinical outcomes will also be assessed using contingency tables and fisher's exact test. The gene expression levels between responses will also be summarized using means, standard deviations, medians, and compared by t-test or Mann-Whitney rank-sum test as appropriate.

16.0 MULTICENTER REGULATORY REQUIREMENTS

Washington University requires that each participating site sends its informed consent document to be reviewed and approved by the Washington University Regulatory Coordinator (or designee) prior to IRB/IEC submission.

Site activation is defined as when the secondary site has received official written documentation from the coordinating center that the site has been approved to begin enrollmkent. At a minimum, each participating institution must have the following documents on file at Washington University prior to study activation:

- Documentation of IRB approval of the study in the form of a letter or other official document from the participating institution's IRB. This documentation must show which version of the protocol was approved by the IRB.
- Documentation of IRB approval of an informed consent form. The consent must include a statement that data will be shared with Washington University, including the Quality Assurance and Safety Monitoring Committee (QASMC), the DSMC (if applicable), and the Washington University study team.
- Documentation of FWA, signed FDA Form 1572 (if applicable), and the CVs of all participating investigators.
- Protocol signature page signed and dated by the investigator at each participating site.

The coordinating center Principal Investigator (or designee) is responsible for disseminating to the participating sites all study updates, amendments, reportable adverse events, etc. Protocol/consent modifications and IB updates will be forwarded electronically to the secondary sites within 4 weeks of obtaining Washington University IRB approval. Activated secondary sites are expected to submit protocol/consent/IB modifications to their local IRBs within 4 weeks of receipt unless otherwise noted. Upon the secondary sites obtaining local IRB approval, documentation of such shall be sent to the Washington University study team within 2 weeks of receipt of approval.

Documentation of participating sites' IRB approval of annual continuing reviews, protocol amendments or revisions, all SAE reports, and all protocol violations/deviations/exceptions must be kept on file at Washington University.

The investigator or a designee from each institution must participate in a regular conference call to update and inform regarding the progress of the trial.

17.0 REFERENCES

1. Jemal, A., et al., *Cancer statistics, 2010.* CA Cancer J Clin, 2010. **60**(5): p. 277-300.
2. EBCTCG, *Effects of chemotherapy and hormonal therapy for early breast cancer on recurrence and 15-year survival: an overview of the randomised trials.* Lancet, 2005. **365**(9472): p. 1687-717.
3. Darb-Esfahani, S., et al., *Identification of biology-based breast cancer types with distinct predictive and prognostic features: role of steroid hormone and HER2 receptor expression in patients treated with neoadjuvant anthracycline/taxane-based chemotherapy.* Breast cancer research : BCR, 2009. **11**(5): p. R69.
4. Liedtke, C., et al., *Response to neoadjuvant therapy and long-term survival in patients with triple-negative breast cancer.* Journal of clinical oncology : official journal of the American Society of Clinical Oncology, 2008. **26**(8): p. 1275-81.
5. Carey, L.A., et al., *The triple negative paradox: primary tumor chemosensitivity of breast cancer subtypes.* Clinical cancer research : an official journal of the American Association for Cancer Research, 2007. **13**(8): p. 2329-34.
6. Guarneri, V., et al., *Prognostic value of pathologic complete response after primary chemotherapy in relation to hormone receptor status and other factors.* J Clin Oncol, 2006. **24**(7): p. 1037-44.
7. Bonadonna, G., et al., *Primary chemotherapy to avoid mastectomy in tumors with diameters of three centimeters or more.* Journal of the National Cancer Institute, 1990. **82**(19): p. 1539-45.
8. Kuerer, H.M., et al., *Incidence and impact of documented eradication of breast cancer axillary lymph node metastases before surgery in patients treated with neoadjuvant chemotherapy.* Annals of surgery, 1999. **230**(1): p. 72-8.
9. Mauriac, L., et al., *Effects of primary chemotherapy in conservative treatment of breast cancer patients with operable tumors larger than 3 cm. Results of a randomized trial in a single centre.* Annals of oncology : official journal of the European Society for Medical Oncology / ESMO, 1991. **2**(5): p. 347-54.
10. Ellis, M.J., *Improving outcomes for patients with hormone receptor-positive breast cancer: back to the drawing board.* J Natl Cancer Inst, 2008. **100**(3): p. 159-61.
11. Ellis, M.J., et al., *Outcome prediction for estrogen receptor-positive breast cancer based on postneoadjuvant endocrine therapy tumor characteristics.* J Natl Cancer Inst, 2008. **100**(19): p. 1380-8.
12. Dowsett, M., et al., *Proliferation and apoptosis as markers of benefit in neoadjuvant endocrine therapy of breast cancer.* Clin Cancer Res, 2006. **12**(3 Pt 2): p. 1024s-1030s.
13. Olson, J.A., et al., *Improved Surgical Outcomes for Breast Cancer Patients Receiving Neoadjuvant Aromatase Inhibitor Therapy: Results from a Multicenter Phase II Trial.* J Am Coll Surg, 2009. **in press**.
14. Eiermann, W., et al., *Preoperative treatment of postmenopausal breast cancer patients with letrozole: A randomized double-blind multicenter study.* Ann Oncol, 2001. **12**(11): p. 1527-32.
15. Baselga, J., et al., *Phase II Randomized Study of Neoadjuvant Everolimus Plus Letrozole Compared With Placebo Plus Letrozole in Patients With Estrogen Receptor–Positive Breast Cancer.* Journal of Clinical Oncology, 2009. **27**(16): p. 2630-2637.

16. Choi, Y.J., et al., *The requirement for cyclin D function in tumor maintenance.* Cancer Cell, 2012. **22**(4): p. 438-51.
17. Campisi, J. and F. d'Adda di Fagagna, *Cellular senescence: when bad things happen to good cells.* Nat Rev Mol Cell Biol, 2007. **8**(9): p. 729-40.
18. Rader, J., et al., *Dual CDK4/CDK6 Inhibition Induces Cell Cycle Arrest and Senescence in Neuroblastoma.* Clinical Cancer Research, 2013.
19. Anders, L., et al., *A systematic screen for CDK4/6 substrates links FOXM1 phosphorylation to senescence suppression in cancer cells.* Cancer Cell, 2011. **20**(5): p. 620-34.
20. Thangavel, C., et al., *Therapeutically activating RB: reestablishing cell cycle control in endocrine therapy-resistant breast cancer.* Endocrine-related cancer, 2011. **18**(3): p. 333-45.
21. Network, T.C.G.A., *Comprehensive molecular portraits of human breast tumours.* Nature, 2012. **490**(7418): p. 61-70.
22. Finn, R.S., et al., *Results of a randomized phase 2 study of PD 0332991, a cyclin-dependent kinase (CDK) 4/6 inhibitor, in combination with letrozole vs letrozole alone for first-line treatment of ER+/HER2– advanced breast cancer (BC)* Cancer Research, 2012. **72**(24 (Suppl 3)): p. Thirty-Fifth Annual CTRC-AACR San Antonio Breast Cancer Symposium-- Dec 4-8, 2012; San Antonio, TX, abstract number S1-6.
23. Ma, C.X. and M.J. Ellis, *Neoadjuvant endocrine therapy for locally advanced breast cancer.* Seminars in oncology, 2006. **33**(6): p. 650-6.
24. Ellis, M.J., et al., *Aromatase expression and outcomes in the P024 neoadjuvant endocrine therapy trial.* Breast Cancer Res Treat, 2008.
25. Dowsett, M., et al., *Prognostic value of Ki67 expression after short-term presurgical endocrine therapy for primary breast cancer.* J Natl Cancer Inst, 2007. **99**(2): p. 167-70.
26. Dowsett, M., et al., *Short-term changes in Ki-67 during neoadjuvant treatment of primary breast cancer with anastrozole or tamoxifen alone or combined correlate with recurrence-free survival.* Clin Cancer Res, 2005. **11**(2 Pt 2): p. 951s-8s.
27. Tao, Y., et al., *Clinical and biomarker endpoint analysis in neoadjuvant endocrine therapy trials.* J Steroid Biochem Mol Biol, 2005. **95**(1-5): p. 91-5.
28. Ellis, M.J., et al., *Randomized phase II neoadjuvant comparison between letrozole, anastrozole, and exemestane for postmenopausal women with estrogen receptor-rich stage 2 to 3 breast cancer: clinical and biomarker outcomes and predictive value of the baseline PAM50-based intrinsic subtype--ACOSOG Z1031.* Journal of clinical oncology : official journal of the American Society of Clinical Oncology, 2011. **29**(17): p. 2342-9.
29. Jakesz, R., et al., *Randomized adjuvant trial of tamoxifen and goserelin versus cyclophosphamide, methotrexate, and fluorouracil: evidence for the superiority of treatment with endocrine blockade in premenopausal patients with hormone-responsive breast cancer--Austrian Breast and Colorectal Cancer Study Group Trial 5.* J Clin Oncol, 2002. **20**(24): p. 4621-7.
30. Jonat, W., et al., *A randomised study to compare the effect of the luteinising hormone releasing hormone (LHRH) analogue goserelin with or without tamoxifen in pre- and perimenopausal patients with advanced breast cancer.* Eur J Cancer, 1995. **31A**(2): p. 137-42.
31. Park, I.H., et al., *Phase II parallel group study showing comparable efficacy between premenopausal metastatic breast cancer patients treated with letrozole plus goserelin*

and postmenopausal patients treated with letrozole alone as first-line hormone therapy. J Clin Oncol, 2010. **28**(16): p. 2705-11.
32. Sagara, Y., et al. *The STAGE Study: A Phase III Comparison of Anastrozole Plus Goserelin with Tamoxifen Plus Goserelin as Pre-Operative Treatments in Premenopausal Breast Cancer Patients.* in *San Antonio Breast Cancer Symposium*. 2010. San Antonio, Texas.
33. Kinoshita, T., et al., *Neoadjuvant anastrozole or tamoxifen for premenopausal breast cancer: Ki67 expression data from the STAGE study.* Journal of Clinical Oncology, 2011. **29**(suppl): p. abstr 501.
34. Leung, B.S. and A.H. Potter, *Mode of estrogen action on cell proliferation in CAMA-1 cells: II. Sensitivity of G1 phase population.* J Cell Biochem, 1987. **34**(3): p. 213-25.
35. Sutherland, R.L., R.E. Hall, and I.W. Taylor, *Cell proliferation kinetics of MCF-7 human mammary carcinoma cells in culture and effects of tamoxifen on exponentially growing and plateau-phase cells.* Cancer Res, 1983. **43**(9): p. 3998-4006.
36. Sherr, C.J., *Cancer cell cycles.* Science, 1996. **274**(5293): p. 1672-7.
37. Sherr, C.J. and J.M. Roberts, *CDK inhibitors: positive and negative regulators of G1-phase progression.* Genes & development, 1999. **13**(12): p. 1501-12.
38. van den Heuvel, S. and E. Harlow, *Distinct roles for cyclin-dependent kinases in cell cycle control.* Science, 1993. **262**(5142): p. 2050-4.
39. Weinberg, R.A., *The retinoblastoma protein and cell cycle control.* Cell, 1995. **81**(3): p. 323-30.
40. Roussel, M.F., et al., *Rescue of defective mitogenic signaling by D-type cyclins.* Proc Natl Acad Sci U S A, 1995. **92**(15): p. 6837-41.
41. Sherr, C.J., *D-type cyclins.* Trends in biochemical sciences, 1995. **20**(5): p. 187-90.
42. Altucci, L., et al., *Estrogen induces early and timed activation of cyclin-dependent kinases 4, 5, and 6 and increases cyclin messenger ribonucleic acid expression in rat uterus.* Endocrinology, 1997. **138**(3): p. 978-84.
43. Geum, D., et al., *Estrogen-induced cyclin D1 and D3 gene expressions during mouse uterine cell proliferation in vivo: differential induction mechanism of cyclin D1 and D3.* Molecular reproduction and development, 1997. **46**(4): p. 450-8.
44. Said, T.K., et al., *Progesterone, in addition to estrogen, induces cyclin D1 expression in the murine mammary epithelial cell, in vivo.* Endocrinology, 1997. **138**(9): p. 3933-9.
45. Tong, W. and J.W. Pollard, *Progesterone inhibits estrogen-induced cyclin D1 and cdk4 nuclear translocation, cyclin E- and cyclin A-cdk2 kinase activation, and cell proliferation in uterine epithelial cells in mice.* Molecular and cellular biology, 1999. **19**(3): p. 2251-64.
46. Hui, R., et al., *Cyclin D1 and estrogen receptor messenger RNA levels are positively correlated in primary breast cancer.* Clinical cancer research : an official journal of the American Association for Cancer Research, 1996. **2**(6): p. 923-8.
47. Michalides, R., et al., *A clinicopathological study on overexpression of cyclin D1 and of p53 in a series of 248 patients with operable breast cancer.* British journal of cancer, 1996. **73**(6): p. 728-34.
48. Nielsen, N.H., et al., *Deregulation of cyclin E and D1 in breast cancer is associated with inactivation of the retinoblastoma protein.* Oncogene, 1997. **14**(3): p. 295-304.
49. Altucci, L., et al., *17beta-Estradiol induces cyclin D1 gene transcription, p36D1-p34cdk4 complex activation and p105Rb phosphorylation during mitogenic stimulation of G(1)-*

arrested human breast cancer cells. Oncogene, 1996. **12**(11): p. 2315-24.
50. Foster, J.S. and J. Wimalasena, *Estrogen regulates activity of cyclin-dependent kinases and retinoblastoma protein phosphorylation in breast cancer cells.* Molecular endocrinology, 1996. **10**(5): p. 488-98.
51. Planas-Silva, M.D. and R.A. Weinberg, *Estrogen-dependent cyclin E-cdk2 activation through p21 redistribution.* Molecular and cellular biology, 1997. **17**(7): p. 4059-69.
52. Prall, O.W., et al., *Estrogen-induced activation of Cdk4 and Cdk2 during G1-S phase progression is accompanied by increased cyclin D1 expression and decreased cyclin-dependent kinase inhibitor association with cyclin E-Cdk2.* The Journal of biological chemistry, 1997. **272**(16): p. 10882-94.
53. Lukas, J., J. Bartkova, and J. Bartek, *Convergence of mitogenic signalling cascades from diverse classes of receptors at the cyclin D-cyclin-dependent kinase-pRb-controlled G1 checkpoint.* Molecular and cellular biology, 1996. **16**(12): p. 6917-25.
54. Watts, C.K., et al., *Antiestrogen inhibition of cell cycle progression in breast cancer cells in associated with inhibition of cyclin-dependent kinase activity and decreased retinoblastoma protein phosphorylation.* Molecular endocrinology, 1995. **9**(12): p. 1804-13.
55. McMahon, C., et al., *P/CAF associates with cyclin D1 and potentiates its activation of the estrogen receptor.* Proc Natl Acad Sci U S A, 1999. **96**(10): p. 5382-7.
56. Neuman, E., et al., *Cyclin D1 stimulation of estrogen receptor transcriptional activity independent of cdk4.* Mol Cell Biol, 1997. **17**(9): p. 5338-47.
57. Zwijsen, R.M., et al., *Ligand-independent recruitment of steroid receptor coactivators to estrogen receptor by cyclin D1.* Genes Dev, 1998. **12**(22): p. 3488-98.
58. Zwijsen, R.M., et al., *CDK-independent activation of estrogen receptor by cyclin D1.* Cell, 1997. **88**(3): p. 405-15.
59. Harper, J.W., et al., *The p21 Cdk-interacting protein Cip1 is a potent inhibitor of G1 cyclin-dependent kinases.* Cell, 1993. **75**(4): p. 805-16.
60. Koff, A., et al., *Negative regulation of G1 in mammalian cells: inhibition of cyclin E-dependent kinase by TGF-beta.* Science, 1993. **260**(5107): p. 536-9.
61. Polyak, K., et al., *p27Kip1, a cyclin-Cdk inhibitor, links transforming growth factor-beta and contact inhibition to cell cycle arrest.* Genes & development, 1994. **8**(1): p. 9-22.
62. Toyoshima, H. and T. Hunter, *p27, a novel inhibitor of G1 cyclin-Cdk protein kinase activity, is related to p21.* Cell, 1994. **78**(1): p. 67-74.
63. Lukas, J., et al., *Retinoblastoma-protein-dependent cell-cycle inhibition by the tumour suppressor p16.* Nature, 1995. **375**(6531): p. 503-6.
64. Medema, R.H., et al., *Growth suppression by p16ink4 requires functional retinoblastoma protein.* Proceedings of the National Academy of Sciences of the United States of America, 1995. **92**(14): p. 6289-93.
65. Parry, D., et al., *Lack of cyclin D-Cdk complexes in Rb-negative cells correlates with high levels of p16INK4/MTS1 tumour suppressor gene product.* EMBO J, 1995. **14**(3): p. 503-11.
66. Jiang, H., H.S. Chou, and L. Zhu, *Requirement of cyclin E-Cdk2 inhibition in p16(INK4a)-mediated growth suppression.* Mol Cell Biol, 1998. **18**(9): p. 5284-90.
67. McConnell, B.B., et al., *Induced expression of p16(INK4a) inhibits both CDK4- and CDK2-associated kinase activity by reassortment of cyclin-CDK-inhibitor complexes.* Mol Cell Biol, 1999. **19**(3): p. 1981-9.

68. Mitra, J., et al., *Induction of p21(WAF1/CIP1) and inhibition of Cdk2 mediated by the tumor suppressor p16(INK4a).* Mol Cell Biol, 1999. **19**(5): p. 3916-28.
69. Craig, C., et al., *Effects of adenovirus-mediated p16INK4A expression on cell cycle arrest are determined by endogenous p16 and Rb status in human cancer cells.* Oncogene, 1998. **16**(2): p. 265-72.
70. Craig, C., et al., *A recombinant adenovirus expressing p27Kip1 induces cell cycle arrest and loss of cyclin-Cdk activity in human breast cancer cells.* Oncogene, 1997. **14**(19): p. 2283-9.
71. Foster, J.S., et al., *Multifaceted regulation of cell cycle progression by estrogen: regulation of Cdk inhibitors and Cdc25A independent of cyclin D1-Cdk4 function.* Molecular and cellular biology, 2001. **21**(3): p. 794-810.
72. Buckley, M., et al., *Expression and amplification of cyclin genes in human breast cancer.* Oncogene, 1993. **8**: p. 2127 - 2133.
73. Dickson, C., et al., *Amplification of chromosome band 11q13 and a role for cyclin D1 in human breast cancer.* Cancer letters, 1995. **90**(1): p. 43-50.
74. Lammie, G.A., et al., *D11S287, a putative oncogene on chromosome 11q13, is amplified and expressed in squamous cell and mammary carcinomas and linked to BCL-1.* Oncogene, 1991. **6**(3): p. 439-44.
75. Bartkova, J., et al., *Cyclin D1 protein expression and function in human breast cancer.* Int J Cancer, 1994. **57**(3): p. 353-61.
76. Gillett, C., et al., *Amplification and overexpression of cyclin D1 in breast cancer detected by immunohistochemical staining.* Cancer Res, 1994. **54**(7): p. 1812-7.
77. McIntosh, G.G., et al., *Determination of the prognostic value of cyclin D1 overexpression in breast cancer.* Oncogene, 1995. **11**(5): p. 885-91.
78. Gillett, C., et al., *Cyclin D1 and prognosis in human breast cancer.* Int J Cancer, 1996. **69**(2): p. 92-9.
79. Knudsen, K.E., et al., *Cyclin D1: polymorphism, aberrant splicing and cancer risk.* Oncogene, 2006. **25**(11): p. 1620-8.
80. Yu, Q., et al., *Requirement for CDK4 kinase function in breast cancer.* Cancer cell, 2006. **9**(1): p. 23-32.
81. Pietilainen, T., et al., *Expression of retinoblastoma gene protein (Rb) in breast cancer as related to established prognostic factors and survival.* Eur J Cancer, 1995. **31A**(3): p. 329-33.
82. Borg, A., et al., *The retinoblastoma gene in breast cancer: allele loss is not correlated with loss of gene protein expression.* Cancer research, 1992. **52**: p. 2991 - 2994.
83. Oesterreich, S. and S.A. Fuqua, *Tumor suppressor genes in breast cancer.* Endocr Relat Cancer, 1999. **6**(3): p. 405-19.
84. Chano, T., et al., *Truncating mutations of RB1CC1 in human breast cancer.* Nature genetics, 2002. **31**: p. 285 - 288.
85. Bosco, E., et al., *The retinoblastoma tumor suppressor modifies the therapeutic response of breast cancer.* The Journal of clinical investigation, 2007. **117**: p. 218 - 228.
86. Musgrove, E.A., et al., *Expression of the cyclin-dependent kinase inhibitors p16INK4, p15INK4B and p21WAF1/CIP1 in human breast cancer.* International journal of cancer. Journal international du cancer, 1995. **63**(4): p. 584-91.
87. Zhou, R., et al., *The growth arrest function of the human oncoprotein mouse double minute-2 is disabled by downstream mutation in cancer cells.* Cancer research, 2005.

65(5): p. 1839-48.
88. Finn, R.S., et al., *PD 0332991, a selective cyclin D kinase 4/6 inhibitor, preferentially inhibits proliferation of luminal estrogen receptor-positive human breast cancer cell lines in vitro.* Breast cancer research : BCR, 2009. **11**(5): p. R77.
89. Fry, D.W., et al., *Specific inhibition of cyclin-dependent kinase 4/6 by PD 0332991 and associated antitumor activity in human tumor xenografts.* Molecular cancer therapeutics, 2004. **3**(11): p. 1427-38.
90. Goncalves, R., et al., *A Ki-67-based clinical trial assay for neoadjuvant endocrine therapy response monitoring in breast cancer.* submitted.
91. Saal, L.H., et al., *PIK3CA mutations correlate with hormone receptors, node metastasis, and ERBB2, and are mutually exclusive with PTEN loss in human breast carcinoma.* Cancer Res, 2005. **65**(7): p. 2554-9.
92. Kalinsky, K., et al., *PIK3CA mutation associates with improved outcome in breast cancer.* Clin Cancer Res, 2009. **15**(16): p. 5049-59.
93. Ellis, M.J., et al., *Whole-genome analysis informs breast cancer response to aromatase inhibition.* Nature, 2012. **486**(7403): p. 353-60.
94. Samuels, Y., et al., *High frequency of mutations of the PIK3CA gene in human cancers.* Science, 2004. **304**(5670): p. 554.
95. Zhang, H., et al., *Comprehensive analysis of oncogenic effects of PIK3CA mutations in human mammary epithelial cells.* Breast Cancer Res Treat, 2008. **112**(2): p. 217-27.
96. Zhao, J.J., et al., *The oncogenic properties of mutant p110alpha and p110beta phosphatidylinositol 3-kinases in human mammary epithelial cells.* Proc Natl Acad Sci U S A, 2005. **102**(51): p. 18443-8.
97. Crowder, R.J., et al., *PIK3CA and PIK3CB inhibition produce synthetic lethality when combined with estrogen deprivation in estrogen receptor-positive breast cancer.* Cancer Res, 2009. **69**(9): p. 3955-62.
98. Ellis, M.J., et al., *Phosphatidyl-inositol-3-kinase alpha catalytic subunit mutation and response to neoadjuvant endocrine therapy for estrogen receptor positive breast cancer.* Breast Cancer Res Treat, 2010. **119**(2): p. 379-90.
99. Nielsen, T.O., et al., *A comparison of PAM50 intrinsic subtyping with immunohistochemistry and clinical prognostic factors in tamoxifen-treated estrogen receptor-positive breast cancer.* Clin Cancer Res, 2010. **16**(21): p. 5222-32.
100. Baum, M., et al., *Anastrozole alone or in combination with tamoxifen versus tamoxifen alone for adjuvant treatment of postmenopausal women with early breast cancer: first results of the ATAC randomised trial.* Lancet, 2002. **359**(9324): p. 2131-9.
101. Leontieva, O.V. and M.V. Blagosklonny, *CDK4/6-inhibiting drug substitutes for p21 and p16 in senescence: Duration of cell cycle arrest and MTOR activity determine geroconversion.* Cell Cycle, 2013. **12**(18): p. 3063-9.
102. Konecny, G.E., et al., *Expression of p16 and retinoblastoma determines response to CDK4/6 inhibition in ovarian cancer.* Clinical cancer research : an official journal of the American Association for Cancer Research, 2011. **17**(6): p. 1591-602.
103. Piantadosi, S., *Translational clinical trials: an entropy-based approach to sample size.* Clin Trials, 2005. **2**(2): p. 182-92.

APPENDIX A: ECOG Performance Status Scale

Grade	Description
0	Normal activity. Fully active, able to carry on all pre-disease performance without restriction.
1	Symptoms, but ambulatory. Restricted in physically strenuous activity, but ambulatory and able to carry out work of a light or sedentary nature (e.g., light housework, office work).
2	In bed <50% of the time. Ambulatory and capable of all self-care, but unable to carry out any work activities. Up and about more than 50% of waking hours.
3	In bed >50% of the time. Capable of only limited self-care, confined to bed or chair more than 50% of waking hours.
4	100% bedridden. Completely disabled. Cannot carry on any self-care. Totally confined to bed or chair.
5	Dead.

APPENDIX B: Neoadjuvant PD 0332991: Registration Worksheet

Protocol #:	Neoadjuvant PD 0332991 (HRPO# 201301106)	Patient Initials	________-_________-______
PI Name:		Institution Name:	
Registering MD name:		Site Number:	
Study Coordinator Name and Email:		Site Telephone #	

Please ensure the following are included with this form: ***(Please ensure all patient identification information is removed)***

☐ Corresponding Source Documentation for Inclusion/Exclusion

**Menopausal Status**	**☐ Pre/Peri- menopausal**	**☐ Postmenopausal**
**DOB:**	_____ / _____ / _____	
**Zip Code:**	____ ____ ____ ____ ____	
**Date endocrine therapy initiated:**	_____ / _____ / _____	
**Local Ki67 after at least 2 weeks of endocrine therapy:**	________________	
**Planned Date Investigational Treatment Initiation:**	_____ / _____ / _____	
**Date of signed informed consent:**	_____ / _____ / _____	

REGISTRATION INCLUSION CRITERIA		
☐	1. Clinical T2-T4c at diagnosis or screening, any N, M0 invasive ER+ (Allred Score at least 3 or > 1% ER positivity) and HER2 negative (0 or 1+ by IHC or FISH negative equivocal) breast cancer, by AJCC 7th edition clinical staging, with the goal being surgery to completely excise the tumor in the breast and the lymph node.<br><br>Note: If the patient has invasive breast cancer that is ER pos, HER2 neg or equivocal, or DCIS in the contralateral breast, the patient is eligible. Multifocal diseases are not excluded.	
☐	2. Ki 67 > 10% by central testing after at least 2 weeks on neoadjuvant endocrine therapy.	
☐	3. Female ≥18 years of age.	
☐	4. ECOG performance status of 0, 1 or 2	ECOG: _________<br>Date Assessed: ____________
☐	5. If premenopausal, patient must be willing to comply with pregnancy requirements laid out in Section 5.5.	
	6. Laboratory Values (Within 14 days of Registration)	
☐	Leukocytes ≥ 3,000/mcL	Screening result:____________
☐	Absolute Neutrophil Count (ANC) ≥ 1,500/mcL	Screening result:____________
☐	Platelets (Plts) ≥ 100,000/mcL	Screening result:____________
☐	Total bilirubin ≤ upper normal institutional limits	Screening result:____________<br>ULN:____________
☐	Alanine aminotransferase (ALT) and aspartate aminotransferase (AST) ≤ 2.5 x upper limit of normal (ULN)	ALT screening result:__________<br>ULN:___________<br>AST screening result:_________<br>ULN:____________
☐	Serum creatinine ≤ upper normal institutional limits	Screening result:____________<br>ULN:____________
☐	7. Able to understand and willing to sign an IRB-approved written informed consent document	

	REGISTRATION EXCLUSION CRITERIA	
☐	1. Prior treatment of this cancer including: surgery, radiation therapy, or chemotherapy.	
☐	2. Receiving any other investigational agents.	
☐	3. Prior therapy with any Cdk4 inhibitor	
☐	4. Any of the following in the previous 6 months: myocardial infarction, severe/unstable angina, coronary/peripheral artery bypass graft, symptomatic congestive heart failure, cerebrovascular accident, transient ischemic attack, symptomatic pulmonary embolism	
☐	5. Uncontrolled intercurrent illness including, but not limited to ongoing or active infection, symptomatic congestive heart failure, unstable angina pectoris, uncontrolled symptomatic cardiac arrhythmia, psychiatric illness/social situations that would limit compliance with study requirements	
☐	6. Pregnant/nursing.	
☐	7. Unwilling to employ adequate contraception.	
☐	8. Known HIV-positive on combination antiretroviral therapy.	
☐	9. Known metastatic disease	
☐	10. Current use of anticoagulation therapy	
☐	11. Previous excisional biopsy of the breast cancer or sentinel lymph node biopsy	
☐	12. Any condition that impairs patient's ability to swallow PD 0332991 tablets (*e.g.*, gastrointestinal tract disease resulting in an inability to take oral medication or a requirement for IV alimentation, prior surgical procedures affecting absorption)	
☐	13. History of allergic reactions attributed to compounds of similar chemical or biologic composition to PD 0332991 or other agents used in the study	
☐	14. Correct QT (QTc) interval > 470 msec	QTc: ______________<br>Date:______________
	15.	
☐	16. Current use or anticipated need for food or drugs that are known strong CYP3A4 inhibitors (i.e. grapefruit juice, verapamil, ketoconazole, miconazole, itraconazole, posaconazole, erythromycin, clarithromycin, telithromycin, indinavir, saquinavir, ritonavir, nelfinavir, lopinavir, atazanavir, amprenavir, fosamprenavir, nefazodone, diltiazem, and delavirdine) or inducers (i.e. dexamethasone, glucocorticoids, progesterone, rifampin, phenobarbital, St. John's wort).	
	17.	

Completed by: ___________________________ Date: _____ / _____ / _____

Investigator Signature: _________________________ Date: _____ / _____ / _____

For Wash U Use Only

Screen Failure ☐ Yes ☐ No

Subject Identifier: __ __ - __ __ __

Signature (Wash U designee): ___________________________ Date: _____ / _____ / _____

APPENDIX C: Adjuvant PD 0332991 Registration Worksheet

Protocol #:	Neoadjuvant PD 0332991 (HRPO# 201301106)	Patient Initials	________-_________-______
PI Name:		Institution Name:	
Registering MD name:		Site Number:	
Study Coordinator Name and Email:		Site Telephone #	

Please ensure the following are included with this form: ***(Please ensure all patient identification information is removed)***

☐ Corresponding Source Documentation for Inclusion/Exclusion

**Menopausal Status**	**☐ Pre/Peri- menopausal**	**☐ Postmenopausal**
**DOB:**	_____ / _____ / _____	
**Zip Code:**	____ ____ ____ ____ ____	
**Date endocrine therapy initiated:**	_____ / _____ / _____	
**Planned Date Investigational Treatment Initiation:**	_____ / _____ / _____	
**Date of signed informed consent:**	_____ / _____ / _____	
**Patient ID for Neoadjuvant Trial:**		

REGISTRATION INCLUSION CRITERIA		
☐	1. Derived benefit from PD 0332991 in the neoadjuvant setting in this trial.	
☐	2. ECOG performance status of 0, 1 or 2	ECOG: __________<br>Date Assessed:________________
☐	3. If premenopausal, patient must be willing to comply with pregnancy requirements laid out in Section 5.5.	
	4. Laboratory Values (Within 14 days of Registration)	
☐	Leukocytes ≥ 3,000/mcL	Screening result:_____________
☐	Absolute Neutrophil Count (ANC) ≥ 1,500/mcL	Screening result:_____________
☐	Platelets (Plts) ≥ 100,000/mcL	Screening result:_____________
☐	Total bilirubin ≤ upper normal institutional limits	Screening result:_____________<br>ULN:_____________
☐	Alanine aminotransferase (ALT) and aspartate aminotransferase (AST) ≤ 2.5 x upper limit of normal (ULN)	ALT screening result:___________<br>ULN:____________<br>AST screening result:__________<br>ULN:_____________
☐	Serum creatinine ≤ upper normal institutional limits	Screening result:_____________<br>ULN:_____________
☐	5. Underwent surgery of the breast and axilla for curative intent. Date of Surgery: __________	
☐	6. At least 4 weeks post-completion of adjuvant chemotherapy and radiation therapy if indicated.	
☐	7. Patients who already started on adjuvant hormonal therapy are eligible under the following conditions:<br>a. For the 26 patients who enrolled in the initial cohorts and derived benefit from neoadjuvant PD 0332991 (C1D1 Ki67 >2.7% and C1D15 Ki67 ≤ 2.7%), adjuvant PD 0332991 should be initiated as soon as possible if adjuvant hormonal therapy has been initiated and the patient has completed radiation if indicated.<br>b. For patients who enrolled in the endocrine resistant cohort and derived benefit from neoadjuvant PD 0332991 (C1D15 Ki67 ≤ 10%), adjuvant PD 0332991 should be initiated within 6 months or sooner after initation of adjuvant hormonal therapy.	
☐	8. Able to understand and willing to sign an IRB-approved written informed consent document	

REGISTRATION EXCLUSION CRITERIA		
☐	1. Any of the following in the previous 6 months: myocardial infarction, severe/unstable angina, coronary/peripheral artery bypass graft, symptomatic congestive heart failure, cerebrovascular accident, transient ischemic attack, symptomatic pulmonary embolism	
☐	2. Uncontrolled intercurrent illness including, but not limited to ongoing or active infection, symptomatic congestive heart failure, unstable angina pectoris, uncontrolled symptomatic cardiac arrhythmia, psychiatric illness/social situations that would limit compliance with study requirements	
☐	3. Pregnant/nursing.	
☐	4. Unwilling to employ adequate contraception.	
☐	5. Known HIV-positive on combination antiretroviral therapy.	
☐	6. Known metastatic disease	
☐	7. Any condition that impairs patient's ability to swallow PD 0332991 tablets (*e.g.*, gastrointestinal tract disease resulting in an inability to take oral medication or a requirement for IV alimentation, prior surgical procedures affecting absorption)	
☐	8. History of allergic reactions attributed to compounds of similar chemical or biologic composition to PD 0332991 or other agents used in the study	
☐	9. Correct QT (QTc) interval > 470 msec	QTc:______________<br>Date: ______________
	10.	
☐	11. Current use or anticipated need for food or drugs that are known strong CYP3A4 inhibitors (i.e. grapefruit juice, verapamil, ketoconazole, miconazole, itraconazole, posaconazole, erythromycin, clarithromycin, telithromycin, indinavir, saquinavir, ritonavir, nelfinavir, lopinavir, atazanavir, amprenavir, fosamprenavir, nefazodone, diltiazem, and delavirdine) or inducers (i.e. dexamethasone, glucocorticoids, progesterone, rifampin, phenobarbital, St. John's wort).	
	12.	

Completed by: ____________________________ Date: _____ / _____ / _____

Investigator Signature: __________________________ Date: _____ / _____ / _____

For Wash U Use Only

Screen Failure ☐ Yes ☐ No

Subject Identifier: __ __ - __ __ __

Signature (Wash U designee): ____________________________ Date: _____ / _____ / _____

APPENDIX D: Medication Diary – PD 0332991

Today's Date: ___________________________ Agent: ____________________

Cycle: _________________________________

Patient Name: ___________________________ Study ID#: _________________

INSTRUCTIONS TO THE PATIENT:

1. Take 1 _____mg pill one time daily for 21 days, followed by 7 days of rest (no pills).
2. Take PD 0332991 at approximately the same time each day with a meal. Swallow the tablets whole and do not chew them.
3. If you forget to take your dose before 6:00PM, then do not take a dose that day. Restart taking it the next day.
4. Avoid St. John's Wort, Seville oranges, grapefruit, grapefruit juice, grapefruit hybrids, pummelos, and exotic citrus fruits from 7 days before you start taking PD 0332991 and throughout the entire study.
5. Complete this drug diary form for each 28-day cycle.
6. Record the date, time (include AM or PM), and the number of tablets taken (for each strength).
7. If you have any questions or notice any side effects, please record them in the comments section. Record the time if you should vomit.
8. If you have any questions, please call the study coordinator.
9. Please return the forms to your physician or your study coordinator when you go to your next appointment.
10. Please bring your unused study medications and/or empty bottles with you to each clinic visit so that a pill count can be done.

Day	Date	What time was dose taken?	# of 100 mg tablets taken	# of 25 mg tablets taken	Comments
1					
2					
3					
4					
5					
6					
7					
8					
9					
10					
11					
12					
13					
14					
15					
16					
17					
18					
19					
20					
21					

APPENDIX E: Medication Diary – Endocrine Therapy

Today's Date: ____________________________ Agent: ____________________

Cycle: __________________________________

Patient Name: ____________________________ Study ID#: _________________

INSTRUCTIONS TO THE PATIENT:

1. Complete one form for each month. Take _____mg (___tabets) of anastrozole at approximately the same time each day. Swallow the tablets whole and do not chew them.
2. Record the date, the number of tablets taken, and when you took them.
3. If you forget to take your dose before 6:00PM, then do not take a dose that day. Restart taking it the next day.
4. If you have any questions or notice any side effects, please record them in the comments section. Record the time if you should vomit.
5. Please return the forms to your physician or your study coordinator when you go to your next appointment. Please bring your unused study medications and/or empty bottles with you to each clinic visit so that a pill count can be done.

Day	Date	What time was dose taken?	# of tablets taken	Comments
1				
2				
3				
4				
5				
6				
7				
8				
9				
10				
11				
12				
13				
14				
15				
16				
17				
18				
19				
20				
21				
22				
23				
24				
25				
26				
27				
28				

APPENDIX F:
Pfizer Reportable
Event Cover Sheet

Investigator-Initiated Research
Reportable Event Fax Cover Sheet

Use this fax cover sheet to fax a Reportable Event for Investigator-Initiated Resear

Include with this form the completed Pfizer Investigator-Initiated Research Serious Adverse Event (IIR SAE) Form FDA 3500A-Mandatory Reporting, which can be obtained from the FDA website: www.fda.gov/medwa or other Pfizer agreed-upon form for SAE reporting.

If you are using the MedWatch Form to report, the following information should be included in block 5 of th section:

- **The complete clinical course of the patient receiving Pfizer drug**
- **The causality assessment for each Reportable Event**
- **The action taken for each study drug and for each Reportable Event**
- **The outcome for each Reportable Event**

This cover sheet MUST be provided with each completed SAE form. Do not substitute forms/reports or sub documentation other than what is required.

Do not fax these forms to any additional fax numbers other than the one listed

TO: *Pfizer U.S. Clinical Trial Department*			
FAX: ***1-866-997-8322***			
FROM:		DATE:	
TELEPHONE:		FAX:	
NUMBER OF PAGES (INCLUDING COVER SHEET):			
PRODUCT	PRODUCT NAME		
PFIZER REFERENCE NUMBER	TRACKING NUMBER	EXTERNAL REFERENCE NUMBER	EXTERNAL REFE
STUDY TITLE	*STUDY TITLE*		
PATIENT NUMBER			
INVESTIGATOR	INVESTIGATOR NAME, DEGREE		

Confidentiality Notice: The documents accompanying this telecopy transmission contain information belonging to Pfizer, which use of the addressee. If you are not the intended recipient, you are hereby notified that any disclosure, copying, distribution or the reliance on the contents of this telecopied information is strictly prohibited. If you have received this telecopy in error, please in telephone to arrange for the return of the original documents to us. Thank you.

FormCT25-USA01-10 Reportable Event Fax Cover S

APPENDIX H: Strong CYP3A4 Inhibitors or Inducers
(http://medicine.iupui.edu/clinpharm/ddis/clinical-table/)

Inhibitors
Indinavir
Nelfinavir
Ritonavir
Clarithromycin
Itraconazole
Ketoconazole
Nefazodone

Inducers
Carbamazepine
Efavirenz
Nevirapine
Phenobarbital
Phenytoin
Pioglitazone
Rifabutin
Rifampin
St. John's Wort
Troglitazone

PD 0332991

TPC BIOSPECIMEN SUBMISSION FORM

HRPO ID: 201301106 **Submitter's Institution:**______

Participant Study Number: ______ **Submitter Name (Last, First)**______

Participant Name (Initials): Last:______**First:**______**Middle:**______ **Submitter's Phone #:** ______

Study Time Points: ☐ Baseline ☐ Cycle 1 Day 1 (NeoAdj) ☐ Cycle 1 Day 15 ☐ Surgery ☐ Cycle 1 Day 1 (Adj) ☐ Cycle 12 Day 1 ☐ End of Cycle 23/EOT ☐ 1 Yr Post Tx ☐ 2 Yr Post Tx ☐ 3 Yr Post Tx ☐ 5 Yr Post Surgery ☐ Recurrence ☐ Other (Specify):______

Specimens Submitted: *CRA to provide Time & Date Collected, # of specimen and QTY (ml)/specimen if applicable. Include original form with kit shipment.*

Parent Label	Parent Type	Time Collected	Date Collected	Number / QTY	Pathological Status	Derivative Label	Derivative Type	# Aliquots	Storage Container	Position(s) (Row, Column)
	Whole Blood (EDTA)			ml	Non-Malignant		Frozen Cell Pellet			
							Plasma			
	Whole Blood (No Additive)			ml	Non-Malignant		Serum			
	Whole Blood (Streck)			ml	Non-Malignant		Plasma			
	Whole Blood (Streck)			ml	Non-Malignant		Plasma			
	Fixed Tissue			ea	Malignant	*Distribute Only*		NA		
	Fixed Tissue Block			ea	Malignant	*Storage Only*		NA		
Enter Label Range	Fixed Tissue Slide			ea	Malignant	*Storage Only*		NA		
Enter Label Range	Frozen Tissue Block			ea	Malignant	*Storage Only*		NA		
Enter Label Range	Plasma (EDTA-Frozen)			ml	Non-Malignant	*Storage Only*		NA		
Enter Label Range	Serum (No Additive-Frozen)			ml	Non-Malignant	*Storage Only*		NA		

Processing Notes:

1) Whole blood (EDTA) processed to plasma (3 x 1.5ml-all time points) and 3 frozen cell pellets (Cycle 1 Day 1 (NeoAdj) only) at central biorepository and stored.
2) Whole blood (No Additive) processed to serum (3 x 1.5ml) at central biorepository and stored.
3) Whole blood (Streck) processed to plasma (3 x 1.5ml, each tube) at central biorepository and stored.
4) Fixed tissue specimen (biopsy cores) received, accessioned and distributed to the CRA (or designee) within one business day.
5) Frozen tissue block(s), fixed tissue block, fixed tissue slides received at central biorepository and stored.
6) Frozen serum (3 x 1.5ml) and plasma (3 x 1.5ml) received by central biorepository and stored.

*** CONTROLLED DOCUMENT ***

This document is maintained electronically in the Tissue Procurement Core shared Quality Controlled Documents folder. It is the responsibility of the user to verify that any hard copy is of the latest version by checking the shared folder.
