# Supplementary material for: Biomarkers of response to neoadjuvant palbociclib plus anastrozole in endocrine-resistant estrogen receptor-positive/HER2-negative breast cancer: a phase 2 trial
Source: Nat Commun. 2026 Jan 27;17:949. doi: 10.1038/s41467-026-68570-6 (PMC12848104; doi:10.1038/s41467-026-68570-6)
Supplement: Supplementary file 3 — Reporting Summary [file 41467_2026_68570_MOESM3_ESM.pdf]

nature portfolio

Corresponding author(s): Cynthia X. Ma

Last updated by author(s): 12/19/2025

Reporting Summary

Nature Portfolio wishes to improve the reproducibility of the work that we publish. This form provides structure for consistency and transparency in reporting. For further information on Nature Portfolio policies, see our Editorial Policies and the Editorial Policy Checklist.

Statistics

For all statistical analyses, confirm that the following items are present in the figure legend, table legend, main text, or Methods section.

n/a	Confirmed	
☐	☒	The exact sample size (*n*) for each experimental group/condition, given as a discrete number and unit of measurement
☐	☒	A statement on whether measurements were taken from distinct samples or whether the same sample was measured repeatedly
☐	☒	The statistical test(s) used AND whether they are one- or two-sided<br>*Only common tests should be described solely by name; describe more complex techniques in the Methods section.*
☐	☒	A description of all covariates tested
☐	☒	A description of any assumptions or corrections, such as tests of normality and adjustment for multiple comparisons
☐	☒	A full description of the statistical parameters including central tendency (e.g. means) or other basic estimates (e.g. regression coefficient) AND variation (e.g. standard deviation) or associated estimates of uncertainty (e.g. confidence intervals)
☐	☒	For null hypothesis testing, the test statistic (e.g. *F*, *t*, *r*) with confidence intervals, effect sizes, degrees of freedom and *P* value noted<br>*Give P values as exact values whenever suitable.*
☒	☐	For Bayesian analysis, information on the choice of priors and Markov chain Monte Carlo settings
☒	☐	For hierarchical and complex designs, identification of the appropriate level for tests and full reporting of outcomes
☐	☒	Estimates of effect sizes (e.g. Cohen's *d*, Pearson's *r*), indicating how they were calculated

Our web collection on statistics for biologists contains articles on many of the points above.

Software and code

Policy information about availability of computer code

Data collection	No software was utilized for data collection.
Data analysis	Rstudo (v4.5.1) and the following packages were utilized: GSEA software (v4.2.2), somaticwrapper (v2.2), Strelka (v2.9.2), Mutect (v1.1.7), Mutect2 (v4.1.2.0), VarScan (v2.3.8), Pindel (v0.2.5) survival (v3.6-4), survminer (v0.4.9), DESeq2 (v1.48.2), limma (v3.64.3), pROC (v1.19.0.1). R code is provided in the supplementary data.

For manuscripts utilizing custom algorithms or software that are central to the research but not yet described in published literature, software must be made available to editors and reviewers. We strongly encourage code deposition in a community repository (e.g. GitHub). See the Nature Portfolio guidelines for submitting code & software for further information.

Data

Policy information about availability of data

All manuscripts must include a data availability statement. This statement should provide the following information, where applicable:

- Accession codes, unique identifiers, or web links for publicly available datasets
- A description of any restrictions on data availability
- For clinical datasets or third party data, please ensure that the statement adheres to our policy

RNAseq and WES data generated in this study have been deposited in the Genome Sequencing Archive (GSA), under the accession code HRA009522 [https://ngdc.cncb.ac.cn/gsa-human/browse/HRA009522]. Access is restricted to qualified researchers and requires approval by the NGDC Data Access Committee based on a request consistent with participant consent, institutional ethics approval, and data use restrictions, including prohibition of re-identification. Access decisions are

typically made within 2–4 weeks, access is granted for a defined research purpose and duration, and the data will remain available under controlled access in accordance with participant consent and applicable regulations. Mass spectrometry proteomic data generated in this study have been deposited in the ProteomeXchange via the PRIDE database, under the accession code PXD058250. Microarray data utilized in this study from NeoPalAna initial cohort under the accession code GSE93204 [https://www.ncbi.nlm.nih.gov/geo/query/acc.cgi?acc=GSE93204]. Transcriptomic data from the Real-world" external cohort were from source publication31. De-identified individual participant data underlying the results reported in this article will be shared with qualified researchers upon reasonable request, subject to institutional approval, a data use agreement, and compliance with patient consent and privacy regulations.

Research involving human participants, their data, or biological material

Policy information about studies with human participants or human data. See also policy information about sex, gender (identity/presentation), and sexual orientation and race, ethnicity and racism.

Reporting on sex and gender	While both biological men and women were eligible for this trial, breast cancer rates in men are quite low. For the purposes of this trial, only biological women met the eligibility criteria and were enrolled. Because of this, no sex- or gender-based analyses were performed. Patients consented to sharing of individual-level data.
Reporting on race, ethnicity, or other socially relevant groupings	Patients were not excluded from participation in this study based on race, ethnicity or any other socially relevant construct, as breast cancer affects biological men and women of all races/ethnicities. All race and ethnicity data collected was self-reported by each enrolling patient. Data presented in this manuscript was blinded to race, ethnicity, and all other socially relevant constructs.
Population characteristics	All patients determined to be eligible for this study based on the protocol defined inclusion and exclusion criteria, will be started on treatment. For analysis purposes, prior diagnoses and treatment history will be obtained.
Recruitment	Patients at the Washington University in St. Louis School of Medicine were recruited specifically from the Siteman Cancer Center outpatient population or patient referrals by our community oncologists to the Principal Investigator (PI) and co-investigators. The recruitment process did not involve any restrictions based on social or demographic factors including age or racial/ethnic characteristics of the subject population outside of the protocol defined exclusion criteria.
Ethics oversight	The study protocol was approved by the Washington University in St. Louis School of Medicine Institutional Review Board (IRB) as part of the Human Research Protection Office (HRPO).

Note that full information on the approval of the study protocol must also be provided in the manuscript.

Field-specific reporting

Please select the one below that is the best fit for your research. If you are not sure, read the appropriate sections before making your selection.

- [x] Life sciences
- [ ] Behavioural & social sciences
- [ ] Ecological, evolutionary & environmental sciences

For a reference copy of the document with all sections, see nature.com/documents/nr-reporting-summary-flat.pdf

Life sciences study design

All studies must disclose on these points even when the disclosure is negative.

Sample size	The primary endpoint was complete cell cycle arrest (CCCA), defined as a Ki67< 2.7% on the drug combination at Cycle 1 Day 15. A Simon optimal two stage phase II clinical trial design was employed to allow a 90% chance of detecting a CCCA of ≥20% (alpha of 0.1). The regimen was considered to have sufficient anti-tumor activity if 4 out of 37 enrolled patients (12 in stage 1 and 25 in stage 2) achieved CCCA. The study was stopped after enrolling 34 patients as the primary endpoint had already been met with the smaller sample size, per the data safety and monitoring board
Data exclusions	No data were actively excluded. One patient had missing data due to insufficient tumor.
Replication	Data replication was not relevant for the clinical trial.<br>For the validation experiments, biological replicates are described in the figure legends.
Randomization	There was no randomization in this trial. All participants received the same treatment.
Blinding	Study participants were not blinded in this trial.

Reporting for specific materials, systems and methods

We require information from authors about some types of materials, experimental systems and methods used in many studies. Here, indicate whether each material, system or method listed is relevant to your study. If you are not sure if a list item applies to your research, read the appropriate section before selecting a response.

Materials & experimental systems

n/a	Involved in the study	
☐	☒	Antibodies
☐	☒	Eukaryotic cell lines
☒	☐	Palaeontology and archaeology
☒	☐	Animals and other organisms
☐	☒	Clinical data
☒	☐	Dual use research of concern
☒	☐	Plants

Methods

n/a	Involved in the study	
☒	☐	ChIP-seq
☒	☐	Flow cytometry
☒	☐	MRI-based neuroimaging

Antibodies

Antibodies used: Antibody information are provided in supplementary 10.

Validation: Antibodies are validated as per manufacturer's website. ISG15 antibody was validated as in Lenschow et al. PNAS 2007

Eukaryotic cell lines

Policy information about cell lines and Sex and Gender in Research

Cell line source(s): MCF7

Authentication: MCF7 was acquired from ATCC

Mycoplasma contamination: Cells were routinely tested for mycoplasma

Commonly misidentified lines (See ICLAC register): No commonly misidentified lines were utilized.

Clinical data

Policy information about clinical studies

All manuscripts should comply with the ICMJE guidelines for publication of clinical research and a completed CONSORT checklist must be included with all submissions.

Clinical trial registration: NCT01723774

Study protocol: The NeoPalAna protocol is provided as supplementary material.

Data collection: All participants gave written informed consent under the IRB-approved protocol prior to inclusion in the study, including access to archival tumor tissue for research. The study was approved by the Institutional Review Board (IRB) and conducted in compliance with the Declaration of Helsinki and Good Clinical Practice guidelines. Recruitment for the trial began in January 2016. Thirty-four patients were enrolled between August 2016 and March 2021.

Outcomes: The primary outcome of this study was to determine the rate of complete cell cycle arrest, defined by Ki67 <2.7%, following 2 weeks of neoadjuvant palbociclib in combination with anastrozole in women with clinical stage II or III ER+/HER2- endocrine-resistant breast cancer. Secondary outcomes included response rate and safety profile. Exploratory outcomes include molecular mechanisms of response of the study drug by tumor genomic, transcriptomic and proteomic analysis.

Plants

Seed stocks: No seed stocks were utilized in this study.

Novel plant genotypes: No novel plant genotypes were utilized in this study.

Authentication: No plants were utilized in this study.
