# Supplementary material for: Biomarkers of response to neoadjuvant palbociclib plus anastrozole in endocrine-resistant estrogen receptor-positive/HER2-negative breast cancer: a phase 2 trial
Source: Nat Commun. 2026 Jan 27;17:949. doi: 10.1038/s41467-026-68570-6 (PMC12848104; doi:10.1038/s41467-026-68570-6)
Supplement: Supplementary file 4 — Description of Additional Supplementary Files [file 41467_2026_68570_MOESM4_ESM.docx]

**Description of Additional Supplementary Files:**

Supplementary Data 1. Tumor whole exome sequencing (WES) analysis in tumors and/or with matched normal.

Supplementary Data 2. Differential copy number variants (CNV) in Resistant vs Sensitive tumors.

Supplementary Data 3. Baseline differentially expressed genes by RNA-seq in Resistant vs. Sensitive tumors.

Supplementary Data 4. C1D15 differentially expressed genes by RNA-seq in Resistant vs. Sensitive tumors.

Supplementary Data 5. Correlation between gene expression and Ki67 in Sensitive or Resistant tumors at baseline and C1D15.

Supplementary Data 6. Treatment induced changes in gene expression between BL and C1D15 in Sensitive and Resistant samples.

Supplementary Data 7. Predictive performance of 33 gene candidates.

Supplementary Data 8. Bootstrap analysis of the derived AUC of 33-gene signature in the NeoPalAna ET-R dataset.
