# Supplementary figures and images for: Biomarkers of response to neoadjuvant palbociclib plus anastrozole in endocrine-resistant estrogen receptor-positive/HER2-negative breast cancer: a phase 2 trial
Source: Nat Commun. 2026 Jan 27;17:949. doi: 10.1038/s41467-026-68570-6 (PMC12848104; doi:10.1038/s41467-026-68570-6)

Fig. 5g

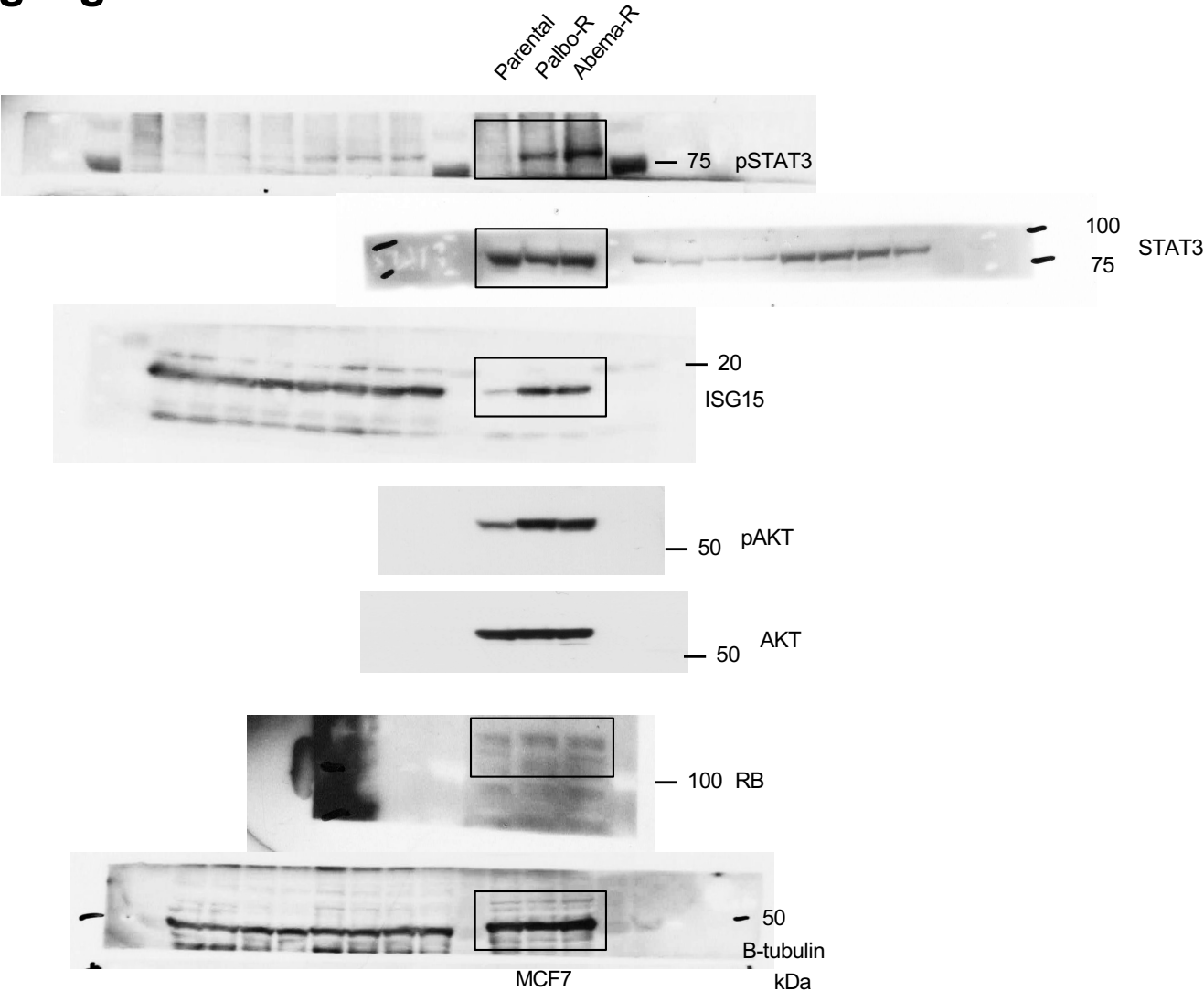

Fig. 5i

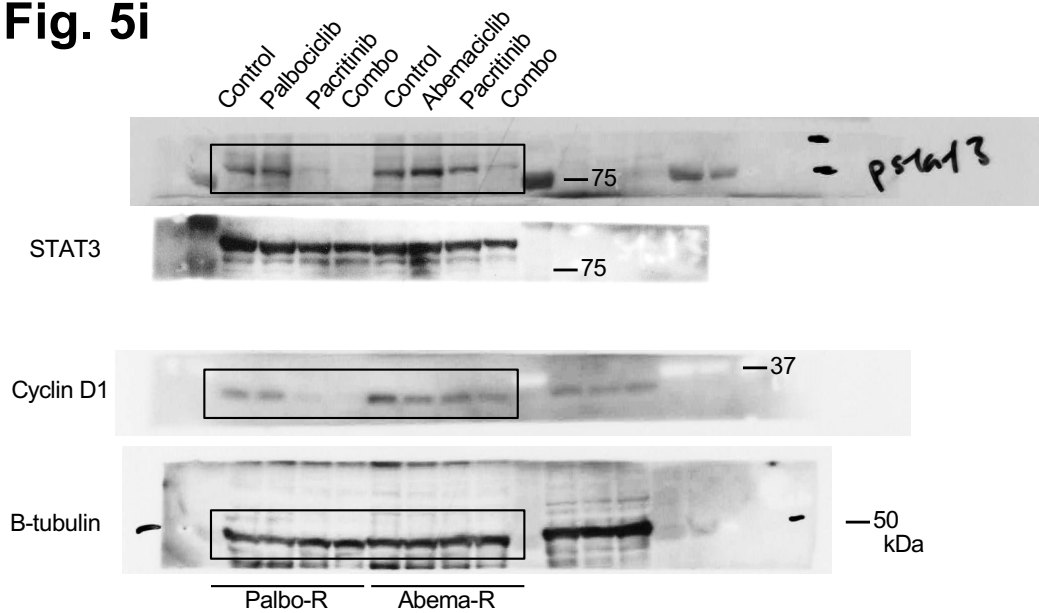

Supplement: Supplementary file 13 — Source Data [file 41467_2026_68570_MOESM13_ESM.zip › Source Data western.pdf]
